# Substitution Mapping and Allelic Variations of the Domestication Genes from *O. rufipogon* and *O. nivara*

**DOI:** 10.1186/s12284-023-00655-y

**Published:** 2023-09-05

**Authors:** Zhangqiang Wang, Zisheng Guo, Tuo Zou, Zhe Zhang, Jianan Zhang, Ping He, Ruifeng Song, Ziqiang Liu, Haitao Zhu, Guiquan Zhang, Xuelin Fu

**Affiliations:** 1 Guangdong Provincial Key Laboratory of Plant Molecular Breeding, College of Agriculture, South China Agricultural University, Guangzhou, 510642 China; 2 College of Life Sciences, South China Agricultural University, Guangzhou, 510642 China

**Keywords:** Single segment substitution lines, Domestication gene, Substitution mapping, Allelic variation, *O. rufipogon*, *O. nivara*

## Abstract

**Background:**

Domestication from wild rice species to cultivated rice is a key milestone, which involved changes of many specific traits and the variations of the genetic systems. Among the AA-genome wild rice species, *O*. *rufipogon* and *O*. *nivara*, have many favorable genes and thought to be progenitors of *O. sativa*.

**Results:**

In the present study, by using *O. rufipogon* and *O. nivara* as donors, the single segment substitution lines (SSSLs) have been developed in the background of the elite indica cultivar, HJX74. In the SSSLs population, 11 genes for 5 domestication traits, including tiller angle, spreading panicle, awn, seed shattering, and red pericarp, were identified and mapped on 5 chromosomes through substitution mapping. Herein, allelic variations of 7 genes were found through sequence alignment with the known genes, that is, *TA7-RUF* was allelic to *PROG1*, *TA8-RUF* was allelic to *TIG1*, *SPR4-NIV* was allelic to *OsLG1*, *AN4-RUF* was allelic to *An-1*, *SH4-NIV* was allelic to *SH4*, and both *RC7-RUF* and *RC7-NIV* were allelic to *Rc*. Meanwhile, 4 genes, *TA11-NIV*, *SPR3-NIV*, *AN3-NIV,* and *AN4-NIV*, were considered as the novel genes identified in these SSSLs, because of none known genes for the related domestication traits found in the chromosomal locations of them.

**Conclusion:**

The results indicated that the SSSLs would be precious germplasm resources for gene mining and utilization from wild rice species, and it laid the foundation for further analyses of the novel domestication genes to better understand the genetic basis in regulating the traits variation during domestication.

**Supplementary Information:**

The online version contains supplementary material available at 10.1186/s12284-023-00655-y.

## Background

Rice (*Oryza sativa* L.) is the staple food for more than half of the population in the world. During the last decades, great achievements in grain yield improvement of per unit area in rice have guaranteed the supply of rice food. These successes have no doubt mainly benefited from rice semi-dwarf breeding in the 1950s and the utilization of the heterosis of hybrid rice in the 1970s. These two major breeding breakthroughs are inseparable from the contribution of the beneficial genes, that is, the utilization of semi-dwarfing gene *sd1* from the landraces (Sasaki et al. 2002) and the wild abortive cytoplasmic male sterile (CMS) genes from wild rice species, *O. rufipogon* (Luo et al. 2013a). With the need and the greatest challenges of ending world hunger, it is obvious that both yield and quality improvement remains the main targets in rice breeding whether now or in the future. Therefore, both enrichment of the germplasm pools and mining the beneficial genes are much more indispensable for us. As is known to all, wild relatives of crops are valuable sources of novel alleles that were lost during the domestication process (Tanksley and McCouch 1997), thus rediscovery of the natural genes from wild species has drawn more and more attentions from rice breeders over these years. Interspecific breeding is an important way of expanding the gene pools and enlarging the genetic variations available for rice improvement. In *Oryza* genus, there are more than 20 wild species, including 6 AA-genome wild rice species, namely *O. rufipogon, O. nivara, O. longistaminata, O. meridionalis*, *O. glumaepatula*, and *O. barthi*, which are important reservoirs of beneficial genes for rice breeding (Brar and Khush 1997; Khush 1997). Besides, interspecific gene flow could be used in enhancing the effectiveness and efficiency of conservation method for wild rice species (Banaticla et al. 2022). *O. rufipogon* is considered as the most popular and influential wild rice species in the gene introgression to the cultivated rice through interspecific hybridization, while *O. nivara* belongs to the same complex with *O. rufipogon*. Both of them are the most closely related species to *O. sativa* and thought to be the direct progenitors of *O. sativa* (Oka 1988; Sweeney et al. 2007; Yamanaka et al. 2003). However,* O. nivara* is obviously differentiated from *O. rufipogon* in certain cases, such as a number of traits related to plant adaptation, life history, mating system, and flowering time, etc. (Grillo et al. 2009). Although both *O. rufipogon* and *O. nivara* are important for the innovation of rice genetic resources, attentions mainly focused on *O. rufipogon*, particularly in the traits of heading date (Furuta et al. 2014), salt stress tolerance (Ding et al. 2022), resistance to rice blast (Hirabayashi et al. 2010), and yield-related components (Tian et al. 2006; Yuan et al. 2009). Notably, it has made progress in development of genetic populations and mapping of genes/QTLs in *O. nivara* in these years, such as Chromosome segment substitution lines (CSSLs) and Introgression lines (ILs) (Furuta et al. 2016; Liu et al. 2021; Ma et al. 2016; Zhang et al. 2022).

During the long domestication process of *O. sativa* from wild rice species, artificial evolutionary forces have made some characters of wild rice species lost to satisfy the human needs of cultivation, such as prostrate growth habit, spreading panicle, seed shattering, long awn, black husk, red pericarp, and so on. Recently, several rice domestication genes have been uncovered, and consequently, the unlocked genetic bases for the domestication traits have greatly enhanced our understanding of the underlying cause of rice domestication. For example, the erect growth habit in cultivated rice is transited from the prostrate growth habit of wild rice species, which is one of the key events in rice domestication. In rice cultivation, the erect growth habit of the cultivated rice could increase planting density and grain yield (Tan et al. 2008). Besides, long and bared awns are beneficial for the seed dispersal and avoidance of bird pecking in wild rice species. Nevertheless, awns cause inconvenience for seed harvesting and processing s in rice. Additionally, seed shattering is a protective trait for wild rice plants, but an undesired trait in crops. Loss of seed shattering is one of the key domestication traits in rice, which is advantageous for rice harvesting and yield improvement. It is known that domestication of any trait in crops is mainly related with the intrinsic genetic variations. Nowadays, several domestication genes have been cloned from wild rice species. For instance,, *Sh4*/*SHA1* for seed shattering (Li et al. 2006), *PROG1* (Jin et al. 2008; Tan et al. 2008) and *TIG1* (Zhang et al. 2019) for prostrate growth habit/tiller angle, and awn genes, *An-1* (Luo et al. 2013b), *LABA1* (Hua et al. 2015), *An-2* (Gu et al. 2015), and *GAD1* (Jin et al. 2016), and *OsLG1* for spreading panicle (Ishii et al. 2013; Zhu et al. 2013), as well as *Rc* for red pericarp (Sweeney et al. 2006). Anyway, the known genes related with domestication traits are not enough for revealing the genetic mechanism of rice domesticationcompletely, it needs to discover many a gene and mine alleles in a large scale.

It has been widely proven that CSSLs and ILs are excellent innovative germplasm resources, demonstrating the superiority in the identification of natural favorable and untapped new alleles in wild species (Ali et al. 2010). Recently, we have developed a set of single segment substitution lines (SSSLs) of AA-genome wild rice species, *O. meridionalis*, *O. glumaepatula*, and *O. barthi*, in the genetic background of indica cultivar, HJX74 (He et al. 2017; Zhao et al. 2019). And based on the SSSLs, we have identified dozens of QTLs related to agronomic traits, grain yield, and the abiotic stress tolerance, and the SSSLs showed the greatest potential in gene/QTL identification, fine-mapping, allelic analysis, and in pyramiding breeding from the previous researches (Fang et al. 2019; Tan et al. 2020; Wang et al. 2020; Zou et al. 2020). In the present study, we developed SSSLs populations from another two AA-genome wild rice species, *O. rufipogon* and *O. nivara*, in the background of HJX74, and we investigated the domestication traits and mapped the related genes, as well as analyzed the allelic variations. A total of 11 genes were mapped through substitution mapping in the SSSLs, herein, allelic variations of 7 genes were found through sequence alignment, and 4 genes were considered as novels. The results provided the reliable evidence for the effectively transformation of the genes from *O. rufipogon* and *O. nivara* into the SSSLs. Moreover, the novel domestication genes identified from the SSSLs would inspire us to explore the possible different ways and mechanisms for the trait domestication in rice.

## Results

### Development and Characterization of the SSSLs Populations

**Fig. 1:**
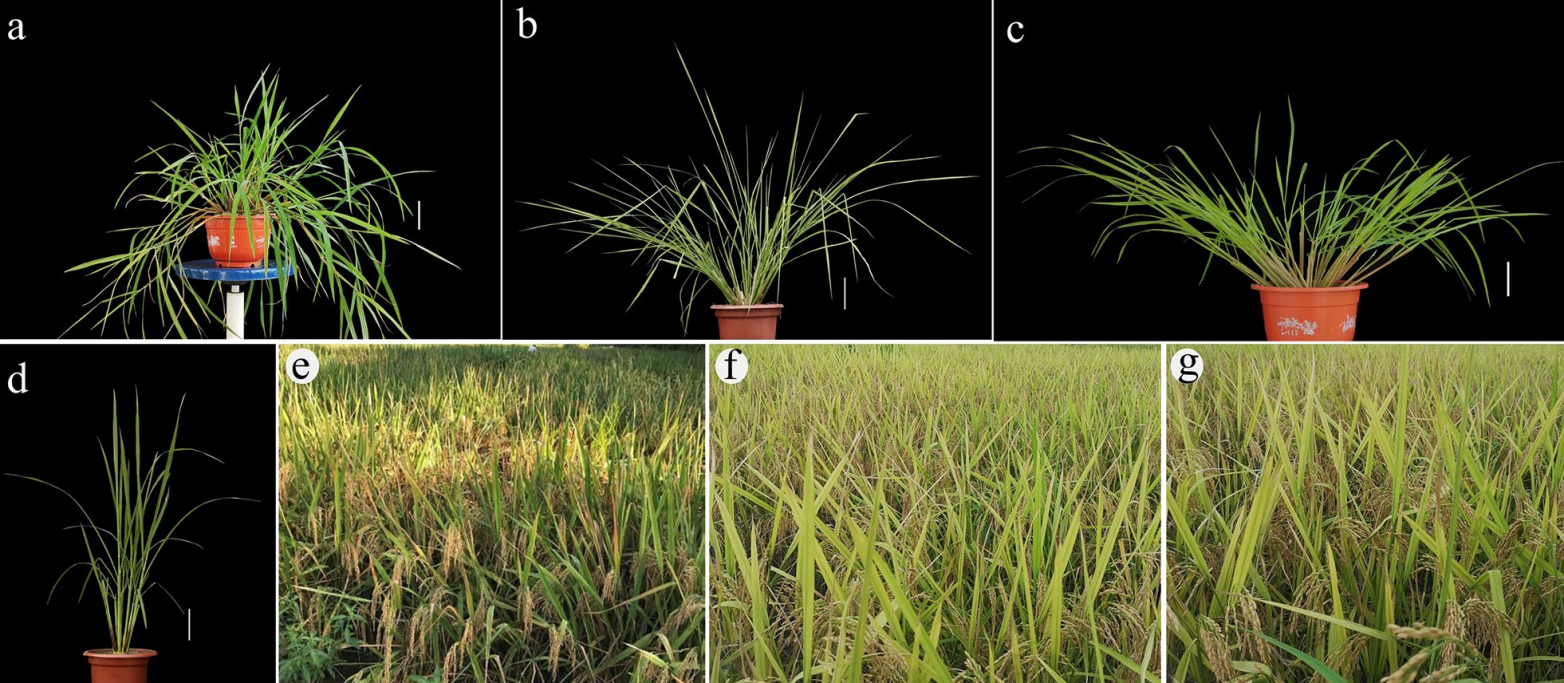
The SSSLs populations and the plants of the recipient and donor parents. **a** *O. rufipogon*, (*RUF*, IRGC106149); **b** *O. nivara*, (*NIV1*, IRGC104309); **c** *O. nivara*, (*NIV2*, IRGC105919); **d** HJX74; **e** SSSLs population (RUF-SSSLs) from *RUF*; **f** SSSLs population (NIV1-SSSLs) from *NIV1*; **g** SSSLs population (NIV2-SSSLs) from *NIV2*. Bar = 10 cm

**Fig. 2:**
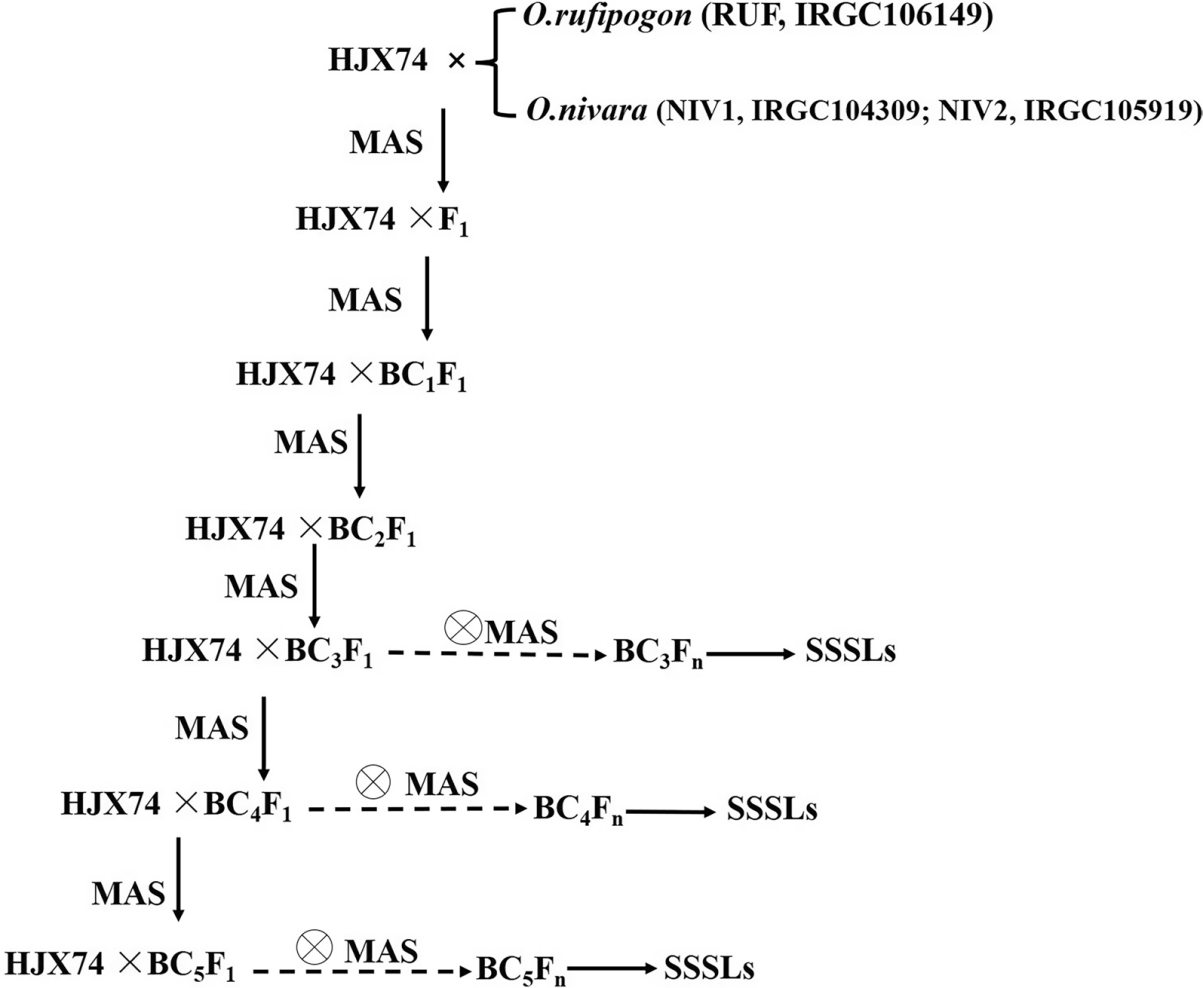
Flow chart of the SSSLs development process from *O. rufipogon* and *O. nivara*. MAS- marker assisted selection. n = 2 ~ 4

**Fig. 3:**
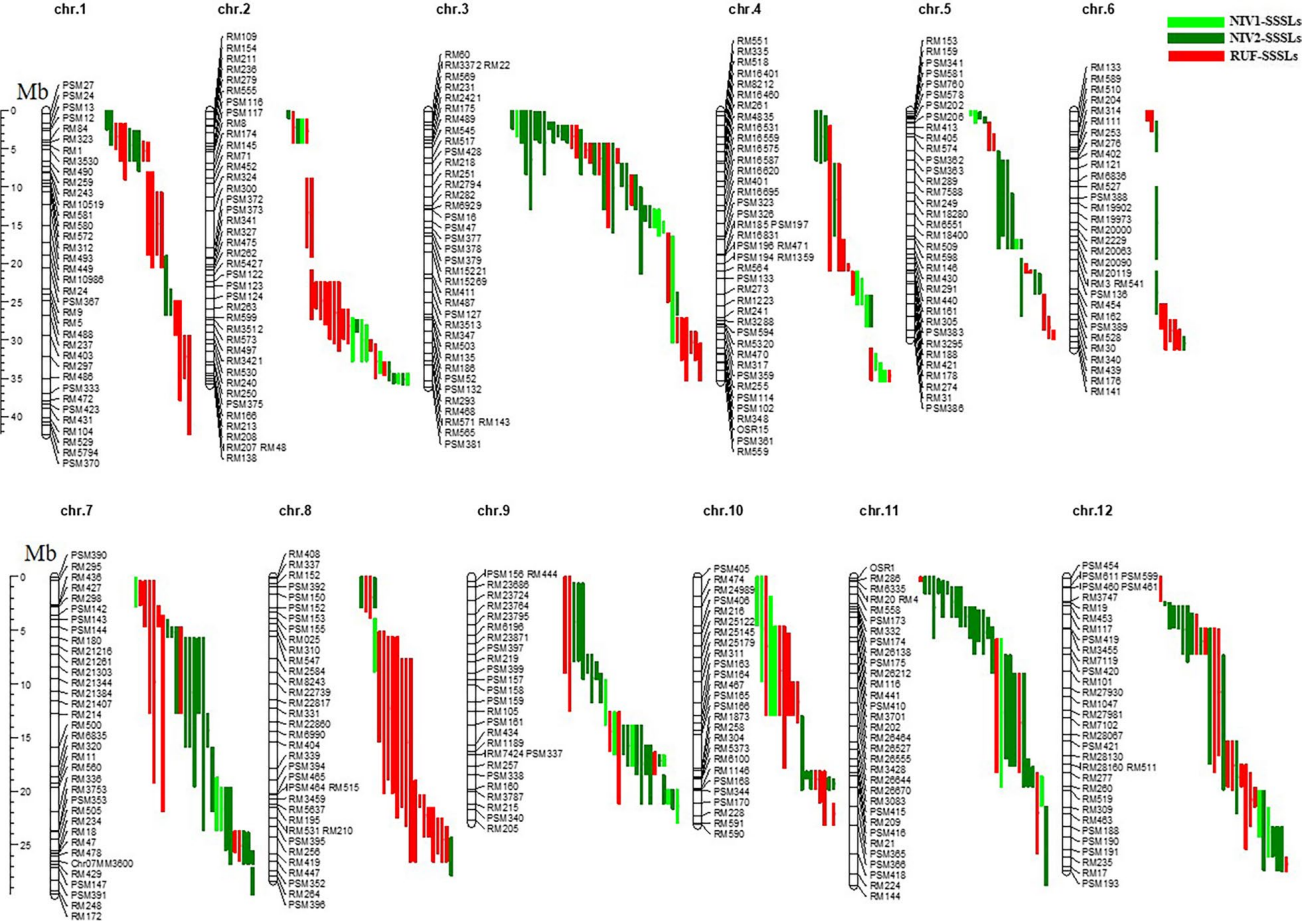
The physical map of the substituted segments distributing on chromosomes in the three sets of SSSLs. The light green, dark green and red bars at the right side of each chromosome represent the substituted segments in NIV1-SSSLs, NIV2-SSSLs and RUF-SSSLs, respectively

**Fig. 4:**
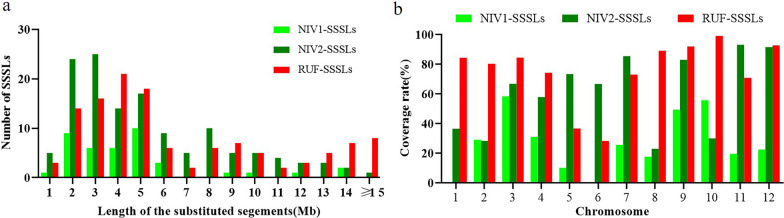
Length distribution and genome coverage of the substituted segments in the SSSLs populations. **a** Length distribution of the substituted segments in the three sets of SSSLs; **b** the coverage rate of the three sets of SSSLs. The light green, dark green, and red columns indicate the different SSSLs, respectively

During the development of SSSLs, the crosses between HJX74 and the wild rice species, the successive backcrosses and self-crosses were conducted by using HJX74 as recipient, and *RUF*, *NIV1*, and *NIV2* as donors (Fig. 1a–d). Besides, 265, 269, and 284 polymorphic markers between HJX74 and *NIV1*, HJX74 and *NIV2*, and HJX74 and *RUF* were selected, respectively. The markers were evenly distributed on 12 chromosomes (Additional files 1 and 2). And then, the polymorphic markers were used in the detection of the substituted segments since the generation of BC_3_ (Fig. 2). A total of 295 SSSLs were ultimately developed in the background of HJX74, including 123 RUF-SSSLs from *RUF* and 172 NIV-SSSLs from *NIV* (39 NIV1-SSSLs and 133 NIV2-SSSLs) (Fig. 1e–g). The substituted segments in the SSSLs populations were distributed on 12 chromosomes (Fig. 3), and the detailed information of each SSSL was shown in Additional file 3. The genetic constitutions of the SSSLs populations were briefly analyzed. In RUF-SSSLs population, the total length of the substituted segments was 761.68 Mb with a 6.19 Mb mean length for each substituted segment. The coverage length of the substituted segments was 279.83 Mb which covered 75.49 percent of the rice genome. While in NIV1-SSSLs population, the total length of the substituted segments was 166.05 Mb with a 4.26 Mb mean length for each substituted segment. And the coverage length of the substituted segments was 94.38 Mb, covering 26.56 percent of the rice genome. In addition, in NIV2-SSSLs population, the total length of the substituted segments was 638.04 Mb with a 4.80 Mb mean length for each substituted segment. And the coverage length of the substituted segments was 224.11 Mb, covering 61.37 percent of the rice genome (Additional file 4, Fig. 4). Additionally, in all of the 295 SSSLs from the three donors, the total length and the coverage length of the substituted segments were 1565.77 Mb and 598.32 Mb, respectively. And the mean length of each substituted segment was 5.31 Mb. It showed that more than 58% in RUF-SSSLs, 80% in NIV1-SSSLs and 64% in NIV2-SSSLs of the substituted segments were shorter than 5.0 Mb, respectively; while approximately 20.32% in RUF-SSSLs, 7.5% in NIV1-SSSLs and 9.85% in NIV2-SSSLs of the substituted segments were longer than 10 Mb (Fig. 4a). Moreover, the coverage rate of the substituted segments for rice genome was 163.42% based on the coverage length of all the 295 SSSLs (Additional file 4). However, the coverage rate was uneven among chromosomes, which varied from 95.10% on chromosome 6 to 224.57% on chromosome 9 (Fig. 4b). In summary, a total of 295 SSSLs from *O. rufipogon* and *O. nivara* have been developed in this study, each SSSL contained a small chromosomal substituted segment from the donors in the genetic background of HJX74. And each SSSL and HJX74 could be genetically considered a pair of near-isogenic line (NIL) for gene identification, mapping, cloning and functional analysis.

### Substitution Mapping and Allelic Variations of the Domestication Genes

**Fig. 5:**
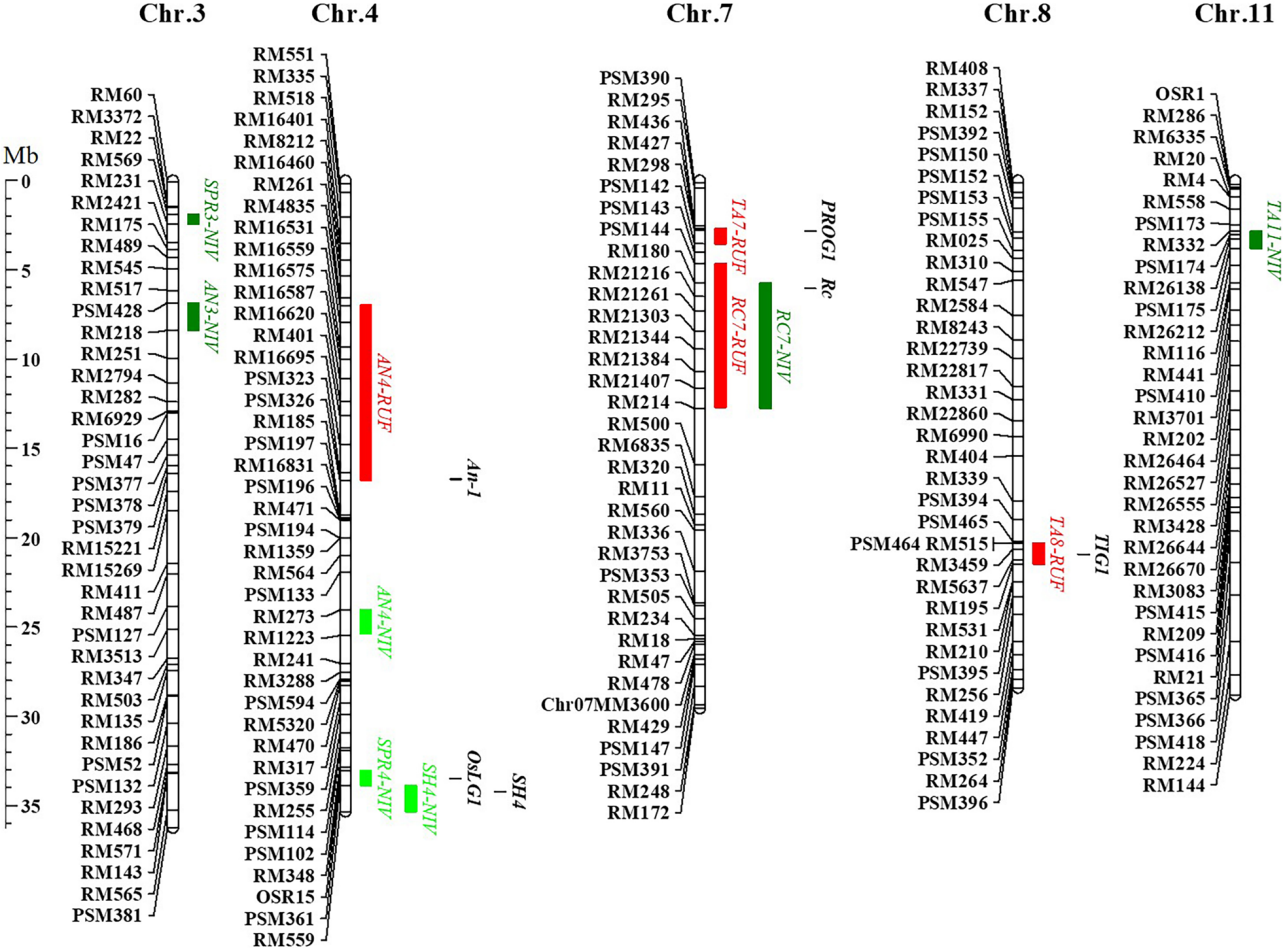
Map locations of the genes associated with the domestication traits. The light green, dark green, and red columns represent the genes are transformed from the donors of *NIV1*, *NIV2*, and *RUF*, respectively. The black bars mean the position of the known genes

Five domestication traits were observed in the SSSLs population, that is, prostrate growth habit or described as large tiller angle, spreading panicle, awn, seed shattering, and red pericarp. And 11 related genes were identified on 5 chromosomes and delimited to the overlapped intervals of the substituted segments through substitution mapping (Fig. 5). Furthermore, allelic variations of the domestication genes were analyzed by comparison of the chromosome interval and sequence alignment. There were 7 genes showed allelic variations with the known genes (Table 1), and 4 genes were suggested the novel genes identified at present. It demonstrated that some domestication genes in the two wild rice donors have been transformed into the SSSLs. The results were concisely described as follows.

**Table 1:** Domestication genes detected in RUF-SSSLs and NIV-SSSLs

Trait	SSSLs	Identified genes	Chr	Region	Known genes	References
Tiller angle	SR59, SR60, SR61, SR63	*TA7-RUF*	7	RM515-RM195	*PROG1*	Jin et al. (2008); Tan et al. (2008)
	SR72, SR77, SR76, SR75, SR78, SR79, SR80	*TA8-RUF*	8	RM515-RM195	*TIG1*	Zhang et al. (2019)
	SN136, SN139, SN143	*TA11-NIV*	11	RM332-PSM175	*–*	–
Spreading panicle	SN22, SN28, SN29, SN33	*SPR3-NIV*	3	RM569-RM231	–	–
	SN57, SN58	*SPR4-NIV*	4	OSR15-PSM361	*OsLG1*	Zhu et al. (2013)
Awn	SN34, SN39	*AN3-NIV*	3	PSM428-RM218	–	–
	SN54, SN56	*AN4-NIV*	4	RM273-RM252	–	–
	SR118, SR119	*AN4-RUF*	4	RM4835-PSM326	*An-1*	–
Seed shattering	SN58	*SH4-NIV*	4	PSM361-RM559	*SH4*	Li et al. (2006)
Red pericarp	SR64, SR65	*RC7-RUF*	7	PSM144-RM214	*Rc*	Sweeney et al. (2006)
	SN79, SN82, SN85	*RC7-NIV*	7	RM180-RM6728		

### Tiller Angle

**Fig. 6:**
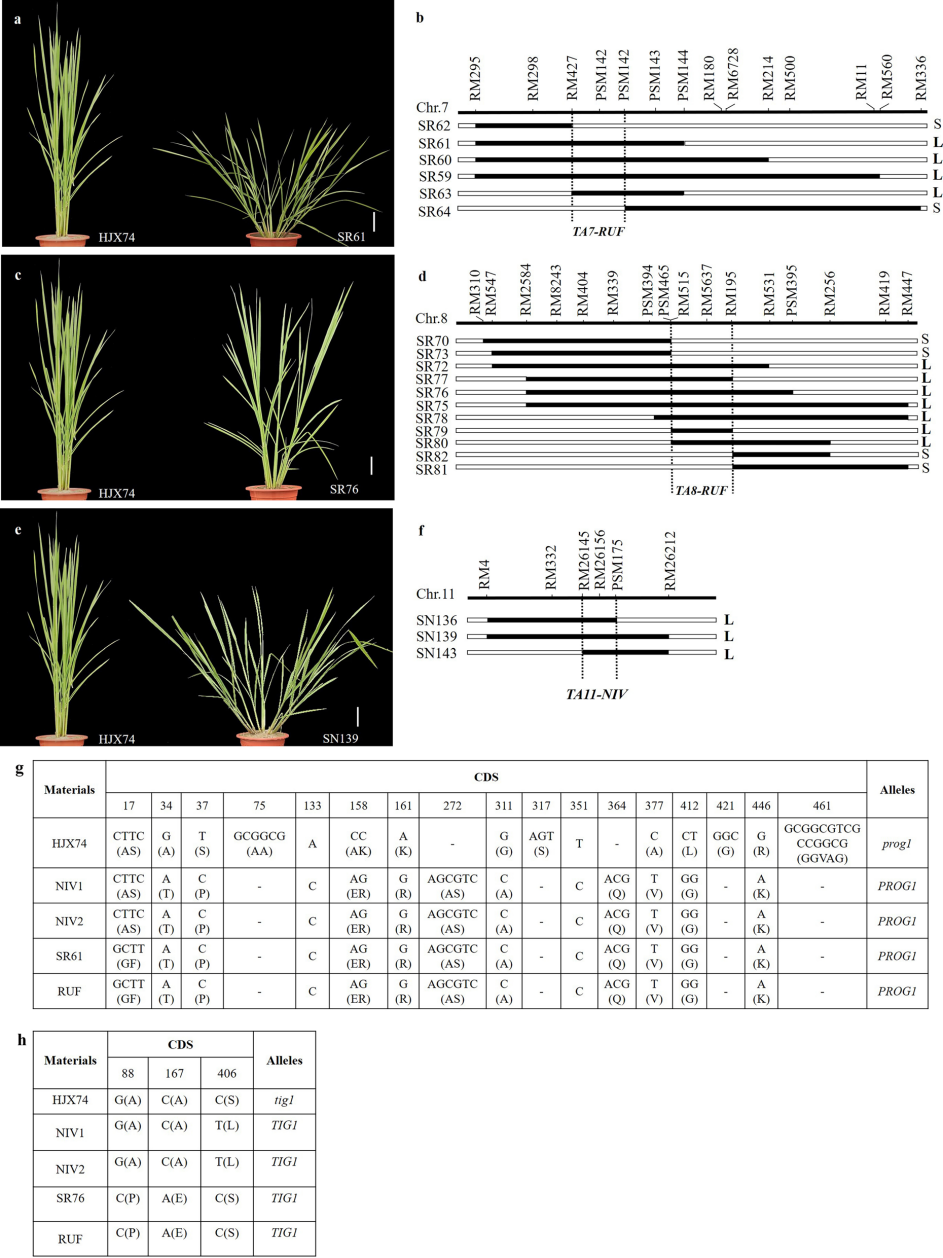
Phenotype and the substitution mapping of the tiller angle genes in SSSLs. **a**, **c**, and **e** show the phenotypes of the tiller angles in HJX74 and SSSLs, bar = 10 cm; **b**, **d**, and **f** show the substitution mapping of the tiller angle genes; **g** shows the variations of CDS sequence of *PROG1*; **h** shows the DNA variations of CDS sequence of *TIG1*. L, large tiller angle; S, small tiller angle

Among the SSSLs, we observed the obviously larger tiller angles in 14 SSSLs than the recipient (HJX74), accordingly, 3 genes related with the large tiller angles, *TA7-RUF*, *TA8-RUF*, and *TA11-NIV,* were identified and mapped (Fig. 6). Briefly, 4 RUF-SSSLs, SR59, SR60, SR61, and SR63, showed the larger tiller angles, while the other 2 RUF-SSSLs, SR62 and SR64, displayed the smaller tiller angles similar to HJX74. Due to these 6 SSSLs all carried the substituted segments on chromosome 7, through substitution mapping among them, the gene, *TA7-RUF*, for the larger tiller angle was identified and narrowed down to a 0.87 Mb interval between markers RM427 and PSM142 on chromosome 7 (Fig. 6a, b). Similarly, another larger tiller angle gene, *TA8-RUF***,** was mapped to a 1.19 Mb target region between markers RM515 and RM195 on chromosome 8 in RUF-SSSLs with the overlapped substituted segments (Fig. 6c, d). Additionally, *TA11-NIV* for the larger tiller angle was delimited to a 0.98 Mb region between RM26145 and PSM175 on chromosome 11 in 3 NIV-SSSLs, SN136, SN139, and SN143 (Fig. 6e, f). Furthermore, based on the rice reference genome (IRGSP1.0), the larger tiller angle gene, *TA7-RUF*, mapped between the markers RM427 and PSM142 with the physical position of 2,679,666–3,555,628 bp on chromosome 7 covered the region of the popular prostrate growth habit gene, *PROG1* (2,839,476–2,839,979 bp). *PROG1* was cloned from the introgression line of *O. rufipogon* and the allele of *O. rufipogon* showed the positive effect for prostrate growth habit of the introgression line (Jin et al. 2008; Tan et al. 2008). Through the sequence alignment of the coding sequence (CDS) of *PROG1* in SR61(carried *TA7-RUF*), *O. rufipogon* (IRGC106149)*,* and HJX74, it showed that SR61 and *O. rufipogon* had the same sequence, however, variations of 16 single nucleotide polymorphisms (SNPs) and 6 insertion/deletions (InDels) were found in HJX74 (Fig. 6g). By comparing the *PROG1* sequences of SR61, HJX74, *O. rufipogon* (IRGC106149) with YJCWR (Yuanjiang common wild rice, *O. rufipogon*) and Teqing (Tan et al. 2008), we found that 12 SNPs and 6 Indels were identical in SR61 and YJCWR, while 8 SNPs were different (Additional file 5). The alignment of amino acid sequence showed that, there were 7 amino acid variations between SR61 and YJCWR, and the other 19 amino acids were identical (Additional file 6). Besides, Tan et al (2008) found that *PROG1* induced less number of primary branches (NPB), secondary branches (NSB) and grain number per panicle (GNP) on main panicle, as well as less grain yield per plant (GYP). It also was found that, comparing with that of HJX74, *TA7-RUF* in SR61significantly shortened the plant height, reduced number of secondary branches, lessened number of grains per panicle, and lightened thousand-grain weight (Additional file 7). Thus, *TA7-RUF* in the present study was inferred as the positive allele of *PROG1.* Additionally, another larger tiller angle gene, *TA8-RUF*, which mapped to the region of RM515-RM195 (20,286,300–21,478,312 bp) on chromosome 8, covered the position of the known gene *TIG1* (20,931,298–20,932,089 bp) for the larger tiller angle cloned from *O. rufipogon* (Zhang et al. 2019). The sequence alignment of *TIG1* in HJX74, SR76, and *O. rufipogon*, showed that SR76 and *O. rufipogon* had the same sequence in allele of *TIG1*, however, there were 3 SNPs variations in CDS region of HJX74 with *tig1* allele, causing 3 amino acid substitution (Fig. 6h, Additional files 8, 9). Moreover, 3 key SNPs in the promoter region, that is, -648 A to G, -449 C to T, and -310 C to T in HJX74 (Additional file 8), which were the identical variations of the promoter sequence in *TIG1* in indica cultivars (*tig1* allele) (Zhang et al. 2019). It resulted in decreased expression of *TIG1* in the adaxial side of tiller base and reduced tiller angle in comparison with *O. rufipogon*. Thus, *TA8-RUF* was considered as the positive allele of *TIG1.* Notably, *TA11-NIV* at the interval of RM26145 and PSM175 on chromosome 11 was inferred as a novel gene for larger tiller angle due to its different position from the reported tiller angle genes/QTLs on chromosome 11 (Abe et al. 2007; Li et al. 2007; Yu et al. 2005).

### Spreading Panicle

**Fig. 7:**
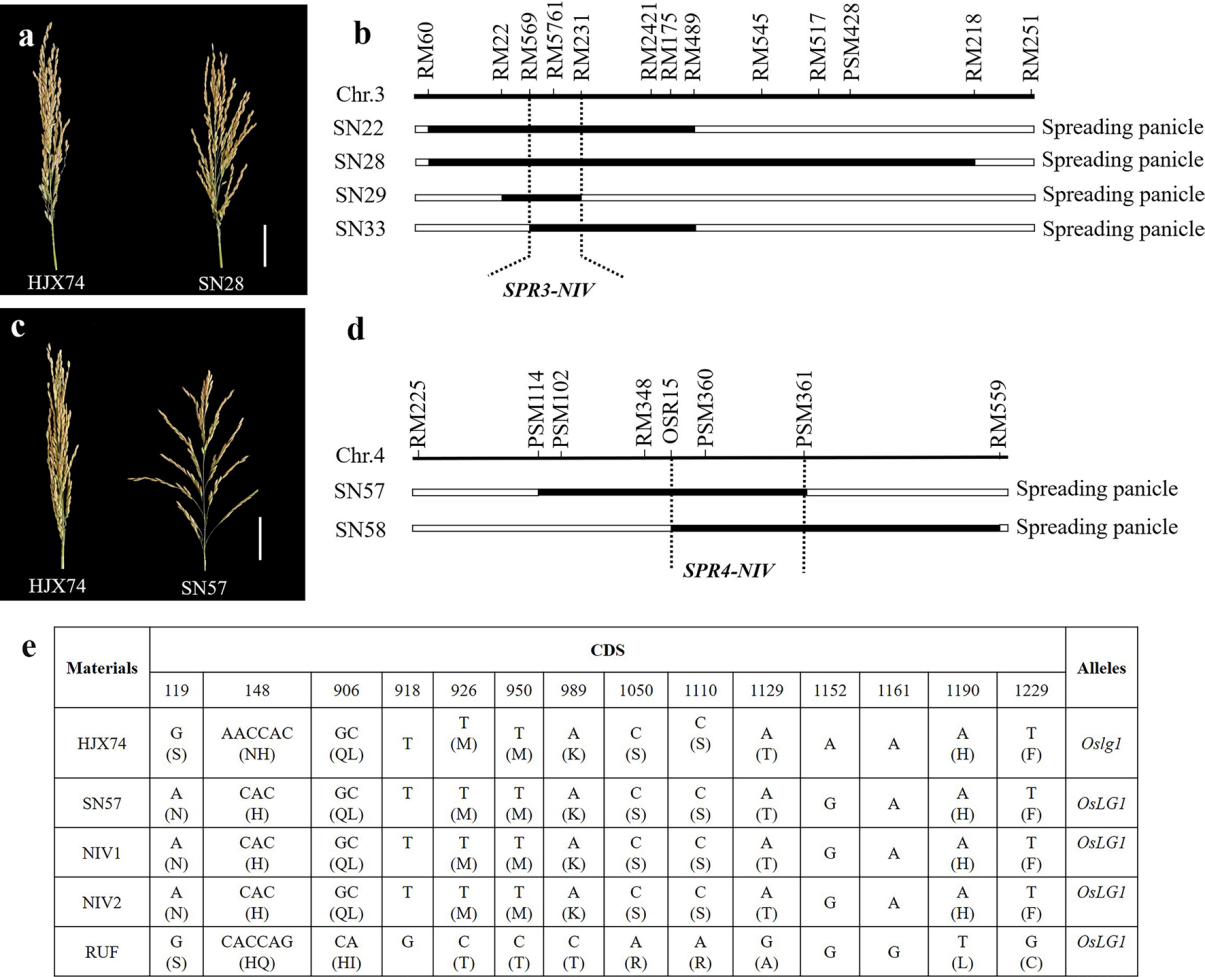
Phenotype of spreading panicles in SSSLs and the substitution mapping of the related genes. **a** and **c** show the panicle types of HJX74 and SSSLs, bars = 5.0 cm; **b** and **d** show the substitution mapping of the spreading panicle genes, *SPR3-NIV* and *SPR4-NIV*, respectively; **e** shows the CDS variations of *OsLG1*

Panicle architecture is another domestication trait in *Oryza* genus and also directly affects rice productivity. It is known that the wild rice species usually show spreading panicle, while *O. sativa* generally have compact panicles. Here, in NIV-SSSLs, 2 genes (*SPR3-NIV* and *SPR4-NIV*) for spreading panicles were identified. That is, *SPR3-NIV* was identified from 4 NIV-SSSLs (SN22, SN28, SN29, and SN33) with spreading panicles and carried the overlapped substituted segments on chromosome 3, *SPR3-NIV* was then delimited to a 0.54 Mb common region between markers RM569 and RM231(Fig. 7a, b). And *SPR4-NIV* was identified from both SN57 and SN58 with spreading panicles, and *SPR4-NIV* was delimited to the 0.83 Mb between markers OSR15 and PSM361 at the overlapped substituted segments on chromosome 4 (Fig. 7c, d). A dominant gene *OsLG1* was cloned from *O. rufipogon* previously, which controlling the spreading panicles (Zhu et al. 2013). Here, we found that both *SPR4-NIV* and *OsLG1* located behind the marker RM348 on the long arm of chromosome 4, and the physical interval of *SPR4-NIV* (33,031,813–33,865,656 bp) covered that of *OsLG1* (33,488,512–33,492,876 bp). Then, the CDS alignment of *OsLG1* in HJX74, SN57, and *O. nivara*, showed that SN57 and *O. nivara* had the same sequence, but different from HJX74. That is, 119th A to G induced an amino acid substitution of asparagine to serine, however, 1152th G to A is a synonymous mutation. Besides, a 3-bp insertion “AAC” in the site of 148th in HJX74 induced an amino acid asparagine insertion (Fig. 7e, Additional files 10 and 11). Furthermore, we sequenced the key SNP in the promoter region of *OsLG1*, which reported to be the key mutation (Zhu et al. 2013). As a result, the key SNP of A to G appears the same in the promoter region (Additional file 12). Thus, we inferred that *SPR4-NIV* in SN57 and SN58 from *O. nivara* in the present study might be a positive allele of *OsLG1* in *O. rufipogon* for the spreading panicle in wild rice species. Additionally, another gene, *SPR3-NIV*, identified in the present study was predicted as a novel gene from *O. nivara*, due to no reported gene on chromosome 3 till now for spreading panicle in rice. Without doubt, it was worth further mining the causal gene of *SPR3-NIV* to assess the functions and molecular mechanisms of the transition from spreading panicle to the erect during domestication.

### Awn

**Fig. 8:**
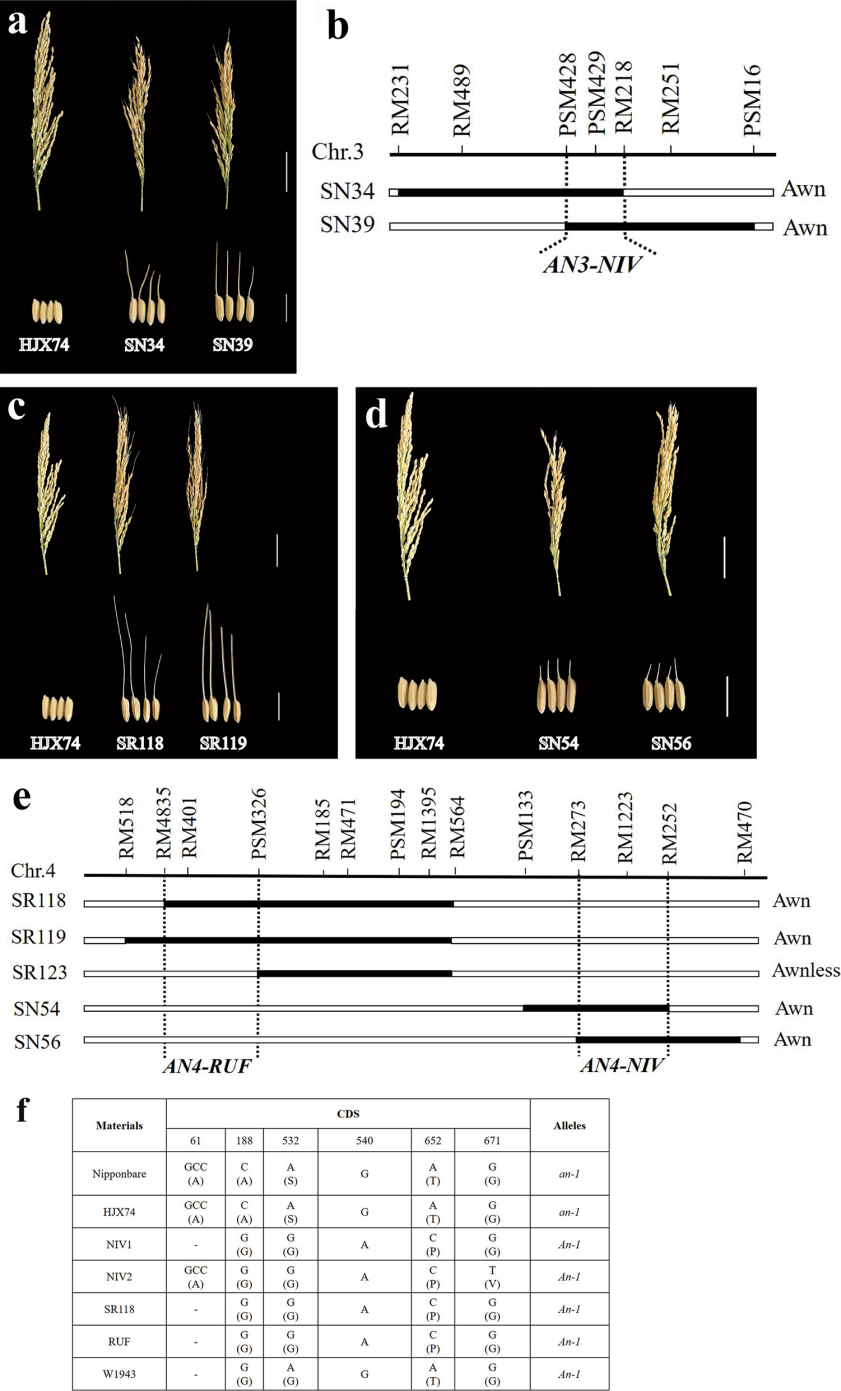
Phenotype and the substitution mapping of the genes for awns in SSSLs. **a**, **c**, and **d** show the panicles and seeds of SSSLs (with awns) and HJX74 (awnless); **b **and **e** show the substitution mapping of the awn genes in SSSLs. **f** shows the CDS variations of *An-1*. In **a**, **c**, and **d**, upper bars = 5.0 cm, lower bars = 1.0 cm

It was found that seeds of 6 SSSLs possessed awns, while seeds of HJX74 showed awnless. Seeds of SN34 and SN39 had long awns, and they showed overlapped substituted segment on chromosome 3, the gene of *AN3-NIV* for long awn was then assigned and delimited to the 1.55 Mb common interval between PSM428 and RM218 in SN34 and SN39 (Fig. 8a, b). Besides, both SN54 and SN56 showed short awns at the tip of the seeds, and the gene *AN4-NIV* for short awns was narrowed down to a 1.32 Mb common region of the overlapped substituted segments between RM273 and RM252 on chromosome 4 in SN54 and SN56 (Fig. 8d, e). Additionally, in RUF-SSSLs, seeds of SR118 and SR119 presented long awns while SR123 was awnless. Through substitution mapping, *AN4-RUF* for long awn was delimited to the interval of RM4835 and PSM326 on chromosome 4, which was the region overlapped in SR118 and SR119, but did not overlap with SR123 (Fig. 8c, e). Comparatively, the chromosome location of *AN4-NIV* (Chr. 4: 24044220–25364277) was very close to *LABA1* (*An-2*) (Chr. 4: 25959399–25963504), a cloned gene controlling long and barbed awns in *O. rufipogon* (Gu et al. 2015; Hua et al. 2015). A 1-bp frame-shift deletion of cytosine at + 69 bp in the 1st exon of *LABA1* induced awnless phenotype in an *indica* cultivar 9311 (Hua et al. 2015), but no variations occurred in the genome sequences of *LABA1* in HJX74 and SN54 in the present study (Additional file 13), thus we guessed that *AN4-NIV* would be a novel gene different from *LABA1*. Additionally, it was found that a reported gene *An-1* for awn in rice (Chr. 4: 16731738–16735336) was in the region of *AN4-RUF* (Chr. 4: 7971563–16789753) (Luo et al. 2013b). By comparing the sequence of *An-1* (Fig. 8f), we found the same key mutation, 188^th^ SNP from C to G, contributes to the awn as previous study (Luo et al. 2013b). The alignments of the genomic DNA sequence and amino acid sequence of *An-1* (Additional files 14 and 15) indicate *AN4-RUF* may be the positive allele of *An-1*. Importantly, *AN3-NIV* detected in the present study did not share any common region with the reported awn genes in rice, it would be considered as novel genes detected in NIV-SSSLs.

### Seed Shattering

**Fig. 9:**
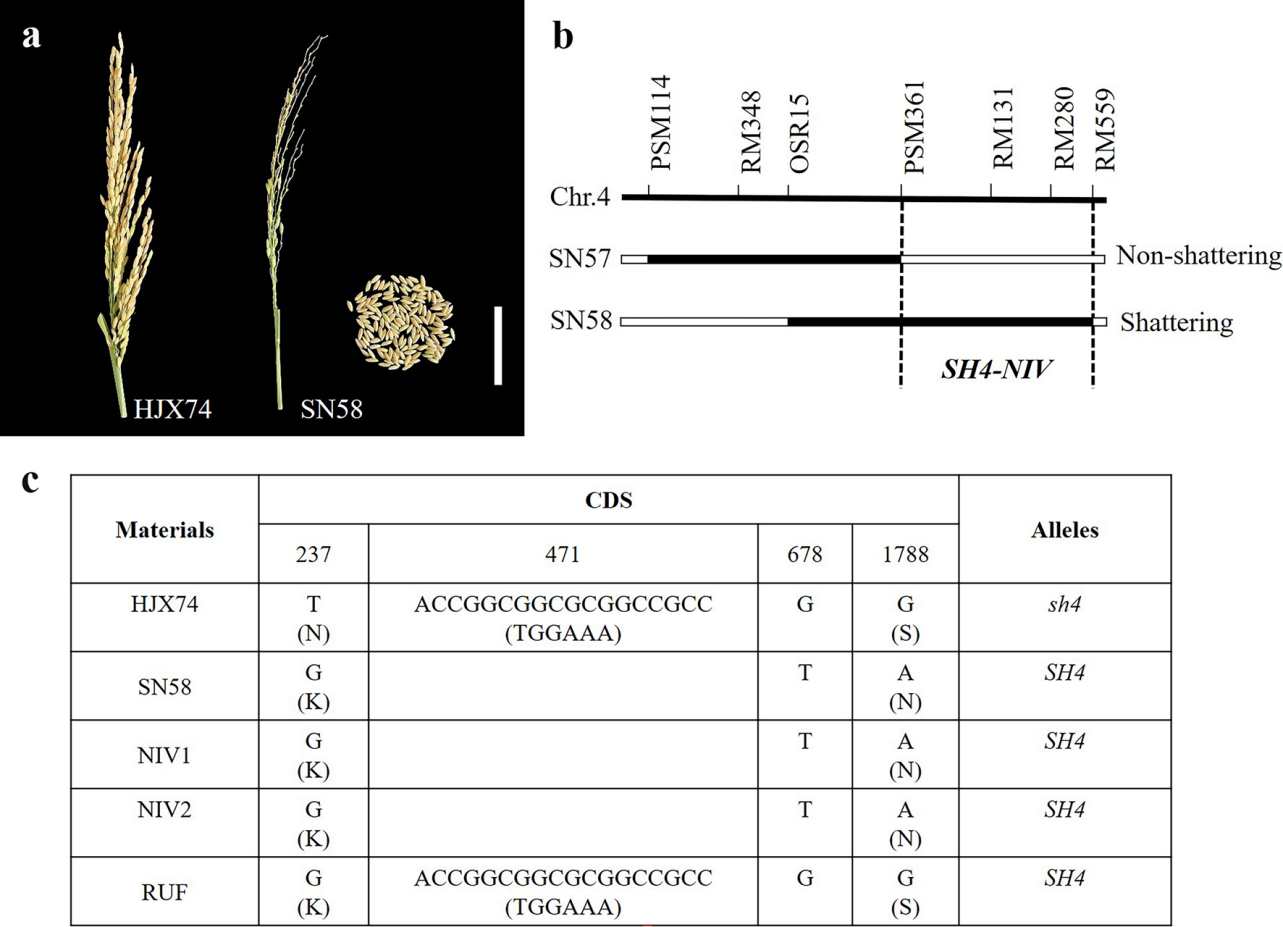
Seed shattering phenotype and the substitution mapping of *SH4-NIV*. **a** shows the shattered seeds and panicle of SN58, and panicles with non-shattered seeds in HJX74. **b** shows the substitution mapping of *SH4-NIV* for seed shattering. **c** shows the CDS variations of *SH4*. bar = 10 cm

Among the SSSLs, SN58 from NIV-SSSLs showed serious seed shattering like *O. nivara*, while HJX74 showed non-shattering (Fig. 9a). The substituted segment of SN58 was between markers OSR15 and RM559 on chromosome 4 and overlapped with that of SN57. However, SN57 showed non-shattering. Then *SH4-NIV* of the seed shattering gene was assigned and delimited to the 1.47 Mb between markers PSM361 and RM559 in SN58 through substitution mapping (Fig. 9b). In the previous study, *SH4* has been cloned from *O. nivara*, which coding region is physically located in 34,012,126- 34,014,305 bp of rice chromosome 4. At present, the identified gene, *SH4-NIV* for seed-shattering in SN58 was in the physical interval of 33,865,656–35,336,720 bp, which covered the region of *SH4.* Through sequencing and alignment of the CDS region of *SH4* in HJX74, SN58, and *O. nivara*, it showed that SN58 and *O. nivara* had the same sequences, but that different from HJX74. Compared with SN58, in the exon 1 of HJX74, base substitutions occurred at 237th (glycine to threonine), 678th (threonine to glycine) sites, and 1788th (alanine to glycine), respectively. The first and third base substitutions induced amino acid variations from lysine to asparagine, and asparagine to serine, respectively. However, the 678th substitution was a synonymous mutation. Additionally, an 18-bp deletion occurred within 471-489th position in both SN58 and *O. nivara*, but not in HJX74, which induced shortened protein molecules with 6 amino acid deletions (Fig. 9c). Nevertheless, the deletion did not occurred in *SH4* allele in the report of Li et al (2006). Importantly, it is reported that the 237th nucleotide substitution of glycine to threonine in exon 1 of *SH4* induced an amino acid substitution from lysine to asparagine in *O. sativa*, which was the key mutation or selection site for the development of non-shattering cultivars during rice domestication (Zhu et al. 2012). Therefore, *SH4-NIV* in SN58 was considered the novel allele of *SH4* for seed shattering. However, whether or not the 18-bp deletion in *SH4-NIV* participating in the regulation of seed shattering remains a question and needs to be uncovered in the future.

### Red Pericarp

**Fig. 10:**
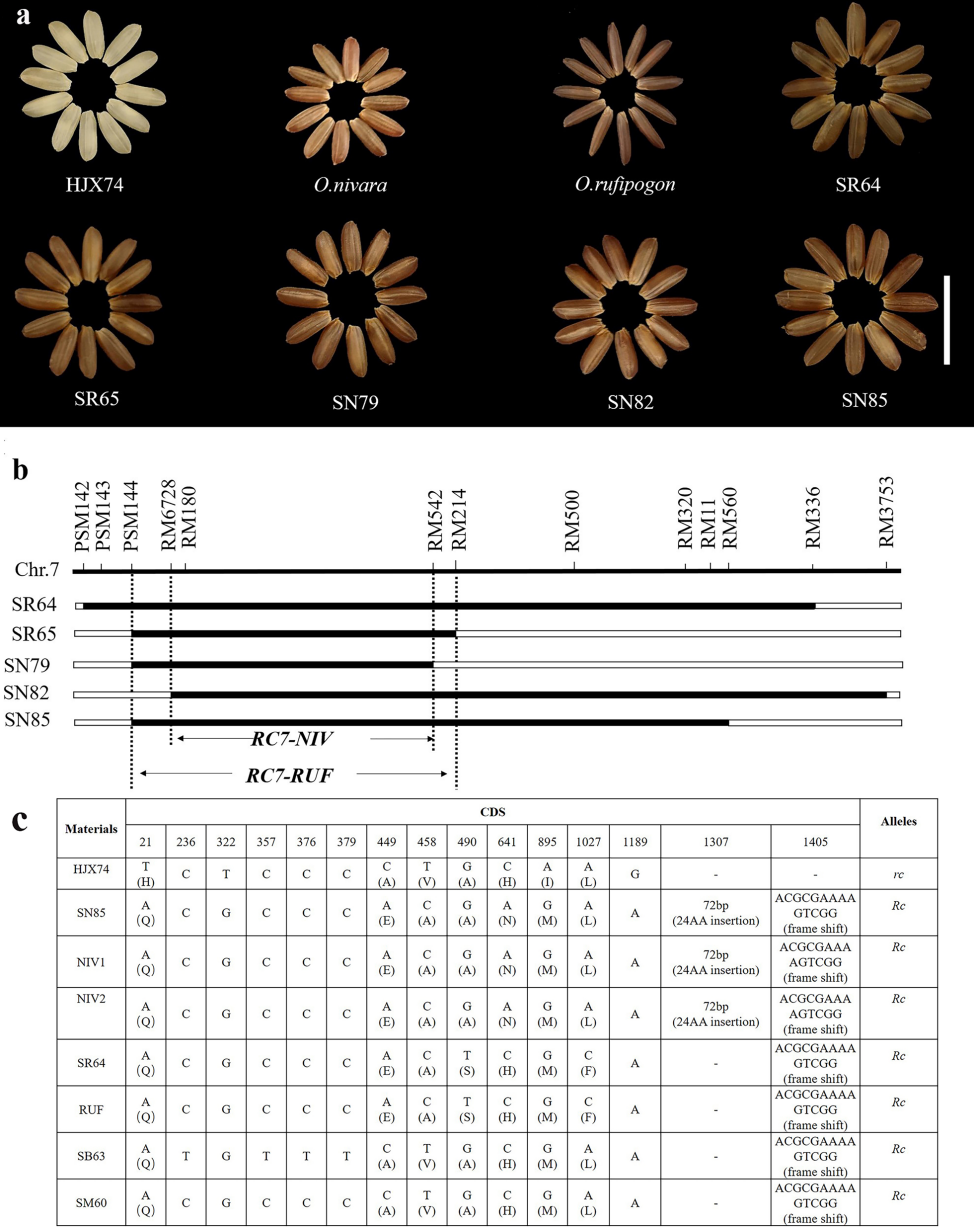
Pericarp phenotypes and the substitution mapping of the related genes. **a** Phenotype of the red pericarp in donors and SSSLs, and the white pericarp in HJX74, bar = 1.0 cm. **b** Substitution mapping of the red pericarp genes in SSSLs. **c** CDS sequence variations of *Rc* gene in SSSLs from different donors and HJX74. Herein, SB63 is a SSSL from the donor of *O. barthii* (Zhao et al. 2019), SM60 is a SSSL from the donor of *O. meridionalis* (He et al. 2017), both of them have the same background of HJX74

In the present study, the wild rice donors, *O. rufipogon* and *O. nivara*, have red pericarps, while HJX74 has the white pericarp. In SSSLs populations, 3 NIV-SSSLs (SN79, SN82, and SN85) and 2 RUF-SSSLs (SR64 and SR65) have red pericarps (Fig. 10a). The substituted segments in these 5 SSSLs were all on chromosome 7, thus the related genes, *RC7-RUF* from *O. rufipogon* and *RC7-NIV* from *O. nivara*, were detected and delimited in these SSSLs. *RC7-RUF* was delimited to an 8.13 Mb overlapped interval of PSM144 to RM214 in SR64 and SR65, and *RC7-NIV* was narrowed down to the 6.98 Mb overlapped interval of RM6728-RM542 among SN79, SN82, and SN85 (Fig. 10b). Moreover, *RC7-RUF* and *RC7-NIV* shared a common interval of RM6728-RM542. In our previous study, red pericarps were observed in brown rice of several SSSLs, that is SM60 from *O. meridionalis* and SB62 and SB63 from *O. barthii*, and they all carried the substituted segments on chromosome 7 (He et al. 2017). It is known that the cloned rice red pericarp gene *Rc* was also located on chromosome 7 (Sweeney et al. 2006). Furthermore, the sequence alignment of the gene *Rc* was performed in SN85, SR65, SM60, SB63, and HJX74. Totally, we found 13 SNPs and 2 Indels variations in *Rc* which induced 7 amino acid substitutions. Besides, a 14-bp sequence showing as “ACGCGAAAAGTCGG” from 1405th site in exon 6 in *Rc* was presented in the 4 SSSLs, but it was deleted in HJX74 (Fig. 10c). In addition, there was a 72-bp insertion at the site of 1307th in the CDS of *Rc* in SN85, which caused a 24 amino acid insertion. The previous results demonstrated that the 14-bp-deletion is the key variation inducing frame-shifting in *Rc* associated with the change of red to white pericarp (Sweeney et al. 2006). Thus, it was inferred that all above SSSLs of 4 wild rice species in our study carried the positive allele of *Rc* for red pericarp, and that in *O. nivara* showed different with other wild rice in the 72-bp insertion, but it did not affect the phenotype of red pericarp.

## Discussion

Discovery and innovation of genetic resources is the first key step for plant breeding. It is well recognized that wild relatives are the important origins of natural genes for crop breeding. In modern rice breeding, wild rice species, particularly those belonging to the AA genome group, have proven to be precious gene pools for cultivar improvement (McCouch et al. 2007). Much more breeding practices have proven that chromosome segment substitution lines (CSSLs) are the elite germplasms for breeding by design, due to its superiority in gene discovery and utilization (Ali et al. 2010; Balakrishnan et al. 2019). Previously, we have developed 3 sets of SSSLs from 3 AA-genome wild rice species, including *O. meridionalis* (He et al. 2017), *O. barthii*, and *O. glumaepatula* (Zhao et al. 2019), in the genetic background of the elite indica rice variety, HJX74. These SSSLs have showed obvious effects on the gene mining from wild rice species (Fang et al. 2019; Tan et al. 2020; Wang et al. 2020; Zou et al. 2020). Currently, we developed 2 sets of SSSLs from *O. rufipogon* and *O. nivara*. The substituted segments distributed on 12 chromosomes with the total lengths of 1565.77 Mb and the coverage lengths of 548.05 Mb. Compared with the previous reported CSSLs or ILs of *O. rufipogon* (Ding et al. 2022; Furuta et al. 2016; Ma et al. 2019; Qiao et al. 2016; Xiao et al. 1998; Xie et al. 2006; Zhang et al. 2014) and *O. nivara* (Furuta et al. 2016; Liu et al. 2021; Ma et al. 2016; Surapaneni et al. 2017; Zhang et al. 2022). Each SSSL in the present study carried the only one different substituted segment in the unique background of HJX74. These SSSLs laid the important foundation for both expansion of the SSSLs library of wild rice species in our laboratory, but also provide invaluable germplasm resources for gene mining, allelic variation analysis, and permanent ex-situ conservation of the genes from wild rice species.

Based on the SSSLs from *O. rufipogon* and *O. nivara*, 38 SSSLs were found to show obvious domestication traits, including tiller angle, spreading panicle, awn, seed shattering, and red pericarp. And 11 related genes were identified and mapped on 5 chromosomes. Furthermore, through comparing the genomic region and/or sequence of the genes, *TA7-RUF* was inferred as the positive allele of *PROG1* from *O. rufipogon* (Jin et al. 2008; Tan et al. 2008). Similarly, *TA8-RUF* was predicted as the positive allele of *TIG1* from *O. rufipogon* (Zhang et al. 2019). Besides, *SPR4-NIV* carried by SN57 and SN58 from *O. nivara* in the present study was considered the positive allele of *OsLG1* in *O. rufipogon* (Ishii et al. 2013; Zhu et al. 2013). Additionally, through sequence alignment, *SH4-NIV* for seed-shattering in SN58 was considered the positive allele of *SH4* cloned by Li et al (2006) in *O. nivara*. In *SH4*, the 237th nucleotide substitution of glycine for threonine in exon 1 was proven the key mutation or selection site for the non-shattering during rice domestication. It is known that red pericarp is one of the basic characters of wild rice species, which is controlled by two complementary genes, *Rc* and *Rd*, in rice*.* Previously, *Rc* has been mapped on chromosome 7 and *Rd* mapped on chromosome 1 (Furukawa et al. 2007; Sweeney et al. 2006). The combination of the two pairs of dominant genes *Rc* and *Rd* produce red pericarp in wild rice species, including *O. rufipogon* and *O. nivara*, while the cultivated rice varieties with white pericarps are mostly resulted from a 14-bp frame-shift deletion in the 6th exon of the *Rc* gene (Sweeney et al. 2006). In the present study, we found that the SSSLs of SN79, SN82, and SN85 from *O. nivara*, and SR64 and SR65 from *O. rufipogon* showed the red pericarps. Meanwhile, in our previous study, SM60 from *O. meridionalis* and SB62 and SB63 from *O. barthii* also showed the red pericarps. These 8 SSSLs carried the related gene *Rc* on the substituted segment of chromosome 7*,* and the 14-bp sequence in 1405–1418th site in exon 6 of the dominant *Rc* in the SSSLs with red pericarp was in the same sequence, but the deletion of the 14-bp sequence was found in the recessive *rc* gene in HJX74 with white pericarp. It has been demonstrated that most white pericarp in rice varieties were resulted from the 14-bp deletion in *Rc* gene (Sweeney et al. 2006)*.* Recently, Zhu et al. (2019) developed a CRISPR/Cas9-mediated method to functionally restore the recessive *rc* allele through reverting the 14-bp frame-shift deletion to in-frame mutation, and they successfully converted 3 elite white pericarp rice varieties into red ones. On the whole, the evolutionary process of rice domestication is filled with mysteries yet to be fully uncovered. Identification of the novel alleles or genes associated with the domestication will play key roles not only for understanding the cultivated rice origin and variation but also for those processes in other crops. Base on the present results, 4 novel domestication genes are worth further study in gene cloning and functional analysis to better reveal the molecular mechanisms in regulating the domestication traits. On the other hand, along with the development of CRISPR/Cas9 technology, gene editing is becoming very important in creating elite germplasms for breeding. It has showed successes in improving yield-related traits and stress resistance by editing the relative genes from wild species, such as in ground cherry (*Physalis pruinosa*) (Lemmon et al. 2018) and wild tomato (Li et al. 2018; Zsogon et al. 2018). Therefore, it provides the support proofs from another perspective that the SSSLs population and the natural genes’ mining are important in the germplasm innovation through gene mining and editing.

## Conclusions

In conclusion, we constructed 172 NIV-SSSLs (39 NIV1-SSSLs and 133 NIV2-SSSLs) and 123 RUF-SSSLs in an *indica* rice HJX74 background. The three sets of SSSLs population covered approximately 26.56%, 61.37% and 75.49% of the wild rice genome and the average length of substitute segment was 4.36 Mb, 4.80 Mb and 6.19 Mb in *NIV1*, *NIV2*, and *RUF*, respectively. In addition, we identified 11 domestication-related genes and 4 of them are novel genes. The three sets of SSSLs described herein could provide a powerful tool for detecting favorable genes from wild rice and creating germplasm resources for rice breeding.

## Methods

### Materials and Cultivation

In the development of the single segment substitution lines (SSSLs), two AA-genome wild rice species were used as donors, that is, *O. rufipogon* (*RUF*, with the IRGC accession number of IRGC106149, indigenous to Laos) and *O. nivara* (*NIV1*, with the IRGC accession number of IRGC104309; *NIV2*, with the IRGC accession number of IRGC105919. Both of them are indigenous to Thailand). Seeds of the wild rice species were kindly provided by the International Rice Research Institute (IRRI). And HJX74, an elite indica cultivar bred by our laboratory, was used as a recipient. All the materials, including HJX74, SSSLs, and the intermediate materials in different backcross generations between parents, were grown in the experimental paddy field of South China Agricultural University (SCAU) in Guangzhou (23°07′ N, 113°15′ E). Plants of *O. rufipogon* and *O. nivara* were potted at the wild rice core collection nursery of SCAU. All the SSSLs for gene identification and mapping were grown in a two-row plot with 10 individuals per row as a replication, each with three replications. The conventional paddy field management in South China was implemented.

### Development Process of the SSSLs

The development procedure of the SSSLs was similar to that of the SSSLs of other wild rice species in our previous reports (He et al. 2017; Zhao et al. 2019). Briefly, as shown in Fig. 2, SSSLs were developed through the crosses between HJX74 and donors, *RUF* (Fig. 1a) and two accessions of *O. nivara*, *NIV1* and *NIV2* (Fig. 1b, c), the successive backcrosses and self-crosses in the later generations were conducted by using HJX74 (Fig. 1d) as the recipient, combined with the marker detections. The substituted segments of the individual plants in each family line were detected by using the polymorphic SSR markers from BC_1_F_1_ to the advanced backcross filials and the self-cross generations, and SSSLs were mainly selected during BC_3_F_2_ to BC_5_F_4_ generations (Fig. 2). During the process of developing SSSLs, from about more than 600 SSR markers in rice genome (data not shown), 265, 269, and 284 polymorphic markers between HJX74 and *NIV1*, HJX74 and *NIV2*, and HJX74 and *RUF* were selected, respectively, which distributed evenly on 12 chromosomes (Additional files 1, 2). The polymorphic markers were used for the detections of the target substituted segments in the foreground, which were also used in the detection of the residual segments in the background of the candidate SSSLs. At last, each candidate plant carried only one homozygous substituted segment in the background of HJX74 was selected as a SSSL. Then each selected SSSL was planted as a family line for confirmation of the substituted segment and the genetic constitution analysis of the SSSL population. According to the same methods in the previous report (He et al. 2017), the genome constitution of the substituted segments were surveyed in each donor’ SSSL population, including the total length of the substituted segments, mean length of each substituted segment, coverage length of the substituted segments, and coverage percent of the rice genome. The genotypic graphics of the substituted segments were charted using Map chart2.2. In addition, the marker detection process of each plant, including DNA extraction, PCR amplification, and electrophoresis analysis, were performed according to our previous methods (He et al. 2017).

### Trait investigation

Domestication traits were investigated in the SSSLs in the early cropping season and the late cropping season in 2016–2017, and it was verified in the late cropping season in 2019. In brief, each SSSL was planted in two rows with 10 plants in each row. Tiller angle was investigated at the vigorous tillering stage using a protractor, and the angle between the two outermost tillers was recorded for each plant in the SSSLs populations(Yu et al. 2005; Zhang et al. 2013), and the mean values were obtained from 10 plants in center of the rows for each material. At mature stage, seed shattering, awn, and spreading panicle were observed and recorded in the field. After seeds were harvested and dried, the pericarp colors of the dehusked seeds were observed in the lab.

### Detection and Substitution Mapping of the Domestication Genes

In the SSSLs populations, we found a number of SSSLs showed domestication traits. Then, by comparing the substituted segments’ marker intervals of the SSSLs, the related genes were identified and delimited to a certain interval through substitution mapping. Moreover, in order to determine if the SSSLs carried a novel gene or allele for the domestication traits with the known genes, we searched the known genes through the research literatures and the databases in rice (http://rice.uga.edu/), and compared the physical positions of the linked markers with the identified genes in the present study. If both genes fell in the same interval or with overlapped interval, the sequence alignments were conducted to analyze the allelism.

### PCR and Sequence Alignment of the Domestication Genes

To further understand the allelic variations or allelism of the domestication genes, we performed the PCR and sequence alignment of the known genes (*PROG1*, *TIG1*, *OsLG1*, *LABA1*, *SH4*, *Rc*), which falling in the intervals of the identified genes in the present study, among the SSSLs, donors and HJX74. The genomic DNA was extracted from young leaves of plants by CTAB method (Murray et al. 1980). The specific primers for gene amplification and sequencing (Additional file 16) were designed using the PRIMER PREMIER 5 program based on the reference genomic sequence of Nipponbare (IRGSP1.0), and KOD-Plus-Neo polymerase (TOYOBO, Japan) was used for PCR. PCR reactions were carried out in a 50 μL reaction final volume (X μL ~ 200 ng template DNA, 5 μL 1 × PCR buffer, 5 μL 0.2 mM dNTPs, 3 μL 1.5 mM MgSO_4_, 1.5 μL 0.3 μM forward primer, 1.5 μL 0.3 μM reverse primer, 1 μL 1 U KOD-Plus-Neo polymerase, and 33-X μL ddH_2_O). The program for PCR amplification was as follows: pre-denature 94 °C, 2 min, 35 cycles (denature for 98 °C 10 s, annealing for (Tm) °C 30 s, extension for 68 °C 30 s/kb), and then a final extension at 68 °C for 5 min. Here, Tm is the temperature of melting, which depends on the primers, and the extension time depends on the length of genes. The PCR products were detected on 1% agarose gels in TBE buffer and then visualized by gel imaging analysis system. The sanger sequencing of PCR products were conducted by Tsingke Biotech Co. Ltd (Beijing, China), and assembled using SeqMan (in the DNASTAR. Lasergene package). The alignment of DNA sequence and amino acid sequence were performed using BioXM 2.7.1 software (http://cbi.njau.edu.cn/BioXM/).

### Supplementary Information

**Additional file 1:** Chromosome distribution of the polymorphic SSR markers used for SSSLs development.

**Additional file 2:** Information of the polymorphic SSR markers used in the SSSLs development.

**Additional file 3: **The specific information of 295 SSSLs.

**Additional file 4:** The information of the chromosome distribution of the substituted segments in the three sets of SSSLs.

**Additional file 5: **Coding sequence alignment of *PROG1*.

**Additional file 6:** Amino acid sequence alignment of *PROG1*.

**Additional file 7:** Comparison of the main agronomic traits in SR61 and HJX74.

**Additional file 8:** Promoter region and genome DNA sequence alignment of *TIG1*.

**Additional file 9: **Amino acid sequence alignment of *TIG1.*

**Additional file10:** CDS alignment of *OsLG1*.

**Additional file 11:** Amino acid sequence alignment of *OsLG1*.

**Additional file 12: **Alignment of key promoter sequence of *OsLG1*.

**Additional file 13:** Genome sequence alignment of *LABA1*.

**Additional file 14:** Genome sequence alignment of *An-1*.

**Additional file 15:** Amino acid sequence alignment of *An-1*.

**Additional file 16:** Primers for gene amplification.

## Data Availability

All data generated or analyzed during this study are included in this published article and its additional information files.

## Abbreviations

CDS: Coding sequence
GNP: Grain number per panicle
GYP: Grain yield per plant
NPB: Number of primary branches
NSB: Number of secondary branches
QTL: Quantitative trait locus
SSSL: Single segment substitution line

## Data analysis and illustration

Data were processed primarily by using the Microsoft Excel 2016. According to the same methods in the previous report (He et al. 2017), the genome constitution of the substituted segments were surveyed in each SSSL population, including the total length of the substituted segments, mean length of each substituted segment, coverage length of the substituted segments, and coverage percent of the rice genome. The genotypic graphics of the substituted segments were charted using Map chart2.2 (Voorrips 2002).
