# Supplementary material for: Substitution Mapping and Allelic Variations of the Domestication Genes from O. rufipogon and O. nivara
Source: Rice (N Y). 2023 Sep 5;16:38. doi: 10.1186/s12284-023-00655-y (PMC10480103; doi:10.1186/s12284-023-00655-y)
Supplement: Supplementary file 1 — Additional file 1: Chromosome distribution of the polymorphic SSR markers used for SSSLs development. [file 12284_2023_655_MOESM1_ESM.docx]

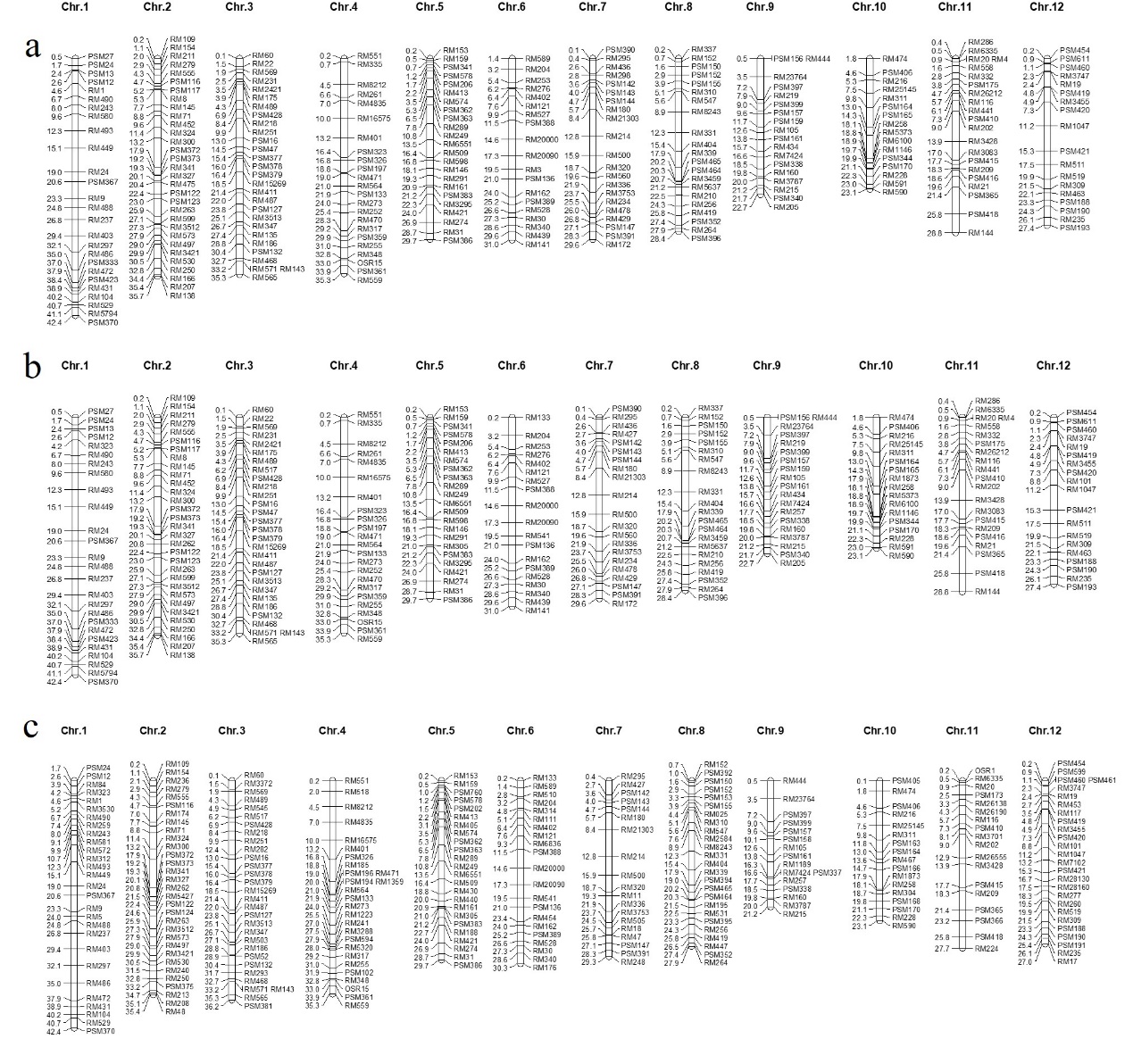


**Additional file 1. Chromosome distribution of the polymorphic SSR markers between recipient and donors for SSSLs development.**

a, b and c show polymorphic SSR markers for development of NIV1-SSSLs, NIV2-SSSLs and RUF-SSSLs, respectively.
