# Supplementary material for: Substitution Mapping and Allelic Variations of the Domestication Genes from O. rufipogon and O. nivara
Source: Rice (N Y). 2023 Sep 5;16:38. doi: 10.1186/s12284-023-00655-y (PMC10480103; doi:10.1186/s12284-023-00655-y)
Supplement: Supplementary file 2 — Additional file 2: Information of the polymorphic SSR markers used in the SSSLs development. [file 12284_2023_655_MOESM2_ESM.docx]

**Additional file 2** Information of the polymorphic SSR markers used in the SSSLs development

| Chr. | Numbers of SSR markers | | | |  | Density of SSR markers (No. per Mb) | | | |
| --- | --- | --- | --- | --- | --- | --- | --- | --- | --- |
|  | NIV1-SSSLs | NIV2-SSSLs | RUF-SSSLs | Total markers |  | NIV1-SSSLs | NIV2-SSSLs | RUF-SSSLs | Total markers |
| 1 | 26 | 26 | 28 | 34 |  | 0.60 | 0.60 | 0.65 | 0.79 |
| 2 | 31 | 31 | 31 | 41 |  | 0.86 | 0.86 | 0.86 | 1.14 |
| 3 | 28 | 29 | 30 | 37 |  | 0.77 | 0.80 | 0.82 | 1.02 |
| 4 | 23 | 23 | 29 | 34 |  | 0.65 | 0.65 | 0.82 | 0.96 |
| 5 | 23 | 23 | 24 | 30 |  | 0.77 | 0.77 | 0.80 | 1.00 |
| 6 | 19 | 19 | 21 | 27 |  | 0.61 | 0.61 | 0.67 | 0.86 |
| 7 | 21 | 21 | 19 | 27 |  | 0.71 | 0.71 | 0.64 | 0.91 |
| 8 | 21 | 21 | 25 | 30 |  | 0.74 | 0.74 | 0.88 | 1.05 |
| 9 | 18 | 19 | 16 | 22 |  | 0.78 | 0.83 | 0.70 | 0.96 |
| 10 | 16 | 17 | 17 | 23 |  | 0.69 | 0.73 | 0.73 | 0.99 |
| 11 | 21 | 21 | 18 | 28 |  | 0.72 | 0.72 | 0.62 | 0.96 |
| 12 | 18 | 19 | 26 | 30 |  | 0.65 | 0.69 | 0.94 | 1.09 |
| Total | 265 | 269 | 284 | 363 |  | - |  | - | - |
| Mean | 22.08 | 22.42 | 23.67 | 31.92 |  | 0.71 | 0.73 | 0.76 | 0.98 |
