# Supplementary material for: Substitution Mapping and Allelic Variations of the Domestication Genes from O. rufipogon and O. nivara
Source: Rice (N Y). 2023 Sep 5;16:38. doi: 10.1186/s12284-023-00655-y (PMC10480103; doi:10.1186/s12284-023-00655-y)
Supplement: Supplementary file 4 — Additional file 4: The information of the chromosome distribution of the substituted segments in the three sets of SSSLs. [file 12284_2023_655_MOESM4_ESM.docx]

**Additional file 4. The information of the chromosome distribution of the substituted segments in the three sets of SSSLs**

| Chr. | Number of segments | | | |  | Segments length (Mb) | | | |  | Average length (Mb) | | | |  | Coverage length (Mb) | | | |  | Coverage rate（%） | | | |
| --- | --- | --- | --- | --- | --- | --- | --- | --- | --- | --- | --- | --- | --- | --- | --- | --- | --- | --- | --- | --- | --- | --- | --- | --- |
|  | NIV1 | NIV2 | RUF | Total |  | NIV1 | NIV2 | RUF | Total |  | NIV1 | NIV2 | RUF | Total |  | NIV1 | NIV2 | RUF | Total |  | NIV1 | NIV2 | RUF | Total |
| 1 | 0 | 7 | 13 | 20 |  | 0 | 32.31 | 99.26 | 131.57 |  | - | 4.62 | 7.64 | 6.58 |  | 0 | 15.81 | 36.53 | 52.34 |  | 0 | 36.54 | 84.42 | 120.95 |
| 2 | 7 | 6 | 16 | 29 |  | 24.94 | 11.53 | 88.95 | 125.42 |  | 3.56 | 1.92 | 5.56 | 3.47 |  | 10.47 | 10.20 | 28.89 | 49.56 |  | 29.14 | 28.37 | 80.39 | 137.90 |
| 3 | 6 | 22 | 15 | 43 |  | 24.86 | 117.93 | 70.35 | 213.14 |  | 4.97 | 5.13 | 4.69 | 4.26 |  | 20.92 | 24.35 | 30.79 | 76.06 |  | 57.45 | 66.88 | 84.56 | 208.89 |
| 4 | 5 | 6 | 7 | 18 |  | 18.26 | 33.05 | 47.17 | 98.49 |  | 3.04 | 6.61 | 6.74 | 4.82 |  | 11.05 | 20.58 | 26.39 | 58.02 |  | 31.12 | 57.98 | 74.34 | 163.44 |
| 5 | 3 | 10 | 7 | 20 |  | 3.69 | 58.51 | 16.38 | 78.58 |  | 1.23 | 5.85 | 2.34 | 3.74 |  | 3.03 | 22.00 | 10.99 | 36.02 |  | 10.11 | 73.43 | 36.68 | 120.22 |
| 6 | 0 | 4 | 7 | 11 |  | 0 | 20.88 | 23.24 | 44.12 |  | - | 5.22 | 3.32 | 4.01 |  | 0 | 20.88 | 8.84 | 29.72 |  | 0 | 66.81 | 28.28 | 95.10 |
| 7 | 3 | 16 | 9 | 28 |  | 11.70 | 102.41 | 71.01 | 185.12 |  | 3.90 | 6.40 | 7.89 | 6.19 |  | 7.62 | 25.39 | 21.72 | 54.74 |  | 25.67 | 85.51 | 73.12 | 184.31 |
| 8 | 1 | 3 | 18 | 22 |  | 5.01 | 9.31 | 157.38 | 171.70 |  | 5.01 | 3.10 | 8.74 | 7.57 |  | 5.01 | 6.55 | 25.36 | 36.92 |  | 17.61 | 23.01 | 89.17 | 129.79 |
| 9 | 6 | 16 | 5 | 27 |  | 17.95 | 67.37 | 36.1 | 121.42 |  | 2.99 | 4.21 | 7.22 | 3.83 |  | 11.38 | 19.11 | 21.19 | 51.68 |  | 49.46 | 83.03 | 92.08 | 224.57 |
| 10 | 4 | 5 | 10 | 19 |  | 33.93 | 11.59 | 61.05 | 106.57 |  | 8.48 | 2.32 | 6.1 | 3.82 |  | 12.97 | 6.97 | 23.02 | 42.95 |  | 55.88 | 30.02 | 99.18 | 185.07 |
| 11 | 2 | 23 | 4 | 29 |  | 16.72 | 98.72 | 29.83 | 145.27 |  | 8.36 | 4.29 | 7.46 | 4.43 |  | 5.72 | 27.05 | 20.56 | 53.33 |  | 19.71 | 93.21 | 70.86 | 183.77 |
| 12 | 2 | 15 | 12 | 29 |  | 8.98 | 74.44 | 60.96 | 144.38 |  | 4.49 | 4.96 | 5.08 | 4.67 |  | 6.21 | 25.23 | 25.55 | 56.99 |  | 22.55 | 91.63 | 92.81 | 206.99 |
| Total | 39 | 133 | 123 | 295 |  | 166.05 | 638.04 | 761.68 | 1565.77 |  | 4.26 | 4.80 | 6.19 | 5.31 |  | 94.38 | 224.11 | 279.83 | 598.32 |  | 26.56 | 61.37 | 75.49 | 163.42 |

Note：NIV1, NIV2 and RUF represent the SSSLs of *O.* *nivara* (IRGC104309), *O.* *nivara* (IRGC105919) and *O. rufipogon* (IRGC106149), respectively.
