# Supplementary material for: Substitution Mapping and Allelic Variations of the Domestication Genes from O. rufipogon and O. nivara
Source: Rice (N Y). 2023 Sep 5;16:38. doi: 10.1186/s12284-023-00655-y (PMC10480103; doi:10.1186/s12284-023-00655-y)
Supplement: Supplementary file 5 — Additional file 5: Coding sequence alignment of PROG1. [file 12284_2023_655_MOESM5_ESM.rtf]

Teqing   ATGGATCCCTCATCGGCTTCTTGGCCGGCTCCGGCTTCTCCGCCGGTGGAGCTGTCCCTGTCCCTGCCGG  70
HJX74    ATGGATCCCTCATCGGCTTCTTGGCCGGCTCCGGCTTCTCCGCCGGTGGAGCTGTCCCTGTCCCTGCCGG  70
NIV1     ATGGATCCCTCATCGGCTTCTTGGCCGGCTCCGACTCCTCCGCCGGTGGAGCTGTCCCTGTCCCTGCCGG  70
NIV2     ATGGATCCCTCATCGGCTTCTTGGCCGGCTCCGACTCCTCCGCCGGTGGAGCTGTCCCTGTCCCTGCCGG  70
SR61     ATGGATCCCTCATCGGGCTTTTGGCCGGCTCCGACTCCTCCGCCGGTGGAGCTGTCCCTGTCCCTGCCGG  70
RUF      ATGGATCCCTCATCGGGCTTTTGGCCGGCTCCGACTCCTCCGCCGGTGGAGCTGTCCCTGTCCCTGCCGG  70
YJCWR    ATGGATCCCTCATCGGCTTCTTGGCCGGCTCCGACTCCTCCGCCGGTGGAGCTGTCCCTGTCCCTGCCGG  70
 
Teqing   CGGCGGCGGCGAGGAACCGCGACGAGGCGGCGCCGACGGCGATCGTCGACGGCAAGCAAGTGAGGCTGTT  140
HJX74    CGGCGGCGGCGAGGAACCGCGACGAGGCGGCGCCGACGGCGATCGTCGACGGCAAGCAAGTGAGGCTGTT  140
NIV1     CGGCG------AGGAACCGCGACGAGGCGGCGCCGACGGCGATCGTCGACGGCAAGCAAGTGCGGCTGTT  134
NIV2     CGGCG------AGGAACCGCGACGAGGCGGCGCCGACGGCGATCGTCGACGGCAAGCAAGTGCGGCTGTT  134
SR61     CGGCG------AGGAACCGCGACGAGGCGGCGCCGACGGCGATCGTCGACGGCAAGCAAGTGCGGCTGTT  134
RUF      CGGCG------AGGAACCGCGACGAGGCGGCGCCGACGGCGATCGTCGACGGCAAGCAAGTGCGGCTGTT  134
YJCWR    CGGCG------AGGAACCGCGACGAGGCGGCGCCGACGGTGATCGTCGACGGCAAGCAAGTGCGGCTGTT  134
 
Teqing   CCCGTGCCTCTTCTGCGCCAAGACGTTCCGCAAGTCGCAGGCGCTCGGCGGCCACCAGAACGCGCACCGG  210
HJX74    CCCGTGCCTCTTCTGCGCCAAGACGTTCCGCAAGTCGCAGGCGCTCGGCGGCCACCAGAACGCGCACCGG  210
NIV1     CCCGTGCCTCTTCTGCGAGAGGACGTTCCGCAAGTCGCAGGCGCTCGGCGGCCACCAGAACGCGCACCGG  204
NIV2     CCCGTGCCTCTTCTGCGAGAGGACGTTCCGCAAGTCGCAGGCGCTCGGCGGCCACCAGAACGCGCACCGG  204
SR61     CCCGTGCCTCTTCTGCGAGAGGACGTTCCGCAAGTCGCAGGCGCTCGGCGGCCACCAGAACGCGCACCGG  204
RUF      CCCGTGCCTCTTCTGCGAGAGGACGTTCCGCAAGTCGCAGGCGCTCGGCGGCCACCAGAACGCGCACCGG  204
YJCWR    CCCGTGCCTCTTCTGCGAGAGGACGTTCCGCAAGTCGCAGGCGCTCGGCGGCCACCAGAACGCGCACCGG  204
 
Teqing   AAGGAGCGCGTCGCCGGCGGCAGCTGGAACCCCAACGTCTACGGCGACGGCGGCGGATCAGC------GT  274
HJX74    AAGGAGCGCGTCGCCGGCGGCAGCTGGAACCCCAACGTCTACGGCGACGGCGGCGGATCAGC------GT  274
NIV1     AAGGAGCGCGTCGCCGGCGGCAGCTGGAACCCCAACGTCTACGGCGACGGCGGCGGATCAGCAGCGTCGT  274
NIV2     AAGGAGCGCGTCGCCGGCGGCAGCTGGAACCCCAACGTCTACGGCGACGGCGGCGGATCAGCAGCGTCGT  274
SR61     AAGGAGCGCGTCGCCGGCGGCAGCTGGAACCCCAACGTCTACGGCGACGGCGGCGGATCAGCAGCGTCGT  274
RUF      AAGGAGCGCGTCGCCGGCGGCAGCTGGAACCCCAACGTCTACGGCGACGGCGGCGGATCAGCAGCGTCGT  274
YJCWR    AAGGACCGCGTCGCCGGCGGCAGCTGGAACCCCAACGTCTACGGCGACAGCGGCGGATCAGCAGCGTCGT  274
 
Teqing   CCATGCCCATCGCCTCCCATGGCGTCACGGCGGCGGGGAGTAGTACGGCAGCCGACGGCCGGTGGTGCGG  344
HJX74    CCATGCCCATCGCCTCCCATGGCGTCACGGCGGCGGGGAGTAGTACGGCAGCCGACGGCCGGTGGTGCGG  344
NIV1     CCATGCCCATCGCCTCCCATGGCGTCACGGCGGCGGCGAGT---ACGGCAGCCGACGGCCGGTGGTGCGG  341
NIV2     CCATGCCCATCGCCTCCCATGGCGTCACGGCGGCGGCGAGT---ACGGCAGCCGACGGCCGGTGGTGCGG  341
SR61     CCATGCCCATCGCCTCCCATGGCGTCACGGCGGCGGCGAGT---ACGGCAGCCGACGGCCGGTGGTGCGG  341
RUF      CCATGCCCATCGCCTCCCATGGCGTCACGGCGGCGGCGAGT---ACGGCAGCCGACGGCCGGTGGTGCGG  341
YJCWR    CCATGCCCATCGCCTCCCATGGCGTCACGGCGGCGGCGAGT---ACGGCAGCCGACGGCCGGTGGTGCGG  341
 
Teqing   CGGCGCTGCCAGCGACGACG---ACACAACGGCGGCGCCCATGCCTTCCCTCGGCTCAGGCTCGGCGGCG  411
HJX74    CGGCGCTGCCAGCGACGACG---ACACAACGGCGGCGCCCATGCCTTCCCTCGGCTCAGGCTCGGCGGCG  411
NIV1     CGGCGCCGCCAGCGACGACGACGACACAACGGCGGTGCCCATGCCTTCCCTCGGCTCAGGCTCGGCGGCG  411
NIV2     CGGCGCCGCCAGCGACGACGACGACACAACGGCGGTGCCCATGCCTTCCCTCGGCTCAGGCTCGGCGGCG  411
SR61     CGGCGCCGCCAGCGACGACGACGACACAACGGCGGTGCCCATGCCTTCCCTCGGCTCAGGCTCGGCGGCG  411
RUF      CGGCGCCGCCAGCGACGACGACGACACAACGGCGGTGCCCATGCCTTCCCTCGGCTCAGGCTCGGCGGCG  411
YJCWR    CGGCGCCGCCAGCGACGACGACGACACAACGGCGGTGCCCATGCCTTCCCTCGGCTCAGGCTCGGCGGCG  411
 
Teqing   CTCGGCGCCGGCGCCGGTTTCGCTTCGACCGAAAGGGGCTCTTCCGGCGGCGGCGTCGCCGGCGAGGAGC  481
HJX74    CTCGGCGCCGGCGCCGGTTTCGCTTCGACCGAAAGGGGCTCTTCCGGCGGCGGCGTCGCCGGCGAGGAGC  481
NIV1     GGCGGCGCCG---CCGGTTTCGCTTCGACCGAAAAGGGCTCTTCCGGCG---------------AGGAGC  463
NIV2     GGCGGCGCCG---CCGGTTTCGCTTCGACCGAAAAGGGCTCTTCCGGCG---------------AGGAGC  463
SR61     GGCGGCGCCG---CCGGTTTCGCTTCGACCGAAAAGGGCTCTTCCGGCG---------------AGGAGC  463
RUF      GGCGGCGCCG---CCGGTTTCGCTTCGACCGAAAAGGGCTCTTCCGGCG---------------AGGAGC  463
YJCWR    GGCGGCGCCG---CCGGTTTCGCTTCGACCGAAAAGGGCTCTTCCGGCG---------------AGGAGC  463
 
Teqing   TTGTCCTCGAGCTCGGCCTCTAG  504
HJX74    TTGTCCTCGAGCTCGGCCTCTAG  504
NIV1     TTGTCCTCGAGCTCGGCCTCTAG  486
NIV2     TTGTCCTCGAGCTCGGCCTCTAG  486
SR61     TTGTCCTCGAGCTCGGCCTCTAG  486
RUF      TTGTCCTCGAGCTCGGCCTCTAG  486
YJCWR    TTGTCCTCGAGCTCGGCCTCTAG  486
 
Additional file 5. Coding sequence alignment of PROG1.
Teqing and YJCWR respectively mean the prog1 and PROG1 allele reported previously by Tan et al. (2008).
