# Supplementary material for: Substitution Mapping and Allelic Variations of the Domestication Genes from O. rufipogon and O. nivara
Source: Rice (N Y). 2023 Sep 5;16:38. doi: 10.1186/s12284-023-00655-y (PMC10480103; doi:10.1186/s12284-023-00655-y)
Supplement: Supplementary file 6 — Additional file 6: Amino acid sequence alignment of PROG1. [file 12284_2023_655_MOESM6_ESM.rtf]

Teqing   MDPSSASWPAPASPPVELSLSLPAAAARNRDEAAPTAIVDGKQVRLFPCLFCAKTFRKSQALGGHQNAHR  70
HJX74    MDPSSASWPAPASPPVELSLSLPAAAARNRDEAAPTAIVDGKQVRLFPCLFCAKTFRKSQALGGHQNAHR  70
NIV1     MDPSSASWPAPTPPPVELSLSLPAARNR--DEAAPTAIVDGKQVRLFPCLFCERTFRKSQALGGHQNAHR  68
NIV2     MDPSSASWPAPTPPPVELSLSLPAARNR--DEAAPTAIVDGKQVRLFPCLFCERTFRKSQALGGHQNAHR  68
SR61     MDPSSGFWPAPTPPPVELSLSLPAARNR--DEAAPTAIVDGKQVRLFPCLFCERTFRKSQALGGHQNAHR  68
RUF      MDPSSGFWPAPTPPPVELSLSLPAARNR--DEAAPTAIVDGKQVRLFPCLFCERTFRKSQALGGHQNAHR  68
YJCWR    MDPSSASWPAPTPPPVELSLSLPAARNR--DEAAPTVIVDGKQVRLFPCLFCERTFRKSQALGGHQNAHR  68
 
Teqing   KERVAGGSWNPNVYGDGGG--SASMPIASHGVTAAGSSTAADGRWCGGAASDDDTTAAPMPSLGSGSAAL  138
HJX74    KERVAGGSWNPNVYGDGGG--SASMPIASHGVTAAGSSTAADGRWCGGAASDDDTTAAPMPSLGSGSAAL  138
NIV1     KERVAGGSWNPNVYGDGGGSAASSMPIASHGVTAAASTAADGRWCGGAASDDDDTTAVPMPSLGSGSAAG  138
NIV2     KERVAGGSWNPNVYGDGGGSAASSMPIASHGVTAAASTAADGRWCGGAASDDDDTTAVPMPSLGSGSAAG  138
SR61     KERVAGGSWNPNVYGDGGGSAASSMPIASHGVTAAASTAADGRWCGGAASDDDDTTAVPMPSLGSGSAAG  138
RUF      KERVAGGSWNPNVYGDGGGSAASSMPIASHGVTAAASTAADGRWCGGAASDDDDTTAVPMPSLGSGSAAG  138
YJCWR    KDRVAGGSWNPNVYGDSGGSAASSMPIASHGVTAAASTAADGRWCGGAASDDDDTTAVPMPSLGSGSAAG  138
 
Teqing   GAGAGFASTERGSSGGGVAGEELVLELGL  167
HJX74    GAGAGFASTERGSSGGGVAGEELVLELGL  167
NIV1     GAAG------FASTEKGSSGEELVLELGL  161
NIV2     GAAG------FASTEKGSSGEELVLELGL  161
SR61     GAAG------FASTEKGSSGEELVLELGL  161
RUF      GAAG------FASTEKGSSGEELVLELGL  161
YJCWR    GAAG------FASTEKGSSGEELVLELGL  161
 

Additional file 6. Amino acid sequence alignment of PROG1.
