# Supplementary material for: Substitution Mapping and Allelic Variations of the Domestication Genes from O. rufipogon and O. nivara
Source: Rice (N Y). 2023 Sep 5;16:38. doi: 10.1186/s12284-023-00655-y (PMC10480103; doi:10.1186/s12284-023-00655-y)
Supplement: Supplementary file 7 — Additional file 7: Comparison of the main agronomic traits in SR61 and HJX74. [file 12284_2023_655_MOESM7_ESM.docx]

**Additional file 7. Comparison of the main agronomic traits in SR61 and HJX74**

Note: Sample size n=10. “*” and “**” indicated the significant difference between SR61 and HJX74 at *P* < 0.05 and *P* < 0.01, respectively.
