# Supplementary material for: Substitution Mapping and Allelic Variations of the Domestication Genes from O. rufipogon and O. nivara
Source: Rice (N Y). 2023 Sep 5;16:38. doi: 10.1186/s12284-023-00655-y (PMC10480103; doi:10.1186/s12284-023-00655-y)
Supplement: Supplementary file 8 — Additional file 8: Promoter region and genome DNA sequence alignment of TIG1. [file 12284_2023_655_MOESM8_ESM.rtf]

9311    CAGAGTACAGTGATGTAAATGTACATGAACACAGTGGAACAGCGACACACACACACACAC--CATGCACT  
HJX74   CAGAGTACAGTGATGTAAATGTACATGAACACAGTGGAACAGCGACACACACACACAC----CATGCACT  
NIV1    CAGAGTACAGTGATGTAAATGTACATGAACACAGTGGAACAGCGACACACACACACAC----CATGCACT  
NIV2    CAGAGTACAGTGATGTAAATGTACATGAACACAGTGGAACAGCGACACACACACACAC----CATGCACT  
SR76    CAGAGTGCAGTGATGTAAATGTACATGAACACAGTGGAACAGCGACACACACACACACACACCATGCACT  
RUF     CAGAGTGCAGTGATGTAAATGTACATGAACACAGTGGAACAGCGACACACACACACACACACCATGCACT  
W2014   CAGAGTACAGTGATGTAAATGTACATGAACACAGTGGAACAGCGACACACACACACAC----CATGCACT  
 
9311    TTGCTCAGCTTGTTTCAGAGCATCGATCAGCTCTGAGTCGTCTTGAGTCTTGACTACTACTCGTACTACC  
HJX74   TTGCTCAGCTTGTTTCAGAGCATCGATCAGCTCTGAGTCGTCTTGAGTCTTGACTACTACTCGTACTACC  
NIV1    TTGCTCAGCTTGTTTCAGAGCATCGATCAGCTCTGAGTCGTCTTGAGTCTTGACTACTACTCGTACTACC  
NIV2    TTGCTCAGCTTGTTTCAGAGCATCGATCAGCTCTGAGTCGTCTTGAGTCTTGACTACTACTCGTACTACC  
SR76    TTGCTCAGCTTGTTTCAGAGCATCGATCAGCTCTGAGTCGTCTTGAGTCTTGACTACTACTCGTACTACC  
RUF     TTGCTCAGCTTGTTTCAGAGCATCGATCAGCTCTGAGTCGTCTTGAGTCTTGACTACTACTCGTACTACC  
W2014   TTGCTCAGCTTGTTTCAGAGCATCGATCAGCTCTGAGTCGTCTTGAGTCTTGACTACTACTCGTACTACC  
 
9311    ACTAGCTCTCTTGAGCTTGAGCACCAGCCCAGGCTCTACTGCAGCAGCTGCCATACATTTCTAGTAGCTG  
HJX74   ACTAGCTCTCTTGAGCTTGAGCACCAGCCCAGGCTCTACTGCAGCAGCTGCCATACATTTCTAGTAGCTG  
NIV1    ACTAGCTCTCTTGAGCTTGAGCACCAGCCCAGGCTCTACTGCAGCAGCTGCCATACATTTCTAGTAGCTG  
NIV2    ACTAGCTCTCTTGAGCTTGAGCACCAGCCCAGGCTCTACTGCAGCAGCTGCCATACATTTCTAGTAGCTG  
SR76    ACTAGCTCTCTTGAGCTTGAGCACCAGCCCAGGCTCTACTGCAGCAGCTGCCATACATTTCTAGTAGCTG  
RUF     ACTAGCTCTCTTGAGCTTGAGCACCAGCCCAGGCTCTACTGCAGCAGCTGCCATACATTTCTAGTAGCTG  
W2014   ACTAGCTCTCTTGAGCTTGAGCACCAGCCCAGGCTCTACTGCAGCAGCTGCCATACATTTCTAGTAGCTG  
 
9311    ATTACATATCGTATACTTAATTAGCACTTTGCACTTGTGTACTAGTATGTGAATGTGACCATGAACACCC  
HJX74   ATTACATATCGTATACTTAATTAGCACTTTGCACTTGTGTACTAGTATGTGAATGTGACCATGAACCCCC  
NIV1    ATTACATATCGTATACTTAATTAGCACTTTGCACTTGTGTACTAGTATGTGAATGTGACCATGAACACCC  
NIV2    ATTACATATCGTATACTTAATTAGCACTTTGCACTTGTGTACTAGTATGTGAATGTGACCATGAACACCC  
SR76    ATTACATATCGTATACTTAATTAGCACTTTGCCCTTGTGTACTAGTATGTGAATGTGACCATGAACACCC  
RUF     ATTACATATCGTATACTTAATTAGCACTTTGCCCTTGTGTACTAGTATGTGAATGTGACCATGAACACCC  
W2014   ATTACATATCGTATACTTAATTAGCACTTTGCACTTGTGTACTAGTATGTGAATGTGACCATGAACACCC  
 
9311    CCCC---TCTCTCTCTCTCTCTCCTGCATCTCATCTCTTCTTTTACTACCTCTGTTTTTTAATATCTGAC  
HJX74   CCCCCCCTCTCTCTCTCTCTCTCCTGCATCTCATCTCTTCTTTTACTACCTCTGTTTTTTAATATCTGAC  
NIV1    CCCCCC-TCTCTCTCTCTCTCGCCTGCATCTCATCTCTTCTTTTACTACCTCTGTTTTTTAATATCTGAC  
NIV2    CCC----TCTCTCTCTCTCTCGCCTGCATCTCATCTCTTCTTTTACTACCTCTGTTTTTTAATATCTGAC  
SR76    CCCCCCTCCTCTCTCTCTCTCGCCTGCATCTCATCTCTTCTTTTACTATCTCTGTTTTTTAATATCTGAC  
RUF     CCCCCCTCCTCTCTCTCTCTCGCCTGCATCTCATCTCTTCTTTTACTATCTCTGTTTTTTAATATCTGAC  
W2014   CCCC--------CTCTCTCTCGCGTGCATCTCATCTCTTCTTTTACTACCTCTGTTTTTTAATATCTGAC  
 
9311    GTCGTTGACGTTTTGGCAAACGTTTAACCATTCATCTTATAAAACTTTTTTGCAAATATAAAAAAGTTTA  
HJX74   GTCGTTGACGTTTTGGCAAACGTTTAACCATTCATCTTATAAAACTTTTTTGCAAATATAAAAAAGTTTA  
NIV1    GTCGGTGACGTTTTGGCAAACGTTTAACCATTCATCTTATAAAACTTTTTTGCAAATATAAAAAAGTTTA  
NIV2    GTCGGTGACGTTGTGGCAAACGTTTAACCATTCATCTTATAAAACTTTTTTGCAAATATAAAAAAGTTTA  
SR76    GTCGTTGACGTTTTGGTAAACGTTTAACCATTCATCTTATAAAACTTTTTTGCAAATATAAAAAAGTTTA  
RUF     GTCGTTGACGTTTTGGTAAACGTTTAACCATTCATCTTATAAAACTTTTTTGCAAATATAAAAAAGTTTA  
W2014   GTTGTTGACGTTTTGGCAAACGTTTAACTATTCATCTTATAAAACTTTTTTGCAAATATAAAAAAGTTTA  
 
9311    GAGATTTTCTCCGGTCCAGCAAAAAGTACCTCGAGGTACCGGTACATTACGGTACTGAATCGTTTCCGAT  
HJX74   GAGATTTTCTCCGGTCCAGCAAAAAGTACCTCTAGGTACCGGTACATTACGGTACTGAATCGTTTCCGAT  
NIV1    GAGATTTTCTCCGGTCCAGCAAAAAGTACCTCTAGGTACCGGTACATTA---------------------  
NIV2    GAGATTTTCTCCGGTCCAGCAAAAAGTACCTCTAGGTACCGGTACATTA---------------------  
SR76    GAGATTTTCTCCGGTCTAACAAAAAATACCTCGAGGTACCGGTACATTACGGTACTGAATCGTTTCCGAT  
RUF     GAGATTTTCTCCGGTCTAACAAAAAATACCTCGAGGTACCGGTACATTACGGTACTGAATCGTTTCCGAT  
W2014   GAGATTTTCTCCGGTCCAGCAAAAAGTACCTCGAGGTACCGGTACATTACGGTACTGAATCGTTTC-GAT   
9311    CGTTGGATCTAGCTGGACAGTATGGGCATTGTTAGATCCAACAATCGAAAACGATTTGGTACCGTGAGGT  
HJX74   CGTTGGATCTAGCTGGACAGTATGGGCATTGTTAGATCCAACAATCGAAAACGATTTGGTACCGTGAGGT  
NIV1    ----------------------TGGGCATTGTTAGATCCAACAATCGAAAACGATTTGGTACCGTGAGGT  
NIV2    ----------------------TGGGCATTGTTAGATCCAACAATCGAAAACGATTTGGTACCGTGAGGT  
SR76    CGTTGGATCTAGCTGGACAGTATGGGCATTGTTAGATCCAACAATCGAAAACGATTTGGTACCGTGAGGT  
RUF     CGTTGGATCTAGCTGGACAGTATGGGCATTGTTAGATCCAACAATCGAAAACGATTTGGTACCGTGAGGT  
W2014   CGTTGGATCTAGCTGGACAGTATGGGCATTGTTAGATCCAACAATTGAAAACGATTTGGTACCGTGAGGT  
 
9311    ACCGGTAACTCGAGG-ACTTTTTATTGGACTATAGCAAATCTAAAAAATTTAAGTCATGTTTAGAAAACA  
HJX74   ACCGGTAACTCGAGGTACTTTTTATTGGACTATAGCAAATCTAAAAAATTTAAGTCATGTTTAGAAAACA  
NIV1    ACCGGTAACTCGAGGTACTTTTTATTGGACTATAGCAAATCTAAAAAATTTAAGTCATGTTTAGAAAACA  
NIV2    ACCGGTAACTCGAGGTACTTTTTATTGGACTATAGCAAATCTAAAAAATTTAAGTCATGTTTAGAAAACA 
SR76    ACCGGTACCTCGAGGTACTTTTTATTGGACTATAGCAAATCTAAAAAATTTAAGTCATGTTTAGAAAACA  
RUF     ACCGGTACCTCGAGGTACTTTTTATTGGACTATAGCAAATCTAAAAAATTTAAGTCATGTTTAGAAAACA  
W2014   ACCGGTACCTCGAGG-ACTTTTTATTGGACTATAGCAAATCTAAAAAATTTAAGTCATGTTTAGAAAACA  
 -648
9311    TTTGACGATAAATCAAGTCACAATAGATAAATGATAATTACATAAATTTTTT-GAATAAAATGAATGATC  
HJX74   TTTGACGATAAATCAAGTCACAATAGATAAATGATAATTACATAAATTTTTT-GAATAAAATGAATGATC  
NIV1    TTTGACGATAAATCAAGTCACAATAAATAAATGATAATTACATAAATTTTTT-GAATAAAATGAATGATC  
NIV2    TTTGACGATAAATCAAGTCACAATAAATAAATGATAATTACATAAATTTTTT-GAATAAAATGAATGATC  
SR76    TTTGACGATAAATCAAGTCACAATAAATAAATGATAATTACATAAATTTTTTTGAATAAAATGAATGATC  
RUF     TTTGACGATAAATCAAGTCACAATAAATAAATGATAATTACATAAATTTTTTTGAATAAAATGAATGATC  
W2014   TTTGACGATAAATCAAGTCACAATAAATAAATGATAATTACATAAATTTTTT-GAATAAAATGAATGATC  
 
9311    AATCGTATCTCAAAAAGTCAATGATGTCATATATTAAAAAACAGATGGATTAAGGTTTAGTTTTGCCCCT  
HJX74   AATCGTATCTCAAAAAGTCAATGATGTCATATATTAAAAAACAGATGGATTAAGGTTTAGTTTTGCCCCT  
NIV1    AATCGTATCTCAAAAAGTCAATGATGTCATATATTAAAAAACAGATGGATTAAGGTTTAGTTTTGCCCCT  
NIV2    AATCGTATCTCAAAAAGTCAATGATGTCATATATTAAAAAACAGATGGATTAAGGTTTAGTTTTGCCCCT  
SR76    AATCGTATCTCAAAAAGTCAATGATGTCATATATTAAAAAACAGATGGATTAAGGTTTAGTTTTGCCCCT  
RUF     AATCGTATCTCAAAAAGTCAATGATGTCATATATTAAAAAACAGATGGATTAAGGTTTAGTTTTGCCCCT  
W2014   AATCGTATCTCAAAAAGTCAATGATGTCATATATTAAAAAACAGATGGATTAAGGTTTAGTTTTGCCCCT  
 
9311    TCCATTGATCTGGTCATACCACAACACACAATTGCACACATAGAATTAAGAAGAACAAGAAGACGTGTGT  
HJX74   TCCATTGATCTGGTCATACCACAACACACAATTGCACACATAGAATTAAGAAGAACAAGAAGACGTGTGT  
NIV1    TCCATTGATCTGGTCATACCACAACACACAATTGCACACATAGAATTAAGAAGAACAAGAAGACGTGTGT  
NIV2    TCCATTGATCTGGTCATACCACAACACACAATTGCACACATAGAATTAAGAAGAACAAGAAGACGTGTGT  
SR76    TCCATTGATCTGGTCATACCACAACACACAATTGCACACATAGAATTAAGAAGAACAAGAAGACGTGTGT  
RUF     TCCATTGATCTGGTCATACCACAACACACAATTGCACACATAGAATTAAGAAGAACAAGAAGACGTGTGT  
W2014   TCCATTGATCTGGTCATACCACAACACACAATTGCACACATAGAATTAAGAAGAACAAGAAGACGTATGT  
                      -449
9311    ACAAGTGTAGTACTCTCTCCGTTTCGTAATGTAAGACTTTTGCTCATATTCATATATATGTTAATGAATC  
HJX74   ACAAGTGTAGTACTCTCTCCGTTTCGTAATGTAAGACTTTTGCTCATATTCATATATATGTTAATGAATC  
NIV1    ACAAGTGTAGTACTCCCTCTGTTTCGTAATGTAAGACTTTTGCTCATATTCATATATATGTTAATGAATC  
NIV2    ACAAGTGTAGTACTCCCTCTGTTTCGTAATGTAAGACTTTTGCTCATATTCATATATATGTTAATGAATC  
SR76    ACAAGTGTAGTACTCCCTCCATTTCGTAATGTAAGACTTTTGCTCATATTCATATATATGTTAATGAATC  
RUF     ACAAGTGTAGTACTCCCTCCATTTCGTAATGTAAGACTTTTGCTCATATTCATATATATGTTAATGAATC  
W2014   ACAAGTGTAGTACTCCCTCCGTTTCGTAATGTAAGACTTTTGCTTATATTCATATATATGTTAATGAATC  
 
9311    TAGACACATACATATATCTAGATTTATTAACTTCTATATCTAGATTCATTAACATCTATATGAATGTGGG  
HJX74   TAGACACATACATATATCTAGATTTATTAACTTCTATATCTAGATTCATTAACATCTATATGAATGTGGG  
NIV1    TAGACACATACATATATCTAGATTTATTAACTTCTATATCTAGATTCATTAACATCTATATGAATGTGGG  
NIV2    TAGACACATACATATATCTAGATTTATTAACTTCTATATCTAGATTCATTAACATCTATATGAATGTGGG  
SR76    TAGACATATACATATGTCTAGATTTATTAACTTCTATATCTAGATTCATTAACATCTATATGAATGTGGG  
RUF     TAGACATATACATATGTCTAGATTTATTAACTTCTATATCTAGATTCATTAACATCTATATGAATGTGGG  
W2014   TAGACACATACATATGTCTAGATTTATTAACTTCTATATCTAGATTCATTAACATCTATATGAATGTGGG  
 -310
9311    TAATGCTAGAAAGATTTACATTATGAAATGGAGGAAGTAGACTAGTAGTGACGGCAATGGTAGAATTATC  
HJX74   TAATGCTAGAAAGATTTACATTATGAAATGGAGGAAGTAGACTAGTAGTGACGGCAATGGTAGAATTATC  
NIV1    TAATGCTAGAAAGAGTTACATTATAAAATGGAGGAAGTAGACTAGTAGTGACGGCAATGGTAGAATTATC  
NIV2    TAATGCTAGAAAGAGTTACATTATAAAATGGAGGAAGTAGACTAGTAGTGACGGCAATGGTAGAATTATC  
SR76    TAATGCTAGAAAGACTTACATTATGAAATGGAGGAAGTAGACTAGTAGTGACGGCAATGGTAGAATTATC  
RUF     TAATGCTAGAAAGACTTACATTATGAAATGGAGGAAGTAGACTAGTAGTGACGGCAATGGTAGAATTATC  
W2014   TAATGCTAGAAAGACTTACATTATGAAATGGAGGAAGTAGACTAGTAGTGACGGCAATGGTAGAATTATC  
 
9311    ACTATCAGTTATTGCATCCATATTACATGTACTATCACTATGTTGTGTACTTGAGCTAGGAAGAGCATAA  
HJX74   ACTATCAGTTATTGCATCCATATTACATGTACTATCACTATGTTGTGTACTTGAGCTAGGAAGAGCATAA  
NIV1    ACTATCAGTTATTGCATCCATATTACATGTACTATCACTATGTTGTGTACTTGAGCTAGGAAGAGCATAA  
NIV2    ACTATCAGTTATTGCATCCATATTACATGTACTATCACTATGTTGTGTACTTGAGCTAGGAAGAGCATAA  
SR76    ACTATCAGTTATTGCATCCATATTACATGTACTATCACTATGTTGTGTACTTGAGCTAGGAAGAGCATAA  
RUF     ACTATCAGTTATTGCATCCATATTACATGTACTATCACTATGTTGTGTACTTGAGCTAGGAAGAGCATAA  
W2014   ACTATCAGTTATTGCATCCATATTACATGTACTATCACTATGTTGTGTACTTGAGCTAGGAAGAGCATAA  
 
9311    TTCAGTTTCTGCCTGATATAAAGACACACATAATCTCAAGTGGAGTTGTTATTGGTTGGGGTTAGTGCTA  
HJX74   TTCAGTTTCTGCCTGATATAAAGACACACATAATCTCAAGTGGAGTTGTTATTGGTTGGGGTTAGTGCTA  
NIV1    TTCAGTTTCTGCCTGATATAAAGACACACATAATCTCAAGTGGAGTTGTTATTGGTTGGGGTTAGTGCTA  
NIV2    TTCAGTTTCTGCCTGATATAAAGACACACATAATCTCAAGTGGAGTTGTTATTGGTTGGGGTTAGTGCTA  
SR76    TTCAGTTTCTGCCTGATATAAAGACACACATAATCTCAAGTGGAGTTGTTATTGGTTGGGGTTAGTGCTA  
RUF     TTCAGTTTCTGCCTGATATAAAGACACACATAATCTCAAGTGGAGTTGTTATTGGTTGGGGTTAGTGCTA  
W2014   TTCAGTTTCTGCCTGATATAAAGACACACATAATCTCAAGTGGAGTTGTTATTGGTTGGGGTTAGTGCTA  
 
9311    TCTTCAGCTTCACTAATGATACCTCCTTACCCTAACAACAACCTTCACCATCACCACCACCATTGCAGCT  
HJX74   TCTTCAGCTTCACTAATGATACCTCCTTACCCTAACAACAACCTTCACCATCACCACCACCATTGCAGCT  
NIV1    TCTTCAGCTTCACTAATGATACCTCCTTACCCTAACAACAACCTTCACCATCACCACCACCATTGCAGCT  
NIV2    TCTTCAGCTTCACTAATGATACCTCCTTACCCTAACAACAACCTTCACCATCACCACCACCATTGCAGCT  
SR76    TCTTCAGCTTCACTAATGATACCTCCTTACCCTAACAACAACCTTCACCATCACCACCACCATTGCAGCT  
RUF     TCTTCAGCTTCACTAATGATACCTCCTTACCCTAACAACAACCTTCACCATCACCACCACCATTGCAGCT  
W2014   TCTTCAGCTTCACTAATGATACCTCCTTACCCTAACAACAACCTTCACCATCACCACCACCATTGCAGCT  
                                                    TSS
9311    CAAACCAAGAACCACACTTACCATTACATCCACCAGCTTTCCTCATGTCCGCGCCGTCGTCGTCGTCGTC  
HJX74   CAAACCAAGAACCACACTTACCATTACATCCACCAGCTTTCCTCATGTCCGCGCCGTCGTCGTCGTCGTC  
NIV1    CAAACCAAGAACCACACTTACCATTACATCCACCAGCTTTCCTCATGTCCGCGCCGTCGTCGTCGTCGTC  
NIV2    CAAACCAAGAACCACACTTACCATTACATCCACCAGCTTTCCTCATGTCCGCGCCGTCGTCGTCGTCGTC  
SR76    CAAACCAAGAACCACACTTACCATTACATCCACCAGCTTTCCTCATGTCCGCGCCGTCGTCGTCGTCGTC  
RUF     CAAACCAAGAACCACACTTACCATTACATCCACCAGCTTTCCTCATGTCCGCGCCGTCGTCGTCGTCGTC  
W2014   CAAACCAAGAACCACACTTACCATTACATCCACCAGCTTTCCTCATGTCCGCGCCGTCGTCGTCGTCGTC  
 
9311    GCCGTCGACGCTGGACGAGTACGACGCGCGCTTCTTCTTCCCCGGCGCCGACGCGTACACCGCCGGCCAC  
HJX74   GCCGTCGACGCTGGACGAGTACGACGCGCGCTTCTTCTTCCCCGGCGCCGACGCGTACACCGCCGGCCAC  
NIV1    GCCGTCGACGCTGGACGAGTACGACGCGCGCTTCTTCTTCCCCGGCGCCGACGCGTACACCGCCGGCCAC  
NIV2    GCCGTCGACGCTGGACGAGTACGACGCGCGCTTCTTCTTCCCCGGCGCCGACGCGTACACCGCCGGCCAC  
SR76    GCCGTCGACGCTGGACGAGTACGACGCGCGCTTCTTCTTCCCCGGCGCCGACGCGTACACCCCCGGCCAC  
RUF     GCCGTCGACGCTGGACGAGTACGACGCGCGCTTCTTCTTCCCCGGCGCCGACGCGTACACCCCCGGCCAC  
W2014   GCCGTCGACGCTGGACGAGTACGACGCGCGCTTCTTCTTCCCCGGCGCCGACGCGTACACCGCCGGCCAC  
 
9311    CGGCAGGATGAGGAGACGCTGGAGGCCGTGCTGCGGCAGCCGGTGACGACGACGGCCGCGGTGGCGGCGG  
HJX74   CGGCAGGATGAGGAGACGCTGGAGGCCGTGCTGCGGCAGCCGGTGACGACGACGGCCGCGGTGGCGGCGG  
NIV1    CGGCAGGATGAGGAGACGCTGGAGGCCGTGCTGCGGCAGCCGGTGACGACGACGGCCGCGGTGGCGGCGG  
NIV2    CGGCAGGATGAGGAGACGCTGGAGGCCGTGCTGCGGCAGCCGGTGACGACGACGGCCGCGGTGGCGGCGG  
SR76    CGGCAGGATGAGGAGACGCTGGAGGCCGTGCTGCGGCAGCCGGTGACGACGACGGCCGCGGTGGCGGCGG  
RUF     CGGCAGGATGAGGAGACGCTGGAGGCCGTGCTGCGGCAGCCGGTGACGACGACGGCCGCGGTGGCGGCGG  
W2014   CGGCAGGATGAGGAGACGCTGGAGGCCGTGCTGCGGCAGCCGGTGACGACGACGGCCGCGGTGGCGGCGG 

9311    CGGCGGCGGCGGTGGAGGGAGGTGGCGGCGGTGGAGGAGGAGGCGCCGGGGGATCCCCCGCGGCGGCGGC  
HJX74   CGGCGGCGGCGGTGGAGGGAGGTGGCGGCGGTGGAGGAGGAGGCGCCGGGGGATCCCCCGCGGCGGCGGC  
NIV1    CGGCGGCGGCGGTGGAGGGAGGTGGCGGCGGTGGAGGAGGAGGCGCCGGGGGATCCCCCGCGGCGGCGGC  
NIV2    CGGCGGCGGCGGTGGAGGGAGGTGGCGGCGGTGGAGGAGGAGGCGCCGGGGGATCCCCCGCGGCGGCGGC  
SR76    AGGCGGCGGCGGTGGAGGGAGGTGGCGGCGGTGGAGGAGGAGGCGCCGGGGGATCCCCCGCGGCGGCGGC  
RUF     AGGCGGCGGCGGTGGAGGGAGGTGGCGGCGGTGGAGGAGGAGGCGCCGGGGGATCCCCCGCGGCGGCGGC  
W2014   CGGCGGCGGCGGTGGAGGGAGGTGGCGGCGGTGGAGGAGGAGGCGCCGGGGGATCCCCCGCGGCGGCGGC  
 
9311    GGCGGCGACGCGGAGGCGGCCGTTCCGGACGGACCGGCACAGCAAGATCCGCACGGCGCAGGGCGTGCGG  
HJX74   GGCGGCGACGCGGAGGCGGCCGTTCCGGACGGACCGGCACAGCAAGATCCGCACGGCGCAGGGCGTGCGG  
NIV1    GGCGGCGACGCGGAGGCGGCCGTTCCGGACGGACCGGCACAGCAAGATCCGCACGGCGCAGGGAGTGCGG  
NIV2    GGCGGCGACGCGGAGGCGGCCGTTCCGGACGGACCGGCACAGCAAGATCCGCACGGCGCAGGGAGTGCGG  
SR76    GGCGGCGACGCGGAGGCGGCCGTTCCGGACGGACCGGCACAGCAAGATCCGCACGGCGCAGGGCGTGCGG  
RUF     GGCGGCGACGCGGAGGCGGCCGTTCCGGACGGACCGGCACAGCAAGATCCGCACGGCGCAGGGCGTGCGG  
W2014   GGCGGCGACGCGGAGGCGGCCGTTCCGGACGGACCGGCACAGCAAGATCCGCACGGCGCAGGGCGTGCGG  
 
9311    GACCGGCGGATGCGGCTGTCCGTCGGCGTCGCGCGCGACTTCTTCGCGCTGCAGGACAAGCTCGGCTTCG  
HJX74   GACCGGCGGATGCGGCTGTCCGTCGGCGTCGCGCGCGACTTCTTCGCGCTGCAGGACAAGCTCGGCTTCG  
NIV1    GACCGGCGGATGCGGCTGTCCGTCGGCGTCGCGCGCGACTTCTTCGCGCTGCAGGACAAGCTCGGCTTCG  
NIV2    GACCGGCGGATGCGGCTGTCCGTCGGCGTCGCGCGCGACTTCTTCGCGCTGCAGGACAAGCTCGGCTTCG  
SR76    GACCGGCGGATGCGGCTGTCCGTCGGCGTCGCGCGCGACTTCTTCGCGCTGCAGGACAAGCTCGGCTTCG  
RUF     GACCGGCGGATGCGGCTGTCCGTCGGCGTCGCGCGCGACTTCTTCGCGCTGCAGGACAAGCTCGGCTTCG  
W2014   GACCGGCGGATGCGGCTGTCCGTCGGCGTCGCGCGCGACTTCTTCGCGCTGCAGGACAAGCTCGGCTTCG  
 
9311    ACAAGGCCAGCAGGACGGTGGAGTGGCTGCTCACCCAGTCCAAGCACGCCATCAACCGCCTCACCCTTCC  
HJX74   ACAAGGCCAGCAGGACGGTGGAGTGGCTGCTCACCCAGTCCAAGCACGCCATCAACCGCCTCACCCTTCC  
NIV1    ACAAGGCCAGCAGGACGGTGGAGTGGCTGCTCACCCAGTCCAAGCACGCCATCAACCGCCTCACCCTTCC  
NIV2    ACAAGGCCAGCAGGACGGTGGAGTGGCTGCTCACCCAGTCCAAGCACGCCATCAACCGCCTCACCCTTCC  
SR76    ACAAGGCCAGCAGGACGGTGGAGTGGCTGCTCACCCAGTCCAAGCACGCCATCAACCGCCTCACCCTTCC  
RUF     ACAAGGCCAGCAGGACGGTGGAGTGGCTGCTCACCCAGTCCAAGCACGCCATCAACCGCCTCACCCTTCC  
W2014   ACAAGGCCAGCAGGACGGTGGAGTGGCTGCTCACCCAGTCCAAGCACGCCATCAACCGCCTCACCCTTCC  
 
9311    CGACTCCGCCGACGCGGCGGCGGCGCCCGCGTTCGCCGCCGCTCCACCGCCGGCGGATCAGCATTCCTCG  
HJX74   CGACTCCGCCGACGCGGCGGCGGCGCCCGCGTTCGCCGCCGCTCCACCGCCGGCGGATCAGCATTCCTCG  
NIV1    CGACTCCGCCGACGCGGCGGCGGCGCCCGCGTTCGCCGCCGCTCCACCGCCGGCGGATCAGCATTCCTCG  
NIV2    CGACTCCGCCGACGCGGCGGCGGCGCCCGCGTTCGCCGCCGCTCCACCGCCGGCGGATCAGCATTCCTCG  
SR76    CGACTCCGCCGACGCGGCGGCGGCGCCCGCGTTCGCCGCCGCTCCACCGCCGGCGGATCAGCATTCCTCG  
RUF     CGACTCCGCCGACGCGGCGGCGGCGCCCGCGTTCGCCGCCGCTCCACCGCCGGCGGATCAGCATTCCTCG  
W2014   CGACTCCGCCGACGCGGCGGCGGCGCCCGCGTTCGCCGCCGCTCCACCGCCGGCGGATCAGCATTCCTCG  
 
9311    GCCATGGCCGCCGCCGCAGCATCGGCTGCGAAGGAGAAAGGGGAGGCGAGCTCGTCGAGCACCACCAATG  
HJX74   GCCATGGCCGCCGCCGCAGCATCGGCTGCGAAGGAGAAAGGGGAGGCGAGCTCGTCGAGCACCACCAATG  
NIV1    GCCATGGCCGCCGCCGCAGCATTGGCTGCGAAGGAGAAAGGGGAGGCGAGCTCGTCGAGCACCACCAATG  
NIV2    GCCATGGCCGCCGCCGCAGCATTGGCTGCGAAGGAGAAAGGGGAGGCGAGCTCGTCGAGCACCACCAATG  
SR76    GCCATGGCCGCCGCCGCAGCATCGGCTGCGAAGGAGAAAGGGGAGGCGAGCTCGTCGAGCACCACCAATG  
RUF     GCCATGGCCGCCGCCGCAGCATCGGCTGCGAAGGAGAAAGGGGAGGCGAGCTCGTCGAGCACCACCAATG  
W2014   GCCATGGCCGCCGCCGCAGCATCGGCTGCGAAGGAGAAAGGGGAGGCGAGCTCGTCGAGCACCACCAATG  
 
9311    CGTCGTCGGCGCGGGCGAGAAACAGAGACCACGACGGATCATCGCCGGTGGCGCCCATGGACGAGCGCGG 
HJX74   CGTCGTCGGCGCGGGCGAGAAACAGAGACCACGACGGATCATCGCCGGTGGCGCCCATGGACGAGCGCGG  
NIV1    CGTCGTCGGCGCGGGCGAGAAACAGAGACCACGACGGATCATCGCCGGTGGCGCCCATGGACGAGCGCGG  
NIV2    CGTCGTCGGCGCGGGCGAGAAACAGAGACCACGACGGATCATCGCCGGTGGCGCCCATGGACGAGCGCGG  
SR76    CGTCGTCGGCGCGGGCGAGAAACAGAGACCACGACGGATCATCGCCGGTGGCGCCCATGGACGAGCGCGG  
RUF     CGTCGTCGGCGCGGGCGAGAAACAGAGACCACGACGGATCATCGCCGGTGGCGCCCATGGACGAGCGCGG 
W2014   CGTCGTCGGCGCGGGCGAGAAACAGAGACCACGATGGATCATCGCCGGTGGCGCCCATGGACGAGCGCGG  
 
9311    GCGCCGCGGTGTCGAGCTCGACTGGACGGCGGCGGCGGCGGCGAGCACCGAGCAGCCGATGGACGGATTG  
HJX74   GCGCCGCGGTGTCGAGCTCGACTGGACGGCGGCGGCGGCGGCGAGCACCGAGCAGCCGATGGACGGATTG  
NIV1    GCGCCGCGGTGTCGAGCTCGACTGGACGGCGGCGGCGGCGGCGAGCACCGAGCAGCCGATGGACGGATTG  
NIV2    GCGCCGCGGTGTCGAGCTCGACTGGACGGCGGCGGCGGCGGCGAGCACCGAGCAGCCGATGGACGGATTG  
SR76    GCGCCGCGGTGTCGAGCTCGACTGGACGGCGGCGGCGGCGGCGAGCACCGAGCAGCCGATGGACGGATTG 
RUF     GCGCCGCGGTGTCGAGCTCGACTGGACGGCGGCGGCGGCGGCGAGCACCGAGCAGCCGATGGACGGATTG 
W2014   GCGCCGCGGTGTCGAGCTCGACTGGACGGCGGCGGCGGCGGCGAGCACCGAGCAGCCGATGGACGGATTG  
 
9311    GAGTACTACTTCCAATACTACAATCATCTGGAGGAGATAATGAGCTGCGACCCAACGACGACAACGGATG 
HJX74   GAGTACTACTTCCAATACTACAATCATCTGGAGGAGATAATGAGCTGCGACCCAACGACGACAACGGATG  
NIV1    GAGTACTACTTCCAATACTACAATCATCTGGAGGAGATAATGAGCTGCGACCCAACGACGACAACGGATG  
NIV2    GAGTACTACTTCCAATACTACAATCATCTGGAGGAGATAATGAGCTGCGACCCAACGACGACAACGGATG  
SR76    GAGTACTACTTCCAATACTACAATCATCTGGAGGAGATAATGAGCTGCGACCCAACGACGACAACGGATG  
RUF     GAGTACTACTTCCAATACTACAATCATCTGGAGGAGATAATGAGCTGCGACCCAACGACGACAACGGATG 
W2014   GAGTACTACTTCCAATACTACAATCATCTGGAGGAGATAATGAGCTGCGACCCAACGACGACAACGGATG  
 
9311    AGTAAAGCCAGTAAAGGTGATTTCTTGTGTTAAACAATCAAACAAAAATA  
HJX74   AGTAAAGCCAGTAAAGGTGATTTCTTGTGTTAAACAATCAAACAAAAATA  
NIV1    AGTAAAGCCAGTAAAGGTGATTTCTTGTGTTAAACAATCAAACAAAAATA  
NIV2    AGTAAAGCCAGTAAAGGTGATTTCTTGTGTTAAACAATCAAACAAAAATA  
SR76    AGTAAAGCCAGTAAAGGTGATTTCTTGTGTTAAACAATCAAACAAAAATA  
RUF     AGTAAAGCCAGTAAAGGTGATTTCTTGTGTTAAACAATCAAACAAAAATA  
W2014   AGTAAAGCCAGTAAAGGTGATTTCTTGTGTTAAACAATCAAACAAAAATA  
 
Additional file 8. Promoter region and DNA sequence alignment of TIG1.
Red box means the key SNP reported previously by Zhang et al. (2019). 9311 and W2014 represent the tig1 and TIG1 allele. TSS means transcriptional start site.
