# Supplementary material for: Substitution Mapping and Allelic Variations of the Domestication Genes from O. rufipogon and O. nivara
Source: Rice (N Y). 2023 Sep 5;16:38. doi: 10.1186/s12284-023-00655-y (PMC10480103; doi:10.1186/s12284-023-00655-y)
Supplement: Supplementary file 9 — Additional file 9: Amino acid sequence alignment of TIG1. [file 12284_2023_655_MOESM9_ESM.rtf]

9311    MSAPSSSSSPSTLDEYDARFFFPGADAYTAGHRQDEETLEAVLRQPVTTTAAVAAAAAAVEGGGGGGGGG  70
HJX74   MSAPSSSSSPSTLDEYDARFFFPGADAYTAGHRQDEETLEAVLRQPVTTTAAVAAAAAAVEGGGGGGGGG  70
NIV1    MSAPSSSSSPSTLDEYDARFFFPGADAYTAGHRQDEETLEAVLRQPVTTTAAVAAAAAAVEGGGGGGGGG  70
NIV2    MSAPSSSSSPSTLDEYDARFFFPGADAYTAGHRQDEETLEAVLRQPVTTTAAVAAAAAAVEGGGGGGGGG  70
SR76    MSAPSSSSSPSTLDEYDARFFFPGADAYTPGHRQDEETLEAVLRQPVTTTAAVAAEAAAVEGGGGGGGGG  70
RUF     MSAPSSSSSPSTLDEYDARFFFPGADAYTPGHRQDEETLEAVLRQPVTTTAAVAAEAAAVEGGGGGGGGG  70
W2014   MSAPSSSSSPSTLDEYDARFFFPGADAYTPGHRQDEETLEAVLRQPVTTTAAVAAEAAAVEGGGGGGGGG  70
 
9311    AGGSPAAAAAATRRRPFRTDRHSKIRTAQGVRDRRMRLSVGVARDFFALQDKLGFDKASRTVEWLLTQSK  140
HJX74   AGGSPAAAAAATRRRPFRTDRHSKIRTAQGVRDRRMRLSVGVARDFFALQDKLGFDKASRTVEWLLTQSK  140
NIV1    AGGSPAAAAAATRRRPFRTDRHSKIRTAQGVRDRRMRLSVGVARDFFALQDKLGFDKASRTVEWLLTQSK  140
NIV2    AGGSPAAAAAATRRRPFRTDRHSKIRTAQGVRDRRMRLSVGVARDFFALQDKLGFDKASRTVEWLLTQSK  140
SR76    AGGSPAAAAAATRRRPFRTDRHSKIRTAQGVRDRRMRLSVGVARDFFALQDKLGFDKASRTVEWLLTQSK  140
RUF     AGGSPAAAAAATRRRPFRTDRHSKIRTAQGVRDRRMRLSVGVARDFFALQDKLGFDKASRTVEWLLTQSK  140
W2014   AGGSPAAAAAATRRRPFRTDRHSKIRTAQGVRDRRMRLSVGVARDFFALQDKLGFDKASRTVEWLLTQSK  140
 
9311    HAINRLTLPDSADAAAAPAFAAAPPPADQHSSAMAAAAASAAKEKGEASSSSTTNASSARARNRDHDGSS  210
HJX74   HAINRLTLPDSADAAAAPAFAAAPPPADQHSSAMAAAAASAAKEKGEASSSSTTNASSARARNRDHDGSS  210
NIV1    HAINRLTLPDSADAAAAPAFAAAPPPADQHSSAMAAAAALAAKEKGEASSSSTTNASSARARNRDHDGSS  210
NIV2    HAINRLTLPDSADAAAAPAFAAAPPPADQHSSAMAAAAALAAKEKGEASSSSTTNASSARARNRDHDGSS  210
SR76    HAINRLTLPDSADAAAAPAFAAAPPPADQHSSAMAAAAASAAKEKGEASSSSTTNASSARARNRDHDGSS  210
RUF     HAINRLTLPDSADAAAAPAFAAAPPPADQHSSAMAAAAASAAKEKGEASSSSTTNASSARARNRDHDGSS  210
W2014   HAINRLTLPDSADAAAAPAFAAAPPPADQHSSAMAAAAASAAKEKGEASSSSTTNASSARARNRDHDGSS  210
 
9311    PVAPMDERGRRGVELDWTAAAAASTEQPMDGLEYYFQYYNHLEEIMSCDPTTTTDE  266
HJX74   PVAPMDERGRRGVELDWTAAAAASTEQPMDGLEYYFQYYNHLEEIMSCDPTTTTDE  266
NIV1    PVAPMDERGRRGVELDWTAAAAASTEQPMDGLEYYFQYYNHLEEIMSCDPTTTTDE  266
NIV2    PVAPMDERGRRGVELDWTAAAAASTEQPMDGLEYYFQYYNHLEEIMSCDPTTTTDE  266
SR76    PVAPMDERGRRGVELDWTAAAAASTEQPMDGLEYYFQYYNHLEEIMSCDPTTTTDE  266
RUF     PVAPMDERGRRGVELDWTAAAAASTEQPMDGLEYYFQYYNHLEEIMSCDPTTTTDE  266
W2014   PVAPMDERGRRGVELDWTAAAAASTEQPMDGLEYYFQYYNHLEEIMSCDPTTTTDE  266
 
Additional file 9. Amino acid sequence alignment of TIG1.
