# Supplementary material for: Substitution Mapping and Allelic Variations of the Domestication Genes from O. rufipogon and O. nivara
Source: Rice (N Y). 2023 Sep 5;16:38. doi: 10.1186/s12284-023-00655-y (PMC10480103; doi:10.1186/s12284-023-00655-y)
Supplement: Supplementary file 10 — Additional file10: CDS alignment of OsLG1. [file 12284_2023_655_MOESM10_ESM.rtf]

HJX74   ATGATGAACGTTCCATCCGCCGCTGCCGCGAGCTCCTGCGATGATTTCGGCTACAACGCCACCCCGCCGC  70
SN57    ATGATGAACGTTCCATCCGCCGCTGCCGCGAGCTCCTGCGATGATTTCGGCTACAACGCCACCCCGCCGC  70
NIV1    ATGATGAACGTTCCATCCGCCGCTGCCGCGAGCTCCTGCGATGATTTCGGCTACAACGCCACCCCGCCGC  70
NIV2    ATGATGAACGTTCCATCCGCCGCTGCCGCGAGCTCCTGCGATGATTTCGGCTACAACGCCACCCCGCCGC  70
RUF     ATGATGAACGTTCCATCCGCCGCTGCCGCGAGCTCCTGCGATGATTTCGGCTACAACGCCACCCCGCCGC  70
 
HJX74   CGCCGCCGTCGCTTCTCCCAATCATGGACCAGGACGGCGGCGGCGGTAGCATCCAGAGGGATCACCACCA  140
SN57    CGCCGCCGTCGCTTCTCCCAATCATGGACCAGGACGGCGGCGGCGGTAACATCCAGAGGGATCACCACCA  140
NIV1    CGCCGCCGTCGCTTCTCCCAATCATGGACCAGGACGGCGGCGGCGGTAACATCCAGAGGGATCACCACCA  140
NIV2    CGCCGCCGTCGCTTCTCCCAATCATGGACCAGGACGGCGGCGGCGGTAACATCCAGAGGGATCACCACCA  140
RUF     CGCCGCCGTCGCTTCTCCCAATCATGGACCAGGACGGCGGCGGCGGTAGCATCCAGAGGGATCACCACCA  140
 
HJX74   CCACCACAACCACCAGCAGCTCGGCTACAACCTGGAGCCGAGCTCCCTAGCTCTGCTTCCCCCTTCCAAC  210
SN57    CCACCAC---CACCAGCAGCTCGGCTACAACCTGGAGCCGAGCTCCCTAGCTCTGCTTCCCCCTTCCAAC  207
NIV1    CCACCAC---CACCAGCAGCTCGGCTACAACCTGGAGCCGAGCTCCCTAGCTCTGCTTCCCCCTTCCAAC  207
NIV2    CCACCAC---CACCAGCAGCTCGGCTACAACCTGGAGCCGAGCTCCCTAGCTCTGCTTCCCCCTTCCAAC  207
RUF     CCACCACCACCAGCAGCAGCTCGGCTACAACCTGGAGCCGAGCTCCCTAGCTCTGCTTCCCCCTTCCAAC  210
 
HJX74   GCCGCCGCCGCCGCAGCACACCACGCCACCATCGCCCACGCCTCCCCACATGACCTCCTCCAGTTCTACC  280
SN57    GCCGCCGCCGCCGCAGCACACCACGCCACCATCGCCCACGCCTCCCCACATGACCTCCTCCAGTTCTACC  277
NIV1    GCCGCCGCCGCCGCAGCACACCACGCCACCATCGCCCACGCCTCCCCACATGACCTCCTCCAGTTCTACC  277
NIV2    GCCGCCGCCGCCGCAGCACACCACGCCACCATCGCCCACGCCTCCCCACATGACCTCCTCCAGTTCTACC  277
RUF     GCCGCCGCCGCCGCAGCACACCACGCCACCATCGCCCACGCCTCCCCACATGACCTCCTCCAGTTCTACC  280
 
HJX74   CGACCTCGCACTACCTCGCCGCTGCCGGCGGCGCCGGTGGCGGAGGCAACCCCTACAGCCACTTCACGGC  350
SN57    CGACCTCGCACTACCTCGCCGCTGCCGGCGGCGCCGGTGGCGGAGGCAACCCCTACAGCCACTTCACGGC  347
NIV1    CGACCTCGCACTACCTCGCCGCTGCCGGCGGCGCCGGTGGCGGAGGCAACCCCTACAGCCACTTCACGGC  347
NIV2    CGACCTCGCACTACCTCGCCGCTGCCGGCGGCGCCGGTGGCGGAGGCAACCCCTACAGCCACTTCACGGC  347
RUF     CGACCTCGCACTACCTCGCCGCTGCCGGCGGCGCCGGTGGCGGAGGCAACCCCTACAGCCACTTCACGGC  350
 
HJX74   GGCGGCGGCGGCCGGGAGCACCTTCCAGTCGTACTACCAGCAGCCGCCGCAGGCCGCGCCGGAGTACTAC  420
SN57    GGCGGCGGCGGCCGGGAGCACCTTCCAGTCGTACTACCAGCAGCCGCCGCAGGCCGCGCCGGAGTACTAC  417
NIV1    GGCGGCGGCGGCCGGGAGCACCTTCCAGTCGTACTACCAGCAGCCGCCGCAGGCCGCGCCGGAGTACTAC  417
NIV2    GGCGGCGGCGGCCGGGAGCACCTTCCAGTCGTACTACCAGCAGCCGCCGCAGGCCGCGCCGGAGTACTAC  417
RUF     GGCGGCGGCGGCCGGGAGCACCTTCCAGTCGTACTACCAGCAGCCGCCGCAGGCCGCGCCGGAGTACTAC  420
 
HJX74   TTCCCGACGCTGGTCAGCTCCGCCGAGGAGAACATGGCCAGCTTCGCCGCCACCCAGCTCGGCCTCAACC  490
SN57    TTCCCGACGCTGGTCAGCTCCGCCGAGGAGAACATGGCCAGCTTCGCCGCCACCCAGCTCGGCCTCAACC  487
NIV1    TTCCCGACGCTGGTCAGCTCCGCCGAGGAGAACATGGCCAGCTTCGCCGCCACCCAGCTCGGCCTCAACC  487
NIV2    TTCCCGACGCTGGTCAGCTCCGCCGAGGAGAACATGGCCAGCTTCGCCGCCACCCAGCTCGGCCTCAACC  487
RUF     TTCCCGACGCTGGTCAGCTCCGCCGAGGAGAACATGGCCAGCTTCGCCGCCACCCAGCTCGGCCTCAACC  490
 
HJX74   TCGGCTACCGGACCTACTTCCCGCCGAGAGGCGGCTACACCTACGGCCACCACCCGCCGCGGTGCCAGGC  560
SN57    TCGGCTACCGGACCTACTTCCCGCCGAGAGGCGGCTACACCTACGGCCACCACCCGCCGCGGTGCCAGGC  557
NIV1    TCGGCTACCGGACCTACTTCCCGCCGAGAGGCGGCTACACCTACGGCCACCACCCGCCGCGGTGCCAGGC  557
NIV2    TCGGCTACCGGACCTACTTCCCGCCGAGAGGCGGCTACACCTACGGCCACCACCCGCCGCGGTGCCAGGC  557
RUF     TCGGCTACCGGACCTACTTCCCGCCGAGAGGCGGCTACACCTACGGCCACCACCCGCCGCGGTGCCAGGC  560
 
HJX74   CGAGGGCTGCAAGGCCGACCTCTCCAGCGCCAAGCGCTACCACCGCCGCCACAAGGTGTGCGAGCACCAC  630
SN57    CGAGGGCTGCAAGGCCGACCTCTCCAGCGCCAAGCGCTACCACCGCCGCCACAAGGTGTGCGAGCACCAC  627
NIV1    CGAGGGCTGCAAGGCCGACCTCTCCAGCGCCAAGCGCTACCACCGCCGCCACAAGGTGTGCGAGCACCAC  627
NIV2    CGAGGGCTGCAAGGCCGACCTCTCCAGCGCCAAGCGCTACCACCGCCGCCACAAGGTGTGCGAGCACCAC  627
RUF     CGAGGGCTGCAAGGCCGACCTCTCCAGCGCCAAGCGCTACCACCGCCGCCACAAGGTGTGCGAGCACCAC  630
 
HJX74   TCCAAGGCGCCCGTCGTCGTCACCGCCGGCGGCCTCCACCAGAGATTCTGCCAGCAGTGCAGCAGATTCC  700
SN57    TCCAAGGCGCCCGTCGTCGTCACCGCCGGCGGCCTCCACCAGAGATTCTGCCAGCAGTGCAGCAGATTCC  697
NIV1    TCCAAGGCGCCCGTCGTCGTCACCGCCGGCGGCCTCCACCAGAGATTCTGCCAGCAGTGCAGCAGATTCC  697
NIV2    TCCAAGGCGCCCGTCGTCGTCACCGCCGGCGGCCTCCACCAGAGATTCTGCCAGCAGTGCAGCAGATTCC  697
RUF     TCCAAGGCGCCCGTCGTCGTCACCGCCGGCGGCCTCCACCAGAGATTCTGCCAGCAGTGCAGCAGATTCC  700
 
HJX74   ATCTTCTTGATGAGTTCGACGATGCCAAGAAGAGCTGCAGGAAGCGACTCGCCGACCACAACCGCCGGCG  770
SN57    ATCTTCTTGATGAGTTCGACGATGCCAAGAAGAGCTGCAGGAAGCGACTCGCCGACCACAACCGCCGGCG  767
NIV1    ATCTTCTTGATGAGTTCGACGATGCCAAGAAGAGCTGCAGGAAGCGACTCGCCGACCACAACCGCCGGCG  767
NIV2    ATCTTCTTGATGAGTTCGACGATGCCAAGAAGAGCTGCAGGAAGCGACTCGCCGACCACAACCGCCGGCG  767
RUF     ATCTTCTTGATGAGTTCGACGATGCCAAGAAGAGCTGCAGGAAGCGACTCGCCGACCACAACCGCCGGCG  770
 
HJX74   GAGGAAGTCGAAGCCGTCCGACGGCGAGCATTCTGGTGAAAAGAGAAGGGCGCAGGCGAATAAATCGGCA  840
SN57    GAGGAAGTCGAAGCCGTCCGACGGCGAGCATTCTGGTGAAAAGAGAAGGGCGCAGGCGAATAAATCGGCA  837
NIV1    GAGGAAGTCGAAGCCGTCCGACGGCGAGCATTCTGGTGAAAAGAGAAGGGCGCAGGCGAATAAATCGGCA  837
NIV2    GAGGAAGTCGAAGCCGTCCGACGGCGAGCATTCTGGTGAAAAGAGAAGGGCGCAGGCGAATAAATCGGCA  837
RUF     GAGGAAGTCGAAGCCGTCCGACGGCGAGCATTCTGGTGAAAAGAGAAGGGCGCAGGCGAATAAATCGGCA  840
 
HJX74   GCTACTAAAGACAAAGCAGGAAGTAGCAGCAAGAACGCAGGCATTGGAGACGGTTTCGAGACACAGCTAC  910
SN57    GCTACTAAAGACAAAGCAGGAAGTAGCAGCAAGAACGCAGGCATTGGAGACGGTTTCGAGACACAGCTAC  907
NIV1    GCTACTAAAGACAAAGCAGGAAGTAGCAGCAAGAACGCAGGCATTGGAGACGGTTTCGAGACACAGCTAC  907
NIV2    GCTACTAAAGACAAAGCAGGAAGTAGCAGCAAGAACGCAGGCATTGGAGACGGTTTCGAGACACAGCTAC  907
RUF     GCTACTAAAGACAAAGCAGGAAGTAGCAGCAAGAACGCAGGCATTGGAGACGGTTTCGAGACACACATAC  910
 
HJX74   TGGGGGGTGCACACATGTCCAAAGATCAAGACCAAGCCATGGATCTGGGAGAGGTGGTGAAAGAAGCTGT  980
SN57    TGGGGGGTGCACACATGTCCAAAGATCAAGACCAAGCCATGGATCTGGGAGAGGTGGTGAAAGAAGCTGT  977
NIV1    TGGGGGGTGCACACATGTCCAAAGATCAAGACCAAGCCATGGATCTGGGAGAGGTGGTGAAAGAAGCTGT  977
NIV2    TGGGGGGTGCACACATGTCCAAAGATCAAGACCAAGCCATGGATCTGGGAGAGGTGGTGAAAGAAGCTGT  977
RUF     TGGGGGGGGCACACACGTCCAAAGATCAAGACCAAGCCACGGATCTGGGAGAGGTGGTGAAAGAAGCTGT  980
 
HJX74   AGATCCCAAAGGTAAGGCATCGATGCAGCAGCAGCAGCAGCAAGCACATCATGGGATTCATCAGCAGAGC  1050
SN57    AGATCCCAAAGGTAAGGCATCGATGCAGCAGCAGCAGCAGCAAGCACATCATGGGATTCATCAGCAGAGC  1047
NIV1    AGATCCCAAAGGTAAGGCATCGATGCAGCAGCAGCAGCAGCAAGCACATCATGGGATTCATCAGCAGAGC  1047
NIV2    AGATCCCAAAGGTAAGGCATCGATGCAGCAGCAGCAGCAGCAAGCACATCATGGGATTCATCAGCAGAGC  1047
RUF     AGATCCCACAGGTAAGGCATCGATGCAGCAGCAGCAGCAGCAAGCACATCATGGGATTCATCAGCAGAGA  1050
 
HJX74   CACCAGCAGCATGGCTTCCCTTTCCCTTCGTCGTCTGGCTCGTGCTTATTCCCTCAGAGCCAAGGAGCTG  1120
SN57    CACCAGCAGCATGGCTTCCCTTTCCCTTCGTCGTCTGGCTCGTGCTTATTCCCTCAGAGCCAAGGAGCTG  1117
NIV1    CACCAGCAGCATGGCTTCCCTTTCCCTTCGTCGTCTGGCTCGTGCTTATTCCCTCAGAGCCAAGGAGCTG  1117
NIV2    CACCAGCAGCATGGCTTCCCTTTCCCTTCGTCGTCTGGCTCGTGCTTATTCCCTCAGAGCCAAGGAGCTG  1117
RUF     CACCAGCAGCATGGCTTCCCTTTCCCTTCGTCGTCTGGCTCGTGCTTATTCCCTCAGAGACAAGGAGCTG  1120
 
HJX74   TCTCGAGCACTGACACATCAAATATAGCTCAAGTGCAAGAACCAAGCTTAGCCTTCCATCAGCAGCATCA  1190
SN57    TCTCGAGCACTGACACATCAAATATAGCTCAGGTGCAAGAACCAAGCTTAGCCTTCCATCAGCAGCATCA  1187
NIV1    TCTCGAGCACTGACACATCAAATATAGCTCAGGTGCAAGAACCAAGCTTAGCCTTCCATCAGCAGCATCA  1187
NIV2    TCTCGAGCACTGACACATCAAATATAGCTCAGGTGCAAGAACCAAGCTTAGCCTTCCATCAGCAGCATCA  1187
RUF     TCTCGAGCGCTGACACATCAAATATAGCTCAGGTGCAAGAGCCAAGCTTAGCCTTCCATCAGCAGCATCT  1190
 
HJX74   CCAACACAGCAACATCCTTCAGCTTGGACAGGCGATGTTTGATCTCGACTTCGATCACTAG  1251
SN57    CCAACACAGCAACATCCTTCAGCTTGGACAGGCGATGTTTGATCTCGACTTCGATCACTAG  1248
NIV1    CCAACACAGCAACATCCTTCAGCTTGGACAGGCGATGTTTGATCTCGACTTCGATCACTAG  1248
NIV2    CCAACACAGCAACATCCTTCAGCTTGGACAGGCGATGTTTGATCTCGACTTCGATCACTAG  1248
RUF     CCAACACAGCAACATCCTTCAGCTTGGACAGGCGATGTGTGATCTCGACTTCGATCACTAG  1251
 
Additional file 10. CDS alignment of OsLG1
