# Supplementary material for: Substitution Mapping and Allelic Variations of the Domestication Genes from O. rufipogon and O. nivara
Source: Rice (N Y). 2023 Sep 5;16:38. doi: 10.1186/s12284-023-00655-y (PMC10480103; doi:10.1186/s12284-023-00655-y)
Supplement: Supplementary file 11 — Additional file 11: Amino acid sequence alignment of OsLG1. [file 12284_2023_655_MOESM11_ESM.rtf]

HJX74   MMNVPSAAAASSCDDFGYNATPPPPPSLLPIMDQDGGGGSIQRDHHHHHNHQQLGYNLEPSSLALLPPSN  70
SN57    MMNVPSAAAASSCDDFGYNATPPPPPSLLPIMDQDGGGGNIQRDHHHHH-HQQLGYNLEPSSLALLPPSN  69
NIV1    MMNVPSAAAASSCDDFGYNATPPPPPSLLPIMDQDGGGGNIQRDHHHHH-HQQLGYNLEPSSLALLPPSN  69
NIV2    MMNVPSAAAASSCDDFGYNATPPPPPSLLPIMDQDGGGGNIQRDHHHHH-HQQLGYNLEPSSLALLPPSN  69
RUF     MMNVPSAAAASSCDDFGYNATPPPPPSLLPIMDQDGGGGSIQRDHHHHHHQQQLGYNLEPSSLALLPPSN  70
 
HJX74   AAAAAAHHATIAHASPHDLLQFYPTSHYLAAAGGAGGGGNPYSHFTAAAAAGSTFQSYYQQPPQAAPEYY  140
SN57    AAAAAAHHATIAHASPHDLLQFYPTSHYLAAAGGAGGGGNPYSHFTAAAAAGSTFQSYYQQPPQAAPEYY  139
NIV1    AAAAAAHHATIAHASPHDLLQFYPTSHYLAAAGGAGGGGNPYSHFTAAAAAGSTFQSYYQQPPQAAPEYY  139
NIV2    AAAAAAHHATIAHASPHDLLQFYPTSHYLAAAGGAGGGGNPYSHFTAAAAAGSTFQSYYQQPPQAAPEYY  139
RUF     AAAAAAHHATIAHASPHDLLQFYPTSHYLAAAGGAGGGGNPYSHFTAAAAAGSTFQSYYQQPPQAAPEYY  140
 
HJX74   FPTLVSSAEENMASFAATQLGLNLGYRTYFPPRGGYTYGHHPPRCQAEGCKADLSSAKRYHRRHKVCEHH  210
SN57    FPTLVSSAEENMASFAATQLGLNLGYRTYFPPRGGYTYGHHPPRCQAEGCKADLSSAKRYHRRHKVCEHH  209
NIV1    FPTLVSSAEENMASFAATQLGLNLGYRTYFPPRGGYTYGHHPPRCQAEGCKADLSSAKRYHRRHKVCEHH  209
NIV2    FPTLVSSAEENMASFAATQLGLNLGYRTYFPPRGGYTYGHHPPRCQAEGCKADLSSAKRYHRRHKVCEHH  209
RUF     FPTLVSSAEENMASFAATQLGLNLGYRTYFPPRGGYTYGHHPPRCQAEGCKADLSSAKRYHRRHKVCEHH  210
 
HJX74   SKAPVVVTAGGLHQRFCQQCSRFHLLDEFDDAKKSCRKRLADHNRRRRKSKPSDGEHSGEKRRAQANKSA  280
SN57    SKAPVVVTAGGLHQRFCQQCSRFHLLDEFDDAKKSCRKRLADHNRRRRKSKPSDGEHSGEKRRAQANKSA  279
NIV1    SKAPVVVTAGGLHQRFCQQCSRFHLLDEFDDAKKSCRKRLADHNRRRRKSKPSDGEHSGEKRRAQANKSA  279
NIV2    SKAPVVVTAGGLHQRFCQQCSRFHLLDEFDDAKKSCRKRLADHNRRRRKSKPSDGEHSGEKRRAQANKSA  279
RUF     SKAPVVVTAGGLHQRFCQQCSRFHLLDEFDDAKKSCRKRLADHNRRRRKSKPSDGEHSGEKRRAQANKSA  280
 
HJX74   ATKDKAGSSSKNAGIGDGFETQLLGGAHMSKDQDQAMDLGEVVKEAVDPKGKASMQQQQQQAHHGIHQQS  350
SN57    ATKDKAGSSSKNAGIGDGFETQLLGGAHMSKDQDQAMDLGEVVKEAVDPKGKASMQQQQQQAHHGIHQQS  349
NIV1    ATKDKAGSSSKNAGIGDGFETQLLGGAHMSKDQDQAMDLGEVVKEAVDPKGKASMQQQQQQAHHGIHQQS  349
NIV2    ATKDKAGSSSKNAGIGDGFETQLLGGAHMSKDQDQAMDLGEVVKEAVDPKGKASMQQQQQQAHHGIHQQS  349
RUF     ATKDKAGSSSKNAGIGDGFETHILGGAHTSKDQDQATDLGEVVKEAVDPTGKASMQQQQQQAHHGIHQQR  350
 
HJX74   HQQHGFPFPSSSGSCLFPQSQGAVSSTDTSNIAQVQEPSLAFHQQHHQHSNILQLGQAMFDLDFDH  416
SN57    HQQHGFPFPSSSGSCLFPQSQGAVSSTDTSNIAQVQEPSLAFHQQHHQHSNILQLGQAMFDLDFDH  415
NIV1    HQQHGFPFPSSSGSCLFPQSQGAVSSTDTSNIAQVQEPSLAFHQQHHQHSNILQLGQAMFDLDFDH  415
NIV2    HQQHGFPFPSSSGSCLFPQSQGAVSSTDTSNIAQVQEPSLAFHQQHHQHSNILQLGQAMFDLDFDH  415
RUF     HQQHGFPFPSSSGSCLFPQRQGAVSSADTSNIAQVQEPSLAFHQQHLQHSNILQLGQAMCDLDFDH  416
 
Additional file 11. Amino acid sequence alignment of OsLG1
