# Supplementary material for: Substitution Mapping and Allelic Variations of the Domestication Genes from O. rufipogon and O. nivara
Source: Rice (N Y). 2023 Sep 5;16:38. doi: 10.1186/s12284-023-00655-y (PMC10480103; doi:10.1186/s12284-023-00655-y)
Supplement: Supplementary file 12 — Additional file 12: Alignment of key promoter sequence of OsLG1. [file 12284_2023_655_MOESM12_ESM.rtf]

Teqing   TCTGTTTGGGGGAGCTTCTAGTTGCT-GTAGCTACTCCTAAAATCAGAAGCTCCCCAAAACAGTCTAGCT  69
HJX74    TCTGTTTGGGGGAGCTTCTAGTTGCT-GTAGCTACTCCTAAAATCAGAAGCTCCCCAAAACAGTCTAGCT  69
SN57     TCTGTTTGGGGGAGCTTCTAGTTGCT-GTAGCTTCTCCTAAAATCAGAAGCTCCCCAAAACAGTCTAGCT  69
NIV1     TCTGTTTGGGGGAGCTTCTAGTTGCT-GTAGCTTCTCCTAAAATCAGAAGCTCCCCAAAACAGTCTAGCT  69
NIV2     TCTGTTTGGGGGAGCTTCTAGTTGCT-GTAGCTTCTCCTAAAATCAGAAGCTCCCCAAAACAGTCTAGCT  69
RUF      TCTGTTTAGGGAAGCTTCTAGTTACTTATAGCTTCTCCTAAAATCAGAAGCTCCCCAAAACAGTCTAGCT  70
YJCWR    TCTGTTTGGGGGAGCTTCTAGTTGCT-GTAGCTACTCCTAAAATCAGAAGCTCCCTAAAACAGTCTAGCT  69
 
Teqing   TTTGGTCCAGATTTGAGAAGCTGTAATTGTAGAATCTAGAAAATGAACTAGCAGCCAGAAGCTGGGAAAC  139
HJX74    TTTGGTCCAGATTTGAGAAGCTGTAATTGTAGAATCTAGAAAATGAACTAGCAGCCAGAAGCTGGGAAAC  139
SN57     TTTGGTCCAGATTTGAGAAGCTGTAATTGTAGAATCTAGAAAATAAAATAGCAGCCAGAAGCTGGGAAAC  139
NIV1     TTTGGTCCAGATTTGAGAAGCTGTAATTGTAGAATCTAGAAAATAAAATAGCAGCCAGAAGCTGGGAAAC  139
NIV2     TTTGGTCCAGATTTGAGAAGCTGTAATTGTAGAATCTAGAAAATAAAATAGCAGCCAGAAGCTGGGAAAC  139
RUF      TTTGGTCCAGATTTGAGAAGCTGTAATTGTAGAATCTAGAAAATGAACTAGCAGCCAGAAGCTGGGAAAC  140
YJCWR    TTTGGTCCAGATTTGAGAAGCTGTAATTGTAGAATCTAGAAAATGAACTAGCAGCCAGAAGCTGGGAAAC  139
 
Teqing   CCAGCTTTTCCAGATTCTCAGAAGCTGGCTACCAATCAACTGCTTCTTCGAATTTTAAGCTCCCTCAAAC  209
HJX74    CCAGCTTTTCCAGATTCTCAGAAGCTGGCTACCAATCAACTGCTTCTTCGAATTTTAAGCTCCCTCAAAC  209
SN57     CCAGCTTTTCCAGATTCTTAGAAGCTGGCTACCAAACTACTGCTTCTTCGAATTTTAAGCTCCCTCAAAC  209
NIV1     CCAGCTTTTCCAGATTCTTAGAAGCTGGCTACCAAACTACTGCTTCTTCGAATTTTAAGCTCCCTCAAAC  209
NIV2     CCAGCTTTTCCAGATTCTTAGAAGCTGGCTACCAAACTACTGCTTCTTCGAATTTTAAGCTCCCTCAAAC  209
RUF      CCAGCTTTTCCAGATTCTCAGAAGCTGGCTACCAAACAACTGCTTCTTCGAATTTTAAGCTCCCTCAAAC  210
YJCWR    CCAGCTTTTCCAGATTCTCAGAAGCTGGCTACCAATCAACTGCTTCTTCGAATTTTAAGCTCCCTCAAAC  209
 
Teqing   AGGCCCAAAGTCTTGTACATAGACAAGGAATATGAAATCTTTTGTTTGAAAACTTAAGTTAGGTATTAGC  279
HJX74    AGGCCCAAAGTCTTGTACATAGACAAGGAATATGAAATCTTTTGTTTGAAAACTTAAGTTAGGTATTAGC  279
SN57     AGGCCCAAAGTCTTGTACATAGACAAGGAATATGAAATCTTTTGTTTGAAAACTTAAGTTAGGTATTAGC  279
NIV1     AGGCCCAAAGTCTTGTACATAGACAAGGAATATGAAATCTTTTGTTTGAAAACTTAAGTTAGGTATTAGC  279
NIV2     AGGCCCAAAGTCTTGTACATAGACAAGGAATATGAAATCTTTTGTTTGAAAACTTAAGTTAGGTATTAGC  279
RUF      AGGCCCAAATTCTTGTACATAGACGAGGAATATGAAATCTTTTGTTTGAAAACTTAAGTTAGCTATTAGC  280
YJCWR    AGGCCCAAAGTCTTGTACATAGACAAGGAATATGAAATCTTTTGTTTGAAAACTTAAGTTAGGTATTAGC  279
 
Teqing   ACGTGACATGAGCCTACACGTCTATATCAGTTTAGCAAAGCGTATAGTGTTAAGATTAATTCAAAATGAC  349
HJX74    ACGTGACATGAGCCTACACGTCTATATCAGTTTAGCAAAGCGTATAGTGTTAAGATTAATTCAAAATGAC  349
SN57     ACGTGACATGAGCCTACACGTCTATATCAGTTTAGCGAAGCGTATAGTGTTAAGATTAATTCAAAATGAC  349
NIV1     ACGTGACATGAGCCTACACGTCTATATCAGTTTAGCGAAGCGTATAGTGTTAAGATTAATTCAAAATGAC  349
NIV2     ACGTGACATGAGCCTACACGTCTATATCAGTTTAGCGAAGCGTATAGTGTTAAGATTAATTCAAAATGAC  349
RUF      ACGTGACATGAGCCTACACGTCTATATCAGTTTAGCGAAGCGTATAGTGTTAAGATTAATTCAAAATGAC  350
YJCWR    ACGTGACATGAGCCTACACGTCTATATCAGTTTAGCGAAGCGTATAGTGTTAAGATTAATTCAAAATGAC  349
 
Teqing   AACGACTCGTAAGGTTGAGAATGGGTTCTGACAATGGTCGGCTATGTACCTACAAAAGATCACAAAACAT  419
HJX74    AACGACTCGTAAGGTTGAGAATGGGTTCTGACAATGGTCGGCTATGTACCTACAAAAGATCACAAAACAT  419
SN57     AACGACTCGTAAGGTTGAGAATGGGTTCTGACAATGGTCGGCTATGTACCTACAAAAGATCACAAAACAT  419
NIV1     AACGACTCGTAAGGTTGAGAATGGGTTCTGACAATGGTCGGCTATGTACCTACAAAAGATCACAAAACAT  419
NIV2     AACGACTCGTAAGGTTGAGAATGGGTTCTGACAATGGTCGGCTATGTACCTACAAAAGATCACAAAACAT  419
RUF      AACGACTCGTAAGGTTGAGAATGGGTTCTGACAATGGTCGGCTATGTACCTACAAAAGATCACAAAACAT  420
YJCWR    AACAACTCGTAAGGTTGAGAATGGGTTCTGACAATGGTCGGCTATGTACCTACAAAAGATCACAAAACAT  419
 
Teqing   ATCTCGATCAATTCATTGTGATATTTAATGCCCCCAAAGCATATATATAAGTTTTCACATTTCCGCCGAA  489
HJX74    ATCTCGATCAATTCATTGTGATATTTAATGCCCCCAAAGCATATATATAAGTTTTCACATTTCCGCCGAA  489
SN57     ATCTCGACCAATTCATTGTGATATTTAATGCCCCCAAAGCATATATATAAGTTTTCACATTTCCGCCGAA  489
NIV1     ATCTCGACCAATTCATTGTGATATTTAATGCCCCCAAAGCATATATATAAGTTTTCACATTTCCGCCGAA  489
NIV2     ATCTCGACCAATTCATTGTGATATTTAATGCCCCCAAAGCATATATATAAGTTTTCACATTTCCGCCGAA  489
RUF      ATCTCGATCAATTCATTGTGATATTTAATGCCCCCAAAGCATATATATAAGTTTTCACATTTCCGCCGAA  490
YJCWR    ATCTCGATCAATTCATTGTGATATTTAATGCCCCCAAAGCAAATATATAAGTTTTCACATTTCCGCCGAA  489
 
Teqing   TTCATCCAGAAACACATAATCGTGGAAAAACTAAGCCTATTGGGTACCGCCGCCAACGCGCCGGCCACGC  559
HJX74    TTCATCCAGAAACACATAATCGTGGAAAAACTAAGCCTATTGGGTACCGCCGCCAACGCGCCGGCCACGC  559
SN57     TTCATCCAGAAACACATAATCGTGGAAAGACTAAGCCTATCAGGTACCGCCGCCACCGCGCCGGCCACGC  559
NIV1     TTCATCCAGAAACACATAATCGTGGAAAGACTAAGCCTATCAGGTACCGCCGCCACCGCGCCGGCCACGC  559
NIV2     TTCATCCAGAAACACATAATCGTGGAAAGACTAAGCCTATCAGGTACCGCCGCCACCGCGCCGGCCACGC  559
RUF      TTCATCCAGAAACACATAATCGTGGAAAGACTAAGCCTATCAGGTACCGCCGCCACCGCGCCGGCCACGC  560
YJCWR    TTCATCCAGAAACACATAATCGTGGAAAGACTAAGCCTATCGGGTACCGCCGCCACCGCGCCGGCCACGC  559
 
Teqing   GAGACCGGAAGCGCAGAAGATCCTGGCTATCCATGGACCACACAAGGCAGGGGCAGGCTCTTCCACAATC  629
HJX74    GAGACCGGAAGCGCAGAAGATCCTGGCTATCCATGGACCACACAAGGCAGGGGCAGGCTCTTCCACAATC  629
SN57     GAGACCGGAAGCGCAGAAGATCCTGGCTATCCATGGACCACACAAGGCAGGGGCAGGCTCTTCCACAATC  629
NIV1     GAGACCGGAAGCGCAGAAGATCCTGGCTATCCATGGACCACACAAGGCAGGGGCAGGCTCTTCCACAATC  629
NIV2     GAGACCGGAAGCGCAGAAGATCCTGGCTATCCATGGACCACACAAGGCAGGGGCAGGCTCTTCCACAATC  629
RUF      GAGACCGGAAGCGCAGAAGATCCTGGCTATCCATGGACCACACAAGGCAGGGGCAGGCTCTTCCACAATC  630
YJCWR    GAGACCGGAAGCGCAGAAGATCCTGGCTATCCATGGACCACACAAGGCAGGGGCAGGCTCTTCCACAATC  629
 
Teqing   CCGGGTCTGGTGACATGAGCCACAGTTGTGGCCCGAACAAAGTGACGTGTGTCGAGTCGAACGCCAGGC  698
HJX74    CCGGGTCTGGTGACATGAGCCACAGTTGTGGCCCGAACAAAGTGACGTGTGTCGAGTCGAACGCCAGGC  698
SN57     CCGGGTCTGGTGACATGAGCCACAGTTGTGGCCCGAACAAAGTGACGTGTGTCGAGTCGAACGCCAGGC  698
NIV1     CCGGGTCTGGTGACATGAGCCACAGTTGTGGCCCGAACAAAGTGACGTGTGTCGAGTCGAACGCCAGGC  698
NIV2     CCGGGTCTGGTGACATGAGCCACAGTTGTGGCCCGAACAAAGTGACGTGTGTCGAGTCGAACGCCAGGC  698
RUF      CCGGGTCTGGTGACATGAGCCACAGTTGTGGCCCGAACAAAGTGACGTGTGTCGAGTCGAACGCCAGGC  699
YJCWR    CCGGGTCTGGTGACATGAGCCACAGTTGTGGCCCGAACAAAGTGACGTGTGTCGAGTCGAACGCCAGGC  698
 
Additional file 12. Alignment of key promoter sequence of OsLG1
The red box shows the key SNP mutation in OsLG1. Teqing and YJCWR represent the Oslg1 and OsLG1 allele.
