# Supplementary material for: Substitution Mapping and Allelic Variations of the Domestication Genes from O. rufipogon and O. nivara
Source: Rice (N Y). 2023 Sep 5;16:38. doi: 10.1186/s12284-023-00655-y (PMC10480103; doi:10.1186/s12284-023-00655-y)
Supplement: Supplementary file 13 — Additional file 13: Genome sequence alignment of LABA1. [file 12284_2023_655_MOESM13_ESM.rtf]

HJX74   TGAGCAAAGCCATGCTGTAGTGGGATCTCTCTCCATCCATTCCTGCAGTGCCGCCACTGCTATAGCTGCG  70
SN54    TGAGCAAAGCCATGCTGTAGTGGGATCTCTCTCCATCCATTCCTGCAGTGCCGCCACTGCTATAGCTGCG  70
 
HJX74   AGAGCTATAGCTAGAAAAATATCAACGTAGGAAGTGGGAGGAGAAGTAGATGATGGATACAGATCACACT  140
SN54    AGAGCTATAGCTAGAAAAATATCAACGTAGGAAGTGGGAGGAGAAGTAGATGATGGATACAGATCACACT  140
 
HJX74   GAGATAATTAAGGAGGGAGAGGCAGTAGTGGAAGCCATGGCTCTACTCAGTCTCGGTTCAGGAGGATATG  210
SN54    GAGATAATTAAGGAGGGAGAGGCAGTAGTGGAAGCCATGGCTCTACTCAGTCTCGGTTCAGGAGGATATG  210
 
HJX74   CGTCTTCTGCGGGAGCAGCCAGGGCAAGAAGAAGAGCTACCAGGACGCAGCCGTTGAGCTTGGCAAGGAG  280
SN54    CGTCTTCTGCGGGAGCAGCCAGGGCAAGAAGAAGAGCTACCAGGACGCAGCCGTTGAGCTTGGCAAGGAG  280
 
HJX74   CTGGTACGTTCTACTATATTTCTCAATTCTCATATATGTAATCGTCTTGACAAAGCAGCTGCTTCATTTT  350
SN54    CTGGTACGTTCTACTATATTTCTCAATTCTCATATATGTAATCGTCTTGACAAAGCAGCTGCTTCATTTT  350
 
HJX74   TCAACCTTGATCATGACATCCTTTCTGAGATCTTGCTTCTTTAATTTTCTCTCGTTAATCAAAAACACCA  420
SN54    TCAACCTTGATCATGACATCCTTTCTGAGATCTTGCTTCTTTAATTTTCTCTCGTTAATCAAAAACACCA  420
 
HJX74   TGGCCATGGAGCTTTTTACTGATATTCAGCACAACGCGCCTGGTGATATGTAGGTATATAGAGAGTACCT  490
SN54    TGGCCATGGAGCTTTTTACTGATATTCAGCACAACGCGCCTGGTGATATGTAGGTATATAGAGAGTACCT  490
 
HJX74   GACAAGGTTTTGGGTTTCTTTTCAACGTCGTAATCTTGGAGTAATATAATTATCAAGCTGTGTAGACGTA  560
SN54    GACAAGGTTTTGGGTTTCTTTTCAACGTCGTAATCTTGGAGTAATATAATTATCAAGCTGTGTAGACGTA  560
 
HJX74   CACTTGCTGAACAAGGGCTAAAATTTATCTCTCTATATATTTAAGTGACAATATATGCGACATTACTGCT  630
SN54    CACTTGCTGAACAAGGGCTAAAATTTATCTCTCTATATATTTAAGTGACAATATATGCGACATTACTGCT  630
 
HJX74   AGCTGTAGACGTACTCTTGCAGTGCATGCAGCTTGTTCTTAGTAGAAGATGAGAGCATATGCATGAGCTG  700
SN54    AGCTGTAGACGTACTCTTGCAGTGCATGCAGCTTGTTCTTAGTAGAAGATGAGAGCATATGCATGAGCTG  700
 
HJX74   AGCACAGCATGTGTAGGTCTGAATTGAATTTCTGTATCATGCAGTAAAACCCACTTGTATTACAAAGGAG  770
SN54    AGCACAGCATGTGTAGGTCTGAATTGAATTTCTGTATCATGCAGTAAAACCCACTTGTATTACAAAGGAG  770
 
HJX74   AATGTCATGAGGGCTTGCACATGAGCTTATTAATTACTTCACATGAAAGACAATATGTCTGTCGGTGAAC  840
SN54    AATGTCATGAGGGCTTGCACATGAGCTTATTAATTACTTCACATGAAAGACAATATGTCTGTCGGTGAAC  840
 
HJX74   ATGGAATTTTATATTTATGCAGCTTGCATAGTTCCATAGAAAATGACTTACTCCCCAGGAACCTAGTACT  910
SN54    ATGGAATTTTATATTTATGCAGCTTGCATAGTTCCATAGAAAATGACTTACTCCCCAGGAACCTAGTACT  910
 
HJX74   GGATTAGGTTATGATCTTGTATAAGGCTGCTATATTTTGAAACGGAGGGAGTACTAGCCAGCAAGTTACT  980
SN54    GGATTAGGTTATGATCTTGTATAAGGCTGCTATATTTTGAAACGGAGGGAGTACTAGCCAGCAAGTTACT  980
 
HJX74   ACGCGCATTTGCATTTGTATGGTGACTTTTCGCCGGGATTTTCGGTGCTCAGCGCATTTTTCTCTATCCA  1050
SN54    ACGCGCATTTGCATTTGTATGGTGACTTTTCGCCGGGATTTTCGGTGCTCAGCGCATTTTTCTCTATCCA  1050
 
HJX74   TTTCGAGGCACGGTATAAATGCAAAAATGAAATAGACAAGTCGCATGGATCAGTCTTAAAGGAAACTATA  1120
SN54    TTTCGAGGCACGGTATAAATGCAAAAATGAAATAGACAAGTCGCATGGATCAGTCTTAAAGGAAACTATA  1120
 
HJX74   TATAGTGTGTACTGTATGTAGTAGCTAGACTACATATTCTAGATCACTTGATGATATATATTATAGAAGA  1190
SN54    TATAGTGTGTACTGTATGTAGTAGCTAGACTACATATTCTAGATCACTTGATGATATATATTATAGAAGA  1190
 
HJX74   CTAGATTCTAGAATGTGGAAATGCATCCCCACTCTCCATTATTATGGTTTCACCACAGGTAGCAAGGAAC  1260
SN54    CTAGATTCTAGAATGTGGAAATGCATCCCCACTCTCCATTATTATGGTTTCACCACAGGTAGCAAGGAAC  1260
 
HJX74   ATTGATCTAGTGTATGGTGGAGGAAGTGTGGGGCTCATGGGCCTGGTCTCTCAAGCTGTCTACAATGGAG  1330
SN54    ATTGATCTAGTGTATGGTGGAGGAAGTGTGGGGCTCATGGGCCTGGTCTCTCAAGCTGTCTACAATGGAG  1330
 
HJX74   GGAGGCATGTTATTGGGTATGTAAAAACGTAATAATTGTTGATCATTCTTCAGCACTGATACATGGAAAG  1400
SN54    GGAGGCATGTTATTGGGTATGTAAAAACGTAATAATTGTTGATCATTCTTCAGCACTGATACATGGAAAG  1400
 
HJX74   AATAACTCCGTATATAGTACACTTTGTAAGATGTCATCTTGTCATGACAATAGACTTGGATTATGGTTCC  1470
SN54    AATAACTCCGTATATAGTACACTTTGTAAGATGTCATCTTGTCATGACAATAGACTTGGATTATGGTTCC  1470
 
HJX74   TTTCCTCAGGTGCTACATGCTAGTACTAATATATAATTTGCCATTTTGTTGTCCCTATATACACGCAGGG  1540
SN54    TTTCCTCAGGTGCTACATGCTAGTACTAATATATAATTTGCCATTTTGTTGTCCCTATATACACGCAGGG  1540
 
HJX74   TGATTCCCAAGACTCTTATGCCTAGAGAGGTAAGCACGCTCATCTCCCTTCCAACAAGTCCTGACATAGT  1610
SN54    TGATTCCCAAGACTCTTATGCCTAGAGAGGTAAGCACGCTCATCTCCCTTCCAACAAGTCCTGACATAGT  1610
 
HJX74   TTATTCACATGCAACGATTCCCATGAGCTTGACATCATTGTTGCTGATCACCGGTTAAAATCTCATCATT  1680
SN54    TTATTCACATGCAACGATTCCCATGAGCTTGACATCATTGTTGCTGATCACCGGTTAAAATCTCATCATT  1680
 
HJX74   TTCATTGGAAATGTCACTTGAAAAAAAATCTAGTTAATACTTATTGATCAAGCATGTTAAGTATTTTGTA  1750
SN54    TTCATTGGAAATGTCACTTGAAAAAAAATCTAGTTAATACTTATTGATCAAGCATGTTAAGTATTTTGTA  1750
 
HJX74   AGCATGATAGTATCACTAGATGAGATTGCTTTTTAGAAAAAAGTCAGTAGCACTGATCTAAAATTATATC  1820
SN54    AGCATGATAGTATCACTAGATGAGATTGCTTTTTAGAAAAAAGTCAGTAGCACTGATCTAAAATTATATC  1820
 
HJX74   ACTTATAGGTATTAGAAAAACATATATATATGGTATGTGGATACATACTACTACTGGAAAATTATGCTTG  1890
SN54    ACTTATAGGTATTAGAAAAACATATATATATGGTATGTGGATACATACTACTACTGGAAAATTATGCTTG  1890
 
HJX74   TTCATTATCTTTCTTGAATCTTGATAGTGGAAAGAAGAATGCGATCTTATCTGCTTACAAAAGATCGCAT  1960
SN54    TTCATTATCTTTCTTGAATCTTGATAGTGGAAAGAAGAATGCGATCTTATCTGCTTACAAAAGATCGCAT  1960
 
HJX74   GGAACGTTCAAAGAAAATCTTGTTTTTCCTTTCCCTCGTAGATGAAATCCTGCTGATCTTGACTGCCGCT  2030
SN54    GGAACGTTCAAAGAAAATCTTGTTTTTCCTTTCCCTCGTAGATGAAATCCTGCTGATCTTGACTGCCGCT  2030
 
HJX74   TCTAGTGGCTGCCCCTTTGTTCTTAAGGCCTTGATCCTTTAAGCAGATGCTGACTTTATTAATCAGAAAG  2100
SN54    TCTAGTGGCTGCCCCTTTGTTCTTAAGGCCTTGATCCTTTAAGCAGATGCTGACTTTATTAATCAGAAAG  2100
 
HJX74   AAATGTCCCTCACATTTTCTTTTTGTCCCCCAACAACAGCAACATGATTGCCTCTCCTGAACCTTTGCTG  2170
SN54    AAATGTCCCTCACATTTTCTTTTTGTCCCCCAACAACAGCAACATGATTGCCTCTCCTGAACCTTTGCTG  2170
 
HJX74   CGTTGCTCTGATTAAACTATACATGCTACCACTCCTGTTAAACAGTGCTCATCCCCTTGCTCGTTGGCTA  2240
SN54    CGTTGCTCTGATTAAACTATACATGCTACCACTCCTGTTAAACAGTGCTCATCCCCTTGCTCGTTGGCTA  2240
 
HJX74   GTTTTTGCATGCTCTCTTACTTTTTGTGGAGCAGAAAGCTGCAGGGTGCTCCTGTGTCTGTGCGAGAGGC  2310
SN54    GTTTTTGCATGCTCTCTTACTTTTTGTGGAGCAGAAAGCTGCAGGGTGCTCCTGTGTCTGTGCGAGAGGC  2310
 
HJX74   TTTGTCACATACGCATGCGTGTTTGCTTGTGTGTGCAGATTACGGGTGAGACAGTAGGGGAGGTGAAAGC  2380
SN54    TTTGTCACATACGCATGCGTGTTTGCTTGTGTGTGCAGATTACGGGTGAGACAGTAGGGGAGGTGAAAGC  2380
 
HJX74   AGTGGCAGATATGCATCAGAGGAAGGCTGAGATGGCCAGGCAATCTGATGCGTTCATAGCACTGCCTGGT  2450
SN54    AGTGGCAGATATGCATCAGAGGAAGGCTGAGATGGCCAGGCAATCTGATGCGTTCATAGCACTGCCTGGT  2450
 
HJX74   TAGTCTCTCTCACCACCTGGATATATTTTTTTTAAGATAATGAAACCACCTGAATATATAGCTTCATCAA  2520
SN54    TAGTCTCTCTCACCACCTGGATATATTTTTTTTAAGATAATGAAACCACCTGAATATATAGCTTCATCAA  2520
 
HJX74   CTTGTGTTGAGTCGACATGTACTAATTTGCTTTACTTTTGTTGTCAATTGCCTTGTCATATTTTTTCCTT  2590
SN54    CTTGTGTTGAGTCGACATGTACTAATTTGCTTTACTTTTGTTGTCAATTGCCTTGTCATATTTTTTCCTT  2590
 
HJX74   TATCATGTTATGATACGTGGCTAGTGGAAGTGTGAGTGGAAAGTTGCGTAAAAGCAAGCACAAGCAGGGT  2660
SN54    TATCATGTTATGATACGTGGCTAGTGGAAGTGTGAGTGGAAAGTTGCGTAAAAGCAAGCACAAGCAGGGT  2660
 
HJX74   GCATGAGTTTGACTTTGACCTGCTAAATTGGCTTGCATGCATGCTGCTGCCGCCCAGTCGGTGCAGAATC  2730
SN54    GCATGAGTTTGACTTTGACCTGCTAAATTGGCTTGCATGCATGCTGCTGCCGCCCAGTCGGTGCAGAATC  2730
 
HJX74   CTTCTTTATACGCATGAGCTGGTCGATCAGATTGTCAGATACTCGGATAAGAGAGGACCTGTTGAGCCAC  2800
SN54    CTTCTTTATACGCATGAGCTGGTCGATCAGATTGTCAGATACTCGGATAAGAGAGGACCTGTTGAGCCAC  2800
 
HJX74   TTCGTGCTGATATTTTCAGTATCGTTCGTAGCTTATCTTTTTCAGTTATTTGATTCTTTTTCCTAATCAT  2870
SN54    TTCGTGCTGATATTTTCAGTATCGTTCGTAGCTTATCTTTTTCAGTTATTTGATTCTTTTTCCTAATCAT  2870
 
HJX74   CAGCCAACTGAATTTGTGATTCGCCCATCAGTCTTAAAAAAAAATTGTGATACGCCTTGTTAGCAAGGAC  2940
SN54    CAGCCAACTGAATTTGTGATTCGCCCATCAGTCTTAAAAAAAAATTGTGATACGCCTTGTTAGCAAGGAC  2940
 
HJX74   CAAATTTGGCCCATCCCCGAGGATGGGCTGGGAATTTTAACTAGATTCAGGACCATCCTAGTGAAATGGG  3010
SN54    CAAATTTGGCCCATCCCCGAGGATGGGCTGGGAATTTTAACTAGATTCAGGACCATCCTAGTGAAATGGG  3010
 
HJX74   CTAGTATTTTTTCTGCCTTCTCAAATCAAACCTGACACAACTTCATACTACTATGGATTTATGAGGTTCA  3080
SN54    CTAGTATTTTTTCTGCCTTCTCAAATCAAACCTGACACAACTTCATACTACTATGGATTTATGAGGTTCA  3080
 
HJX74   AACTCAGCCTAGAAATATTCTGAAGATAATGCAAACATCCGACAATTATACCCAGGCAGATGCTGTATCC  3150
SN54    AACTCAGCCTAGAAATATTCTGAAGATAATGCAAACATCCGACAATTATACCCAGGCAGATGCTGTATCC  3150
 
HJX74   GCAGATTCCCTCAATCAATACTCGTATTTCTGGAGGAGAGAGAGATCCAGAGCGAGCACTGTTCATTACT  3220
SN54    GCAGATTCCCTCAATCAATACTCGTATTTCTGGAGGAGAGAGAGATCCAGAGCGAGCACTGTTCATTACT  3220
 
HJX74   TAAATTTGTTTACTGCAGGTGGGTATGGAACACTTGAAGAGCTCCTGGAAGTAATTGCCTGGGCTCAGCT  3290
SN54    TAAATTTGTTTACTGCAGGTGGGTATGGAACACTTGAAGAGCTCCTGGAAGTAATTGCCTGGGCTCAGCT  3290
 
HJX74   CGGCATTCACGACAAGCCGGTACATACTGAAATAGTTCATGATCAGCTTTTGCACATGCAACATATGTAC  3360
SN54    CGGCATTCACGACAAGCCGGTACATACTGAAATAGTTCATGATCAGCTTTTGCACATGCAACATATGTAC  3360
 
HJX74   ACGCACTGATGAACAATGCACGTATATACACGGAGAGCCAAAACTTTTTTTTACCTTGGCTTAATGTGGA  3430
SN54    ACGCACTGATGAACAATGCACGTATATACACGGAGAGCCAAAACTTTTTTTTACCTTGGCTTAATGTGGA  3430
 
HJX74   CGATCGATGCTACGTACAGGTTGGCCTGCTAAATGTGGACGGCTACTACAACTCTCTGCTGTCGTTCATC  3500
SN54    CGATCGATGCTACGTACAGGTTGGCCTGCTAAATGTGGACGGCTACTACAACTCTCTGCTGTCGTTCATC  3500
 
HJX74   GATAAAGCTGTGGAGGAAGAGTTCATCAGCCCCTCTGCGCGCCATATCATCGTGTTAGCTCCAACACCAA  3570
SN54    GATAAAGCTGTGGAGGAAGAGTTCATCAGCCCCTCTGCGCGCCATATCATCGTGTTAGCTCCAACACCAA  3570
 
HJX74   AAGAACTTCTCGAGAAGCTAGAGGTGTATATACTTATATATATAATCTATCGATTTTTCTGATGCATCAT  3640
SN54    AAGAACTTCTCGAGAAGCTAGAGGTGTATATACTTATATATATAATCTATCGATTTTTCTGATGCATCAT  3640
 
HJX74   GGCACACTGCAAAGGACAGAAGAAAACGCCGGTTTCATCGGTGAATGAGAGATCGAGCTGAACTTTCCTC  3710
SN54    GGCACACTGCAAAGGACAGAAGAAAACGCCGGTTTCATCGGTGAATGAGAGATCGAGCTGAACTTTCCTC  3710
 
HJX74   TTGCTCGCATGCAGGCGTACTCCCCTCGGCATGACAAGGTCGTGCCGAAGATGCAGTGGGAGATGGAGAA  3780
SN54    TTGCTCGCATGCAGGCGTACTCCCCTCGGCATGACAAGGTCGTGCCGAAGATGCAGTGGGAGATGGAGAA  3780
 
HJX74   GATGAGCTACTGCAAGAGCTGCGAGATCCCTGGCCTGAAAGAAGGCAACAAGGCGACCATCCAAGCACAG  3850
SN54    GATGAGCTACTGCAAGAGCTGCGAGATCCCTGGCCTGAAAGAAGGCAACAAGGCGACCATCCAAGCACAG  3850
 
HJX74   CGAGGAAGCATGCTCTGAAATTTACTGTAGCACTAGCTAGCTATAGCTTAGCCAATGCGTGCAGCAAGAA  3920
SN54    CGAGGAAGCATGCTCTGAAATTTACTGTAGCACTAGCTAGCTATAGCTTAGCCAATGCGTGCAGCAAGAA  3920
 
HJX74   GATTCAAAACTTCTTGGTGCACATGAACTGCAACTTTTAATTCATGTAACTCGGTTCATTAGAAGGCAAT  3990
SN54    GATTCAAAACTTCTTGGTGCACATGAACTGCAACTTTTAATTCATGTAACTCGGTTCATTAGAAGGCAAT  3990
 
HJX74   TGATCACTGATCATGCAATTAGTCGTGTACGTAGCCCTAAGAATGAAATCATCAGTAAGATTTGTAAATC  4060
SN54    TGATCACTGATCATGCAATTAGTCGTGTACGTAGCCCTAAGAATGAAATCATCAGTAAGATTTGTAAATC  4060
 
HJX74   ACAGAGCTCCAGAGCAGCAGACTGACATAAAACGATATGTGGGCT----------------  4105
SN54    ACAGAGCTCCAGAGCAGCAGACTGACATAAAACGATATGTGGGCTAGATCGATCTCCTCCG  4121
 

Additional file 13. Genome sequence alignment of LABA1in HJX74 and SN54.
Note: Bases in blue background indicate exons of LABA1, red box shows the initiation codon and stop codon.
