# Supplementary material for: Substitution Mapping and Allelic Variations of the Domestication Genes from O. rufipogon and O. nivara
Source: Rice (N Y). 2023 Sep 5;16:38. doi: 10.1186/s12284-023-00655-y (PMC10480103; doi:10.1186/s12284-023-00655-y)
Supplement: Supplementary file 14 — Additional file 14: Genome sequence alignment of An-1. [file 12284_2023_655_MOESM14_ESM.rtf]

Nipponbare   ATGAACCCCACCACCGCCGCCGCCGCCGACCAACCATCCAAGCCCTCCGCCGCCGCCGCCGCCCGGAAGC  70
HJX74        ATGAACCCCACCACCGCCGCCGCCGCCGACCAACCATCCAAGCCCTCCGCCGCCGCCGCCGCCCGGAAGC  70
NIV1         ATGAACCCCACCACCGCCGCCGCCGCCGACCAACCATCCAAGCCCTCCGCCGCCGCCGCC---CGGAAGC  67
NIV2         ATGAACCCCACCACCGCCGCCGCCGCCGACCAACCATCCAAGCCCTCCGCCGCCGCCGCCGCCCGGAAGC  70
SR118        ATGAACCCCACCACCGCCGCCGCCGCCGACCAACCATCCAAGCCCTCCGCCGCCGCCGCC---CGGAAGC  67
RUF          ATGAACCCCACCACCGCCGCCGCCGCCGACCAACCATCCAAGCCCTCCGCCGCCGCCGCC---CGGAAGC  67
W1943        ATGAACCCCACCACCGCCGCCGCCGCCGACCAACCATCCAAGCCCTCCGCCGCCGCCGCC---CGGAAGC  67
 
Nipponbare   GCAAGTCGTCGGCGAAGCCCAAGGCCTCGTCCTCATCCTTACCCACGGTACGACACCATACTCCGACCAC  140
HJX74        GCAAGTCGTCGGCGAAGCCCAAGGCCTCGTCCTCATCCTTACCCACGGTACGACACCATACTCCGACCAC  140
NIV1         GCAAGTCGTCGGCGAAGCCCAAGGCCTCGTCCTCATCCTTACCCACGGTACGACACCATACTCCGACCAC  137
NIV2         GCAAGTCGTCGGCGAAGCCCAAGGCCTCGTCCTCATCCTTACCCACGGTACGACACCATACTCCGACCAC  140
SR118        GCAAGTCGTCGGCGAAGCCCAAGGCCTCGTCCTCATCCTTACCCACGGTACGACACCATACTCCGACCAC  137
RUF          GCAAGTCGTCGGCGAAGCCCAAGGCCTCGTCCTCATCCTTACCCACGGTACGACACCATACTCCGACCAC  137
W1943        GCAAGTCGTCGGCGAAGCCCAAGGCCTCGTCCTCATCCTTACCCACGGTACGACACCATACTCCGACCAC  137
 
Nipponbare   CGCTCTCCATCACTTCTCCGATCTCCACTGATCACCAACCCCATGCACAATGCAGGCCACGGCGACGACG  210
HJX74        CGCTCTCCATCACTTCTCCGATCTCCACTGATCACCAACCCCATGCACAATGCAGGCCACGGCGACGACG  210
NIV1         CGCTCTCCATCACTTCTCCGATCTCCACTGATCACCAACCCCATGCACAATGCAGGCCACGGCGACGACG  207
NIV2         CGCTCTCCATCACTTCTCCGATCTCCACTGATCACCAACCCCATGCACAATGCAGGCCACGGCGACGACG  210
SR118        CGCTCTCCATCACTTCTCCGATCTCCACTGATCACCAACCCCATGCACAATGCAGGCCACGGCGACGACG  207
RUF          CGCTCTCCATCACTTCTCCGATCTCCACTGATCACCAACCCCATGCACAATGCAGGCCACGGCGACGACG  207
W1943        CGCTCTCCATCACTTCTCCGATCTCCACTGATCACCAACCCCATGCACAATGCAGGCCACGGCGACGACG  207
 
Nipponbare   AACGCGAGCCCGAAGCGGTCCAAGGTCGCCGCCGGCGCCGGAGACGACGGCGACGCCGACGCCGACGCGG  280
HJX74        AACGCGAGCCCGAAGCGGTCCAAGGTCGCCGCCGGCGCCGGAGACGACGGCGACGCCGACGCCGACGCGG  280
NIV1         AACGCGAGCCCGAAGCGGTCCAAGGTCGCCGCCGGCGCCGGAGACGACGGCGACGGCGACGCCGACGCGG  277
NIV2         AACGCGAGCCCGAAGCGGTCCAAGGTCGCCGCCGGCGCCGGAGACGACGGCGACGGCGACGCCGACGCGG  280
SR118        AACGCGAGCCCGAAGCGGTCCAAGGTCGCCGCCGGCGCCGGAGACGACGGCGACGGCGACGCCGACGCGG  277
RUF          AACGCGAGCCCGAAGCGGTCCAAGGTCGCCGCCGGCGCCGGAGACGACGGCGACGGCGACGCCGACGCGG  277
W1943        AACGCGAGCCCGAAGCGGTCCAAGGTCGCCGCCGGCGCCGGAGACGACGGCGACGGCGACGCCGACGCGG  277
 
Nipponbare   CGGAGGAGAAGCCGGAGCCAGCCAAAGACTACATCCATGTGAGGGCGAGGCGGGGGCAAGCCACCGATAG  350
HJX74        CGGAGGAGAAGCCGGAGCCAGCCAAAGACTACATCCATGTGAGGGCGAGGCGGGGGCAAGCCACCGATAG  350
NIV1         CGGAGGAGAAGCCGGAGCCAGCCAAAGACTACATCCATGTGAGGGCGAGGCGGGGGCAAGCCACCGATAG  347
NIV2         CGGAGGAGAAGCCGGAGCCAGCCAAAGACTACATCCATGTGAGGGCGAGGCGGGGGCAAGCCACCGATAG  350
SR118        CGGAGGAGAAGCCGGAGCCAGCCAAAGACTACATCCATGTGAGGGCGAGGCGGGGGCAAGCCACCGATAG  347
RUF          CGGAGGAGAAGCCGGAGCCAGCCAAAGACTACATCCATGTGAGGGCGAGGCGGGGGCAAGCCACCGATAG  347
W1943        CGGAGGAGAAGCCGGAGCCAGCCAAAGACTACATCCATGTGAGGGCGAGGCGGGGGCAAGCCACCGATAG  347
 
Nipponbare   CCATAGCCTCGCCGAGAGGGTAATTAATCACCTAATTAATTAAATCCTAATTAGCTTTTTT-GCAAGTAC  419
HJX74        CCATAGCCTCGCCGAGAGGGTAATTAATCACCTAATTAATTAAATCCTAATTAGCTTTTTT-GCAAGTAC  419
NIV1         CCATAGCCTCGCCGAGAGGGTAATTAATCACCTAATTAATTAAATCCTAATTAGCTTTTTTTGCAAGTAC  417
NIV2         CCATAGCCTCGCCGAGAGGGTAATTAATCACCTAATTAATTAAATCCTAATTAGCTTTTTTTGCAAGTAC  420
SR118        CCATAGCCTCGCCGAGAGGGTAATTAATCACCTAATTAATTAAATCCTAATTAGCTTTTTT-GCAAGTAC  416
RUF          CCATAGCCTCGCCGAGAGGGTAATTAATCACCTAATTAATTAAATCCTAATTAGCTTTTTT-GCAAGTAC  416
W1943        CCATAGCCTCGCCGAGAGGGTAATTAATCACCTAATTAATTAAATCCTAATTAGCTTTTTTTGCAAGTAC  417
 
Nipponbare   TACACTAATCCCAAAATTAATCACAATGCGAGGGCACCCCCTAATTGCGCACCGTGTTACTAATTGCTCT  489
HJX74        TACACTAATCCCAAAATTAATCACAATGCGAGGGCACCCCCTAATTGCGCACCGTGTTACTAATTGCTCT  489
NIV1         TACACTAATCCCAAAATTAATCACAATGCGAGGGCACCCCCTAATTGCGCACCGTGTTACTAATTGCTCT  487
NIV2         TACACTAATCCCAAAATTAATCACAATGCGAGGGCACCCCCTAATTGCGCACCGTGTTACTAATTGCTCT  490
SR118        TACACTAATCCCAAAATTAATCACAATGCGAGGGCACCCCCTAATTGCGCACCGTGTTACTAATTGCTCT  486
RUF          TACACTAATCCCAAAATTAATCACAATGCGAGGGCACCCCCTAATTGCGCACCGTGTTACTAATTGCTCT  486
W1943        TACACTAATCCCAAAATTAATCACAATGCGAGGGCACCCCCTAATTGCGCACCGTGTTACTAATTGCTCT  487
 
Nipponbare   ACTTATTGAGTTGCAAGGACGCTTAATTATTATTAAAATTTTAATTAGCTTTGTGTTTTGTTTAATATTG  559
HJX74        ACTTATTGAGTTGCAAGGACGCTTAATTATTATTAAAATTTTAATTAGCTTTGTGTTTTGTTTAATATTG  559
NIV1         ACTTATTGAGTTGCAAGGACGCTTAATTATTATTAAAATTTTAATTAGCTTTGTGTTTTGTTTAATATTG  557
NIV2         ACTTATTGAGTTGCAAGGACGCTTAATTATTATTAAAATTTTAATTAGCTTTGTGTTTTGTTTAATATTG  560
SR118        ACTTATTGAGTTGCAAGGACGCTTAGTTATTATTAAAATTTTAATTAGCTTTGTGTTTTGTTTAATATTG  556
RUF          ACTTATTGAGTTGCAAGGACGCTTAGTTATTATTAAAATTTTAATTAGCTTTGTGTTTTGTTTAATATTG  556
W1943        ACTTATTGAGTTGCAAGGACGCTTAATTATTATTAAAATTTTAATTAGCTTTGTGTTTTGTTTAATATTG  557
 
Nipponbare   GTAGGTGAGGAGGGAGAGGATAAGCGAGAGGATGAAGCTTCTGCAGTCGCTCGTGCCAGGCTGCAACAAG  629
HJX74        GTAGGTGAGGAGGGAGAGGATAAGCGAGAGGATGAAGCTTCTGCAGTCGCTCGTGCCAGGCTGCAACAAG  629
NIV1         GTAGGTGAGGAGGGAGAGGATAAGCGAGAGGATGAAGCTTCTGCAGTCGCTCGTGCCAGGCTGCAACAAG  627
NIV2         GTAGGTGAGGAGGGAGAGGATAAGCGAGAGGATGAAGCTTCTGCAGTCGCTCGTGCCAGGCTGCAACAAG  630
SR118        GTAGGTGAGGAGGGAGAGGATAAGCGAGAGGATGAAGCTTCTGCAGTCGCTCGTGCCAGGCTGCAACAAG  626
RUF          GTAGGTGAGGAGGGAGAGGATAAGCGAGAGGATGAAGCTTCTGCAGTCGCTCGTGCCAGGCTGCAACAAG  626
W1943        GTAGGTGAGGAGGGAGAGGATAAGCGAGAGGATGAAGCTTCTGCAGTCGCTCGTGCCAGGCTGCAACAAG  627
 
Nipponbare   GTAGTATGAACACAAACACACTCCAGCGGCTAATCTCATTCTTAAATTTCCTCCAAATTCATGTATCCAA  699
HJX74        GTAGTATGAACACAAACACACTCCAGCGGCTAATCTCATTCTTAAATTTCCTCCAAATTCATGTATCCAA  699
NIV1         GTAGTATGAACACAAACACACTCCAGCGGCTAATCTCATTCTTAAATTTCCTCCAAATTCATGTATCCAA  697
NIV2         GTAGTATGAACACAAACACACTCCAGCGGCTAATCTCATTCTTAAATTTCCTCCAAATTCATGTATCCAA  700
SR118        GTAGTATGAACACAAACACACTCCAGCGGCTAATCTCATTCTTAAATTTCCTCCAAATTCATGTATCCAA  696
RUF          GTAGTATGAACACAAACACACTCCAGCGGCTAATCTCATTCTTAAATTTCCTCCAAATTCATGTATCCAA  696
W1943        GTAGTATGAACACAAACACACTCCAGCGGCTAATCTCATTCTTAAATTTCCTCCAAATTCATGTATCCAA  697
 
Nipponbare   ACAAGTGGCTTCTTTTTTTTCCCGCTCCAAATTACACTCAAAATTTAAAATTCTAGTTTTAGAGTTTAAT  769
HJX74        ACAAGTGGCTTCTTTTTTTTCCCGCTCCAAATTACACTCAAAATTTAAAATTCTAGTTTTAGAGTTTAAT  769
NIV1         ACAAGTGGCTTCTTTTTTTTCCCGCTCCAAATTACACTCAAAATTTAAAATTCTAGTTTTAGAGTTTAAT  767
NIV2         ACAAGTGGCTTCTTTTTTTTCCCGCTCCAAATTACACTCAAAATTTAAAATTCTAGTTTTAGAGTTTAAT  770
SR118        ACAAGTGGCTTCTTTTTTTTCCCGCTCCAAATTACACTCAAAATTTAAAATTCTAGTTTTAGAGTTTAAT  766
RUF          ACAAGTGGCTTCTTTTTTTTCCCGCTCCAAATTACACTCAAAATTTAAAATTCTAGTTTTAGAGTTTAAT  766
W1943        ACAAGTGGCTTCTTTTTTTTCCCGCTCCAAATTACACTCAAAATTTAAAATTCTAGTTTTAGAGTTTAAT  767
 
Nipponbare   TAATATCAAAAGCAGTTGTACTAATTTGTAATATTAGAATTCCGTTATACGGGGCAGTACTGAAAATTAG  839
HJX74        TAATATCAAAAGCAGTTGTACTAATTTGTAATATTAGAATTCCGTTATACGGGGCAGTACTGAAAATTAG  839
NIV1         TAATATCAAAAGCAGTTGTACTAATTTGTAATATTAGAATTCCGTTATACGGGGCAGTACTGAAAATTAG  837
NIV2         TAATATCAAAAGCAGTTGTACTAATTTGTAATATTAGAATTCCGTTATACGGGGCAGTACTGAAAATTAG  840
SR118        TAATATCAAAAGCAGTTGTACTAATTTGTAATATTAGAATTCCGTTATACGGGGCAGTACTGAAAATTAG  836
RUF          TAATATCAAAAGCAGTTGTACTAATTTGTAATATTAGAATTCCGTTATACGGGGCAGTACTGAAAATTAG  836
W1943        TAATATCAAAAGCAGTTGTACTAATTTGTAATATTAGAATTCCGTTATACGGGGCAGTACTGAAAATTAG  837
 
Nipponbare   TGGTCAAAAATGTATTTTCGAGGACGCGCATTAAGGGGGAATAGTAACCTAACGTACAAAACGGACTTGC  909
HJX74        TGGTCAAAAATGTATTTTCGAGGACGCGCATTAAGGGGGAATAGTAACCTAACGTACAAAACGGACTTGC  909
NIV1         TGGTCA--------TTTTCGAGGACGCGCATTAAGGGGGAATAGTAACCTAACGTACAAAACGGACTTGC  899
NIV2         TGGTCA--------TTTTCGAGGACGCGCATTAAGGGGGAATAGTAACCTAACGTACAAAACGGACTTGC  902
SR118        TGGTCA--------TTTTCGAGGACGCGCATTAAGGGGGAATAGTAACCTAACGTACAAAACGGACTTGC  898
RUF          TGGTCA--------TTTTCGAGGACGCGCATTAAGGGGGAATAGTAACCTAACGTACAAAACGGACTTGC  898
W1943        TGGTCAAAAATGTATTTTCGAGGACGCGCATTAAGGGGGAATAGTAACCTAACGTACAAAACGGACTTGC  907
 
Nipponbare   CTGTGTGGCAATAATTAAGTTTAATATATATACAATTACTTTAGTTTCAGTATTGCCTAGAGCTAGGAGT  979
HJX74        CTGTGTGGCAATAATTAAGTTTAATATATATACAATTACTTTAGTTTCAGTATTGCCTAGAGCTAGGAGT  979
NIV1         CTGTGTGGCAATAATTAAGTTTAATATATATACAATTACTTTAGTTTCAGTATTGCCTAGAGCTAGGAGT  969
NIV2         CTGTGTGGCAATAATTAAGTTTAATATATATACAATTACTTTAGTTTCAGTATTGCCTAGAGCTAGGAGT  972
SR118        CAGTGTGGCAATAATTAAGTTTAATATATATACAATTACTTTAGTTTCAGTATTGCCTAGAGCTAGGAGT  968
RUF          CAGTGTGGCAATAATTAAGTTTAATATATATACAATTACTTTAGTTTCAGTATTGCCTAGAGCTAGGAGT  968
W1943        CTGTGTGGCAATAATTAAGTTTAATATATATACAATTACTTTAGTTTCAGTATTGCCTAGAGCTAGGAGT  977
 
Nipponbare   TGGACTTTTTGCTACTGGGATAGATAAAATTAAGTAAAAAAATAATAATCTTTTTTGTACCGAAAGTGTG  1049
HJX74        TGGACTTTTTGCTACTGGGATAGATAAAATTAAGTAAAAAAATAATAATCTTTTTTGTACCGAAAGTGTG  1049
NIV1         TGGACTTTTTGCTACTAGGATAGATAAAATTAAGTAAAAAAATAATAATCTTTTTTGTACCGAAAGTGTG  1039
NIV2         TGGACTTTTTGCTACTGGGATAGATAAAATTAAGTAAAAAAATAATAATCTTTTTTGTACCGAAAGTGTG  1042
SR118        TGGACTTTTTGCTACTGGGATAGATAAAATTAAGTAAAAAAATAATAATCTTTTTTGTACCGAAAGTGTG  1038
RUF          TGGACTTTTTGCTACTGGGATAGATAAAATTAAGTAAAAAAATAATAATCTTTTTTGTACCGAAAGTGTG  1038
W1943        TGGACTTTTTGCTACTGGGATAGATAAAATTAAGTAAAAAAATAATAATCTTTTTTGTACCGAAAGTGTG  1047
 
Nipponbare   AGCACTAACTGCGGTGGTTAGTCTATGCAGCCAGTAGTAATTTTGACCGTAATGCATGTAAGCTACTAGT  1119
HJX74        AGCACTAACTGCGGTGGTTAGTCTATGCAGCCAGTAGTAATTTTGACCGTAATGCATGTAAGCTACTAGT  1119
NIV1         AGCACTAACTGCGGTGGTTAGTCTATGCAGCCAGTAGTAATTTTGACCGTAATGCATGTAAGCTACTAGT  1109
NIV2         AGCACTAACTGCGGTGGTTAGTCTATGCAGCCAGTAGTAATTTTGACCCTAATGCATGTAAGCTACTAGT  1112
SR118        AGCACTAACTGCGGTGGTTAGTCTATGCAGCCAGTAGTAATTTTGACCGTAATGCATGTAAGCTACTAGT  1108
RUF          AGCACTAACTGCGGTGGTTAGTCTATGCAGCCAGTAGTAATTTTGACCGTAATGCATGTAAGCTACTAGT  1108
W1943        AGCACTAACTGCGGTGGTTAGTCTATGCAGCCAGTAGTAATTTTGACCGTAATGCATGTAAGCTACTAGT  1117
 
Nipponbare   AGTACTCCTAAGTTAAGCTCCCAGTGGTCCAAGAGGATATAATAATAATTGATTAGTTAATAATCTGAGA  1189
HJX74        AGTACTCCTAAGTTAAGCTCCCAGTGGTCCAAGAGGATATAATAATAATTGATTAGTTAATAATCTGAGA  1189
NIV1         AGTACTCCTAAGTTAAGCTCCCAGTGGTCCAAGAGGATATAATAATAATTGATTAGTTAATAATCTGAGA  1179
NIV2         AGTACTCCTAAGTTAAGCTCCCAGTGGTCCAAGAGGA-ATAATAATAATTGATTAGTTAATAATCTGAGA  1181
SR118        AGTACTCCTAAGTTAAGCTCCCAGTGGTCCAAGAGGATATAATAATAATTGATTAGTTAATAATCTGAGA  1178
RUF          AGTACTCCTAAGTTAAGCTCCCAGTGGTCCAAGAGGATATAATAATAATTGATTAGTTAATAATCTGAGA  1178
W1943        AGTACTCCTAAGTTAAGCTCCCAGTGGTCCAAGAGGATATAATAATAATTGATTAGTTAATAATCTGAGA  1187
 
Nipponbare   GGCTCTTGCTCTGTACTTAAACTAATCAAACCGGCTAATCAATTGCAAGGTCCTGGTGGTTTAGGTGGTG  1259
HJX74        GGCTCTTGCTCTGTACTTAAACTAATCAAACCGGCTAATCAATTGCAAGGTCCTGGTGGTTTAGGTGGTG  1259
NIV1         GGCTCTTGCT--GTACTTAAACTAATCAAACCGGCTAATCAATTGCAAGGTCCTGGTGGTTTAGGTGGTG  1247
NIV2         GGCTCTTGCTCTGTACTTAAACTAATCAAACCGGCTAATCAATTGCAAGGTCCTGGTGGTTTAGGTGGTG  1251
SR118        GGCTCTTGCTCTGTACTTAAACTAATCAAACCGGCTAATCAATTGCAAGGTCCTGGTGGTTTAGGTGGTG  1248
RUF          GGCTCTTGCTCTGTACTTAAACTAATCAAACCGGCTAATCAATTGCAAGGTCCTGGTGGTTTAGGTGGTG  1248
W1943        GGCTCTTGCTCTGTACTTAAACTAATCAAACCGGCTAATCAATTGCAAGGTCCTGGTGGTTTAGGTGGTG  1257
 
Nipponbare   CGCACGATTATTTTAGTTGCCCCAATTATTGCCACGGTAGTGATGCCACCGCCGGTGGTCAAATTTGGGT  1329
HJX74        CGCACGATTATTTTAGTTGCCCCAATTATTGCCACGGTAGTGATGCCACCGCCGGTGGTCAAATTTGGGT  1329
NIV1         CGCACGATTATTTTAGTTGCCCCAATTATTGCCACGGTAGTGATGCCACCGCCGATGGTCAAATTTGGGT  1317
NIV2         CGCACGATTATTTTAGTTGCCCCAATTATTGCCACGGTAGTGATGCCACCGCCGGTGGTCAAATTTGGGT  1321
SR118        CGCACGATTATTTTAGTTGCCCCAATTATTGCCACGGTAGTGATGCCACCGCCGGTGGTCAAATTTGGGT  1318
RUF          CGCACGATTATTTTAGTTGCCCCAATTATTGCCACGGTAGTGATGCCACCGCCGGTGGTCAAATTTGGGT  1318
W1943        CGCACGATTATTTTAGTTGCCCCAATTATTGCCACGGTAGTGATGCCACCGCCGGTGGTCAAATTTGGGT  1327
 
Nipponbare   TGAATTTTAAATTTGGTTGATAACTTTTCTTACCTTTTTAAAATTTATTGCTGAGTTTTTTTCTAGATGA  1399
HJX74        TGAATTTTAAATTTGGTTGATAACTTTTCTTACCTTTTTAAAATTTATTGCTGAGTTTTTTTCTAGATGA  1399
NIV1         TGAATTTTAAATTTGGTTGATAACTTTTCTTACCTTTTTAAAAATTATTGCTGAGTTTTTTTCTAGATGA  1387
NIV2         TGAATTTTAAATTTGGTTGATAACTTTTCTTACCTTTTTAAAAATTATTGCTGAGTTTTTTTCTAGATGA  1391
SR118        TGAATTTTAAATTTGGTTGATAACTTTTCTTACCTTTTTAAAAATTATTGCTGAGTTTTTTTCTAGATGA  1388
RUF          TGAATTTTAAATTTGGTTGATAACTTTTCTTACCTTTTTAAAAATTATTGCTGAGTTTTTTTCTAGATGA  1388
W1943        TGAATTTTAAATTTGGTTGATAACTTTTCTTACCTTTTTAAAATTTATTGCTGAGTTTTTTTCTAGATGA  1397
 
Nipponbare   TTGTTTATTGTTTTTGTTTTGTTAATTAGTTAGCAAATAAATGTGAATGTTGCGGTGTGCAGATCACCGG  1469
HJX74        TTGTTTATTGTTTTTGTTTTGTTAATTAGTTAGCAAATAAATGTGAATGTTGCGGTGTGCAGATCACCGG  1469
NIV1         TTGTTTATTGTTTTTGTTTTGTTAATTAGTTAGCAAATAAATGTGAATGTTGCGGTGTGCAGATCACCGG  1457
NIV2         TTGTTTATTGTTTTTGTTTTGTTAATTAGTTAGCAAATAAATGTGAATGTTGCGGTGTGCAGATCACCGG  1461
SR118        TTGTTTATTGTTTTTGTTTTGCTAATTAGTTAGCAAATAAATGTGAATGTTGCGGTGTGCAGATCACCGG  1458
RUF          TTGTTTATTGTTTTTGTTTTGCTAATTAGTTAGCAAATAAATGTGAATGTTGCGGTGTGCAGATCACCGG  1458
W1943        TTGTTTATTGTTTTTGTTTTGTTAATTAGTTAGCAAATAAATGTGAATGTTGCGGTGTGCAGATCACCGG  1467
 
Nipponbare   CAAGGCTCTCATGCTGGACGAGATCATCAACTATGTGCAGTCGCTGCAGCGTCAGGTCGAGGTACCAATG  1539
HJX74        CAAGGCTCTCATGCTGGACGAGATCATCAACTATGTGCAGTCGCTGCAGCGTCAGGTCGAGGTACCAATG  1539
NIV1         CAAGGCTCTCATGCTGGACGAGATCATCAACTATGTGCAGTCGCTGCAGCGTCAGGTCGAGGTACCAATG  1527
NIV2         CAAGGCTCTCATGCTGGACGAGATCATCAACTATGTGCAGTCGCTGCAGCGTCAGGTCGAGGTACCAATG  1531
SR118        CAAGGCTCTCATGCTGGACGAGATCATCAACTATGTGCAGTCGCTGCAGCGTCAGGTCGAGGTACCAATG  1528
RUF          CAAGGCTCTCATGCTGGACGAGATCATCAACTATGTGCAGTCGCTGCAGCGTCAGGTCGAGGTACCAATG  1528
W1943        CAAGGCTCTCATGCTGGACGAGATCATCAACTATGTGCAGTCGCTGCAGCGTCAGGTCGAGGTACCAATG  1537
 
Nipponbare   CAAGCATTTGCTTTAATAGTGTGCTAAATGAACTTGTTTTAATGACAATAAATATGTTGGAGAAAAATGT  1609
HJX74        CAAGCATTTGCTTTAATAGTGTGCTAAATGAACTTGTTTTAATGACAATAAATATGTTGGAGAAAAATGT  1609
NIV1         CAAGCATTTGCTTTAATAGTGTGCTAAATGAACTTGTTTTAATGACAATAAATATGTTGGAGAAAAATGT  1597
NIV2         CAAGCATTTGCTTTAATAGTGTGCTAAATGAACTTGTTTTAATGACAATAAATATGTTGGAGAAAAATGT  1601
SR118        CAAGCATTTGCTTTAATAGTGTGCTAAATGAACTTGTTTTAATGACAATAAATATGTTGGAGAAAAATGT  1598
RUF          CAAGCATTTGCTTTAATAGTGTGCTAAATGAACTTGTTTTAATGACAATAAATATGTTGGAGAAAAATGT  1598
W1943        CAAGCATTTGCTTTAATAGTGTGCTAAATGAACTTGTTTTAATGACAATAAATATGTTGGAGAAAAATGT  1607
 
Nipponbare   TGTTGTGTTCTCCTTCTGTCGTCCTAGCTAGCTCTTATTTGAAGTTATGTATGAAATTAAGCCCAAAAGC  1679
HJX74        TGTTGTGTTCTCCTTCTGTCGTCCTAGCTAGCTCTTATTTGAAGTTATGTATGAAATTAAGCCCAAAAGC  1679
NIV1         TGTTGTGTTCTCCTTCTGTCGTCCTAGCTAGCTCTTATTTGAAGTTATGTATGAAATTAAGCCCAAAAGC  1667
NIV2         TGTTGTGTTCTCCTTCTGTCGTCCTAGCTAGCTCTTATTTGAAGTTATGTATGAAATTAAGCCCAAAAGC  1671
SR118        TGTTGTGTTCTCCTTCTGTCGTCCTAGCTAGCTCTTATTTGAAGTTATGTATGAAATTAAGCCCAAAAGC  1668
RUF          TGTTGTGTTCTCCTTCTGTCGTCCTAGCTAGCTCTTATTTGAAGTTATGTATGAAATTAAGCCCAAAAGC  1668
W1943        TGTTGTGTTCTCCTTCTGTCGTCCTAGCTAGCTCTTATTTGAAGTTATGTATGAAATTAAGCCCAAAAGC  1677
 
Nipponbare   TAAATTTTGACATTGGTGTGTCTCACAGTTTTTGTCCATGAAGTTGGCGACCATGAATCCTCAGCTGGAC  1749
HJX74        TAAATTTTGACATTGGTGTGTCTCACAGTTTTTGTCCATGAAGTTGGCGACCATGAATCCTCAGCTGGAC  1749
NIV1         TAAATTTTGACATTGGTGTGTCTCACAGTTTTTGTCCATGAAGTTGGCGACCATGAATCCTCAGCTGGAC  1737
NIV2         TAAATTTTGACATTGGTGTGTCTCACAGTTTTTGTCCATGAAGTTGGCGACCATGAATCCTCAGCTGGAC  1741
SR118        TAAATTTTGACATTGGTGTGTCTCACAGTTTTTGTCCATGAAGTTGGCGACCATGAATCCTCAGCTGGAC  1738
RUF          TAAATTTTGACATTGGTGTGTCTCACAGTTTTTGTCCATGAAGTTGGCGACCATGAATCCTCAGCTGGAC  1738
W1943        TAAATTTTGACATTGGTGTGTCTCACAGTTTTTGTCCATGAAGTTGGCGACCATGAATCCTCAGCTGGAC  1747
 
Nipponbare   TTTGACAGCCATTACATGCCTTCCAAAGATGTAAGTATAGCATCTGAAAACACTTTTATCTGATCTAGAG  1819
HJX74        TTTGACAGCCATTACATGCCTTCCAAAGATGTAAGTATAGCATCTGAAAACACTTTTATCTGATCTAGAG  1819
NIV1         TTTGACAGCCATTACATGCCTTCCAAAGATGTGAGTATAGCATCTGAAAACACTTTTATCTGATCTAGAG  1807
NIV2         TTTGACAGCCATTACATGCCTTCCAAAGATGTAAGTATAGCATCTGAAAACACTTTTATCTGATCTAGAG  1811
SR118        TTTGACAGCCATTACATGCCTTCCAAAGATGTAAGTATAGCATCTGAAAACACTTTTATCTGATCTAGAG  1808
RUF          TTTGACAGCCATTACATGCCTTCCAAAGATGTAAGTATAGCATCTGAAAACACTTTTATCTGATCTAGAG  1808
W1943        TTTGACAGCCATTACATGCCTTCCAAAGATGTAAGTATAGCATCTGAAAACACTTTTATCTGATCTAGAG  1817
 
Nipponbare   A----GACAGTTGACACAGAGTACTATTACGATATTGTCCCTCAATTTGCAAATGTTATATTCGCCGCAC  1885
HJX74        A----GACAGTTGACACAGAGTACTATTACGATATTGTCCCTCAATTTGCAAATGTTATATTCGCCGCAC  1885
NIV1         A----GACAGTTGACACAGAGTACTATTACGATATTGTCCCTCAATTTGCAAATGTTATATTCGCCGCAC  1873
NIV2         A----GACAGTTGACACAGAGTACTATTACGATATTGTCCCTCAATTTGCAAATGTTATATTCGCCGCAC  1877
SR118        ATCAAGACAGTTGACACAGAGTACTATTACGATATTGTCCCTCAATTTGCAAATGTTATATTCGCCGCAC  1878
RUF          ATCAAGACAGTTGACACAGAGTACTATTACGATATTGTCCCTCAATTTGCAAATGTTATATTCGCCGCAC  1878
W1943        A----GACAGTTGACACAGAGTACTATTACGATATTGTCCCTCAATTTGCAAATGTTATATTCGCCGCAC  1883
 
Nipponbare   TCGGGGTATCATTTTCAGATGAGCCATATGCCAGTACCCGCATACCCGTCAAGCGATCCGACCACCACCA  1955
HJX74        TCGGGGTATCATTTTCAGATGAGCCATATGCCAGTACCCGCATACCCGTCAAGCGATCCGACCACCACCA  1955
NIV1         TCGGGGTATCATTTTCAGATGAGCCATATGCCAGTACCCGCATACCCGTCAGGCGATCCAACCACCACCA  1943
NIV2         TCGGGGTATCATTTTCAGATGAGCCATATGCCAGTACCCGCATACCCGTCAGGCGATCCAACCACCACCA  1947
SR118        TCGGGGTATCATTTTCAGATGAGCCATATGCCAGTACCCGCATACCCGTCAGGCGATCCAACCACCACCA  1948
RUF          TCGGGGTATCATTTTCAGATGAGCCATATGCCAGTACCCGCATACCCGTCAGGCGATCCAACCACCACCA  1948
W1943        TCGGGGTATCATTTTCAGATGAGCCATATGCCAGTACCCGCATACCCGTCAAGCGATCCGACCACCACCA  1953
 
Nipponbare   CCGCGTTCTCCTACACCGGCTCACCCGCCACTGCTGATCCATTCACCGTCTACAACTGCTGGGAGCTCGA  2025
HJX74        CCGCGTTCTCCTACACCGGCTCACCCGCCACTGCTGATCCATTCACCGTCTACAACTGCTGGGAGCTCGA  2025
NIV1         CCGCGTTCTCCTACACCGGCTCACCCGCCACTGCTGATCCATTCACCGTCTACAACTGCTGGGAGCTCGA  2013
NIV2         CCGCGTTCTCCTACACCGGCTCACCCGCCACTGCTGATCCATTCACCGTCTACAACTGCTGGGAGCTCGA  2017
SR118        CCGCGTTCTCCTACACCGGCTCACCCGCCACTGCTGATCCATTCACCGTCTACAACTGCTGGGAGCTCGA  2018
RUF          CCGCGTTCTCCTACACCGGCTCACCCGCCACTGCTGATCCATTCACCGTCTACAACTGCTGGGAGCTCGA  2018
W1943        CCGCGTTCTCCTACACCGGCTCACCCGCCACTGCTGATCCATTCACCGTCTACAACTGCTGGGAGCTCGA  2023
 
Nipponbare   CCTCCACACCGCTATGCAAATGGGAGCCACCACCGGACTCAGCCAAGACGGTCCAATCGCAACGATGGCA  2095
HJX74        CCTCCACACCGCTATGCAAATGGGAGCCACCACCGGACTCAGCCAAGACGGTCCAATCGCAACGATGGCA  2095
NIV1         CCTCCACACCGCTATGCAAATGGGAGCCACCCCCGGACTCAGCCAAGACGGTCCAATCGCAACGATGGCA  2083
NIV2         CCTCCACACCGCTATGCAAATGGGAGCCACCCCCGGACTCAGCCAAGACGTTCCAATCGCAACGATGGCA  2087
SR118        CCTCCACACCGCTATGCAAATGGGAGCCACCCCCGGACTCAGCCAAGACGGTCCAATCGCAACGATGGCA  2088
RUF          CCTCCACACCGCTATGCAAATGGGAGCCACCCCCGGACTCAGCCAAGACGGTCCAATCGCAACGATGGCA  2088
W1943        CCTCCACACCGCTATGCAAATGGGAGCCACCACCGGACTCAGCCAAGACGGTCCAATCGCAACGATGGCA  2093
 
Nipponbare   CCCTCTCCCTCGCCATTGCCGCACCATCCTCCTCTTCACGGCTTCTACGGTAAGTGAAATCGAACCACCA  2165
HJX74        CCCTCTCCCTCGCCATTGCCGCACCATCCTCCTCTTCACGGCTTCTACGGTAAGTGAAATCGAACCACCA  2165
NIV1         CCCTCTCCCTCGCCATTGCCGCACCATCCTCCTCTTCACGGCTTCTACGGTAAGTGAAATCGAACCACCA  2153
NIV2         CCCTCTCCCTCGCCATTGCCGCACCATCCTCCTCTTCACGGCTTCTACGGTAAGTGAAATCAAACCACCA  2157
SR118        CCCTCTCCCTCGCCATTGCCGCACCATCCTCCTCTTCACGGCTTCTACGGTAAGTGAAATCGAACCACCA  2158
RUF          CCCTCTCCCTCGCCATTGCCGCACCATCCTCCTCTTCACGGCTTCTACGGTAAGTGAAATCGAACCACCA  2158
W1943        CCCTCTCCCTCGCCATTGCCGCACCATCCTCCTCTTCACGGCTTCTACGGTAAGTGAAATCGAACCACCA  2163
 
Nipponbare   CATCTCCTTACATCCCTAACAAATTATACATGAATTTTTAAAAATACTCAATTTTTTTTTTAAAAAAA-G  2234
HJX74        CATCTCCTTACATCCCTAACAAATTATACATGAATTTTTAAAAATACTCAATTTTTTTTTAAAAAAAA-G  2234
NIV1         CATCTCCTTACATCCCTAACAAATTATACATGAATTTTTAAAAATACTCAATTTTTTTTTAAAAAAAA-G  2222
NIV2         CATCTCCTTACATCCCTAACAAATTATACATGAATTTTTAAAAATACTCA---TTTTTTTAAAAAAAA-G  2223
SR118        CATCTCCTTACATCCCTAACAAATTATACATGAATTTTTAAAAATACTCAATTTTTTTTTAAAAAAAA-G  2227
RUF          CATCTCCTTACATCCCTAACAAATTATACATGAATTTTTAAAAATACTCAATTTTTTTTTAAAAAAAA-G  2227
W1943        CATCTCCTTACATCCCTAACAAATTATACATGAATTTTTAAAAATACTCAATTTTTTTTTAAAAAAAAAG  2233
 
Nipponbare   TTCAAAAATAAGATTATTTTTTTTACTGGACTACAGTAGTGGAGTGGTGGTGGTCATACTACTCCAGTAC  2304
HJX74        TTCAAAAATAAGATTATTTTTTTTACTGGACTACAGTAGTGGAGTGGTGGTGGTCATACTACTCCAGTAC  2304
NIV1         TTCAAAAATAAGATTATTTTTTT-ACTGGACTACAGTAGTGGAGTGGTGGTGGTCATACTACTCCAGTAC  2291
NIV2         TTCAAAAATAAGATTATTTTTTTTACTGGACTACAGTAGTGGAGTTGCGGTGGTCATACTACTCCAGTAC  2293
SR118        TTCAAAAATAAGATTATTTTTTTTACTGGACTACAGTAGTGGAGTGGTGGTGGTCATACTACTCCAGTAC  2297
RUF          TTCAAAAATAAGATTATTTTTTTTACTGGACTACAGTAGTGGAGTGGTGGTGGTCATACTACTCCAGTAC  2297
W1943        TTCAAAAATAAGATTATTTTTTTTACTGGACTACAGTAGTGGAGTGGTGGTGGTCATACTACTCCAGTAC  2303
 
Nipponbare   GGTAGGTTTGTTTGTCCAAGTTTGTTGAGTTTCGCTGTTGGTGGTAATTGGGCGCAGGGGGGCAGCAGCA  2374
HJX74        GGTAGGTTTGTTTGTCCAAGTTTGTTGAGTTTCGCTGTTGGTGGTAATTGGGCGCAGGGGGGCAGCAGCA  2374
NIV1         GGTAGGTTTGTTTGTCCAAGTTTGTTGAGTTTCGCTGTTGGTGGTAATTGGGCGCAGGGGGGCAGCAGCA  2361
NIV2         GGTAGGTTTGTTTGTCCAAGTTTGTTGAGTTTCGCTGTTGGTGGTAATTGGGCGCAGGGGGGCAGCAGCA  2363
SR118        GGTAGGTTTGTTTGTCCAAGTTTGTTGAGTTTCGCTGTTGGTGGTAATTGGGCGCAGGGGGGCAGCAGCA  2367
RUF          GGTAGGTTTGTTTGTCCAAGTTTGTTGAGTTTCGCTGTTGGTGGTAATTGGGCGCAGGGGGGCAGCAGCA  2367
W1943        GGTAGGTTTGTTTGTCCAAGTTTGTTGAGTTTCGCTGTTGGTGGTAATTGGGCGCAGGGGGGCAGCAGCA  2373
 
Nipponbare   GCAGGGGACGACAGTAAACCACATGAAGGCCGAGCCATAA  2414
HJX74        GCAGGGGACGACAGTAAACCACATGAAGGCCGAGCCATAA  2414
NIV1         GCAGGGGACGACAGTAAACCACATGAAGGCCGAGCCATAA  2401
NIV2         GCAGGGGACGACAGTAAACCACATGAAGGCCGAGCCATAA  2403
SR118        GCAGGGGACGACAGTAAACCACATGAAGGCCGAGCCATAA  2407
RUF          GCAGGGGACGACAGTAAACCACATGAAGGCCGAGCCATAA  2407
W1943        GCAGGGGACGACAGTAAACCACATGAAGGCCGAGCCATAA  2413
 
Additional file 14. Genome sequence alignment of An-1.
Nipponbare and W1943 means the allele of an-1 and An-1.
