# Supplementary material for: Substitution Mapping and Allelic Variations of the Domestication Genes from O. rufipogon and O. nivara
Source: Rice (N Y). 2023 Sep 5;16:38. doi: 10.1186/s12284-023-00655-y (PMC10480103; doi:10.1186/s12284-023-00655-y)
Supplement: Supplementary file 15 — Additional file 15: Amino acid sequence alignment of An-1. [file 12284_2023_655_MOESM15_ESM.rtf]

Nipponbare   MNPTTAAAADQPSKPSAAAAARKRKSSAKPKASSSSLPTATATTNASPKRSKVAAGAGDDGDADADAAEE  70
HJX74        MNPTTAAAADQPSKPSAAAAARKRKSSAKPKASSSSLPTATATTNASPKRSKVAAGAGDDGDADADAAEE  70
NIV1         MNPTTAAAADQPSKPS-AAAARKRKSSAKPKASSSSLPTATATTNASPKRSKVAAGAGDDGDGDADAAEE  69
NIV2         MNPTTAAAADQPSKPSAAAAARKRKSSAKPKASSSSLPTATATTNASPKRSKVAAGAGDDGDGDADAAEE  70
SR118        MNPTTAAAADQPSKPS-AAAARKRKSSAKPKASSSSLPTATATTNASPKRSKVAAGAGDDGDGDADAAEE  69
RUF          MNPTTAAAADQPSKPS-AAAARKRKSSAKPKASSSSLPTATATTNASPKRSKVAAGAGDDGDGDADAAEE  69
W1943        MNPTTAAAADQPSKPS-AAAARKRKSSAKPKASSSSLPTATATTNASPKRSKVAAGAGDDGDGDADAAEE  69
 
Nipponbare   KPEPAKDYIHVRARRGQATDSHSLAERVRRERISERMKLLQSLVPGCNKITGKALMLDEIINYVQSLQRQ  140
HJX74        KPEPAKDYIHVRARRGQATDSHSLAERVRRERISERMKLLQSLVPGCNKITGKALMLDEIINYVQSLQRQ  140
NIV1         KPEPAKDYIHVRARRGQATDSHSLAERVRRERISERMKLLQSLVPGCNKITGKALMLDEIINYVQSLQRQ  139
NIV2         KPEPAKDYIHVRARRGQATDSHSLAERVRRERISERMKLLQSLVPGCNKITGKALMLDEIINYVQSLQRQ  140
SR118        KPEPAKDYIHVRARRGQATDSHSLAERVRRERISERMKLLQSLVPGCNKITGKALMLDEIINYVQSLQRQ  139
RUF          KPEPAKDYIHVRARRGQATDSHSLAERVRRERISERMKLLQSLVPGCNKITGKALMLDEIINYVQSLQRQ  139
W1943        KPEPAKDYIHVRARRGQATDSHSLAERVRRERISERMKLLQSLVPGCNKITGKALMLDEIINYVQSLQRQ  139
 
Nipponbare   VEFLSMKLATMNPQLDFDSHYMPSKDMSHMPVPAYPSSDPTTTTAFSYTGSPATADPFTVYNCWELDLHT  210
HJX74        VEFLSMKLATMNPQLDFDSHYMPSKDMSHMPVPAYPSSDPTTTTAFSYTGSPATADPFTVYNCWELDLHT  210
NIV1         VEFLSMKLATMNPQLDFDSHYMPSKDMSHMPVPAYPSGDPTTTTAFSYTGSPATADPFTVYNCWELDLHT  209
NIV2         VEFLSMKLATMNPQLDFDSHYMPSKDMSHMPVPAYPSGDPTTTTAFSYTGSPATADPFTVYNCWELDLHT  210
SR118        VEFLSMKLATMNPQLDFDSHYMPSKDMSHMPVPAYPSGDPTTTTAFSYTGSPATADPFTVYNCWELDLHT  209
RUF          VEFLSMKLATMNPQLDFDSHYMPSKDMSHMPVPAYPSGDPTTTTAFSYTGSPATADPFTVYNCWELDLHT  209
W1943        VEFLSMKLATMNPQLDFDSHYMPSKDMSHMPVPAYPSSDPTTTTAFSYTGSPATADPFTVYNCWELDLHT  209
 
Nipponbare   AMQMGATTGLSQDGPIATMAPSPSPLPHHPPLHGFYGGQQQQGTTVNHMKAEP  263
HJX74        AMQMGATTGLSQDGPIATMAPSPSPLPHHPPLHGFYGGQQQQGTTVNHMKAEP  263
NIV1         AMQMGATPGLSQDGPIATMAPSPSPLPHHPPLHGFYGGQQQQGTTVNHMKAEP  262
NIV2         AMQMGATPGLSQDVPIATMAPSPSPLPHHPPLHGFYGGQQQQGTTVNHMKAEP  263
SR118        AMQMGATPGLSQDGPIATMAPSPSPLPHHPPLHGFYGGQQQQGTTVNHMKAEP  262
RUF          AMQMGATPGLSQDGPIATMAPSPSPLPHHPPLHGFYGGQQQQGTTVNHMKAEP  262
W1943        AMQMGATTGLSQDGPIATMAPSPSPLPHHPPLHGFYGGQQQQGTTVNHMKAEP  262
 
Additional file 15. Amino acid sequence alignment of An-1.
