# Supplementary material for: Substitution Mapping and Allelic Variations of the Domestication Genes from O. rufipogon and O. nivara
Source: Rice (N Y). 2023 Sep 5;16:38. doi: 10.1186/s12284-023-00655-y (PMC10480103; doi:10.1186/s12284-023-00655-y)
Supplement: Supplementary file 16 — Additional file 16: Primers for gene amplification. [file 12284_2023_655_MOESM16_ESM.docx]

| Gene | Forward primer (5'-3') | Reverse primer (5'-3') |
| --- | --- | --- |
| *LABA1* | CCGGAAGAGATCAACTAACAGAGAC | GGCGGCAGGGACTATTTCTGCAAT |
| *RC*-1 | TCCTTTGTCGACACATGTGGAGGCC | CCTGGATACCAGCACTCTGCCATTC |
| *RC*-2 | GTGTGCTTTGCATGTCTTCAGGTTA | CCTTCACCTGCGCCAACCAATTCAC |
| *SH4* | AGGCGTTGTGCATGCAATGCACCGT | GATGAGCAGTGTCAGGTCTGTCAC |
| *PROG1* | ATCATGATTCGCAGCTTGCAAT | CTCGGATTCGGAAATAACTAGC |
| *TIG1* | GCATAAAGCTATGGGTATGGCC | ACAAGCCAAACCGGTATTGATAA |
| *OsLG1* | CCTAACCCTGGCCGGTCGATAT | CTAGCCATGTGCAATTGCACGAA |
| Promoter of *OsLG1* | GTGCGGAATTCGAATTAGACCG | TGCCAGTGCAATGCATGTGTGC |
| *AN-1* | CCAGCACACACAAAGGTCACACAGCTCC | GGCTGTCCCGTGTCTCCAGCAGCAAGT |

**Additional file 16. Primers for gene amplification**
